# Employing computational tools to design a multi-epitope vaccine targeting human immunodeficiency virus-1 (HIV-1)

**DOI:** 10.1186/s12864-023-09330-4

**Published:** 2023-05-24

**Authors:** Hamza Sher, Hafsa Sharif, Tahreem Zaheer, Sarmad Ahmad Khan, Amjad Ali, Hasnain Javed, Aneela Javed

**Affiliations:** 1 Atta Ur Rahman School of Applied Biosciences, National University of Sciences and Technology, Islamabad, Pakistan; 2 German Cancer Research Center (DFKZ), German Cancer Research Consortium (DKTK), Heidelberg, Germany; 3 Advanced Diagnostic Lab BSL-3, Punjab AIDS Control Program, Primary and Secondary Healthcare Department, Government of the Punjab, Lahore, Pakistan

**Keywords:** Immuno-informatics, Computational biology, Human immunodeficiency virus, Immunity, Vaccinology, Acquired immunodeficiency syndrome, Bioinformatics, Toll like receptor-3

## Abstract

**Background:**

Despite being in the 21^st^ century, the world has still not been able to vanquish the global AIDS epidemic, and the only foreseeable solution seems to be a safe and effective vaccine. Unfortunately, vaccine trials so far have returned unfruitful results, possibly due to their inability to induce effective cellular, humoral and innate immune responses. The current study aims to tackle these limitations and propose the desired vaccine utilizing immunoinformatic approaches that have returned promising results in designing vaccines against various rapidly mutating organisms. For this, all polyprotein and protein sequences of HIV-1 were retrieved from the LANL (Los Alamos National Laboratory) database. The consensus sequence was generated after alignment and used to predict epitopes. Conserved, antigenic, non-allergenic, T-cell inducing, B-cell inducing, IFN-ɣ inducing, non-human homologous epitopes were selected and combined to propose two vaccine constructs i.e., HIV-1a (without adjuvant) and HIV-1b (with adjuvant).

**Results:**

HIV-1a and HIV-1b were subjected to antigenicity, allergenicity, structural quality analysis, immune simulations, and MD (molecular dynamics) simulations. Both proposed multi-epitope vaccines were found to be antigenic, non-allergenic, stable, and induce cellular, humoral, and innate immune responses. TLR-3 docking and *in-silico* cloning of both constructs were also performed.

**Conclusion:**

Our results indicate HIV-1b to be more promising than HIV-1a; experimental validations can confirm the efficacy and safety of both constructs and *in-vivo* efficacy in animal models.

**Graphical Abstract:**

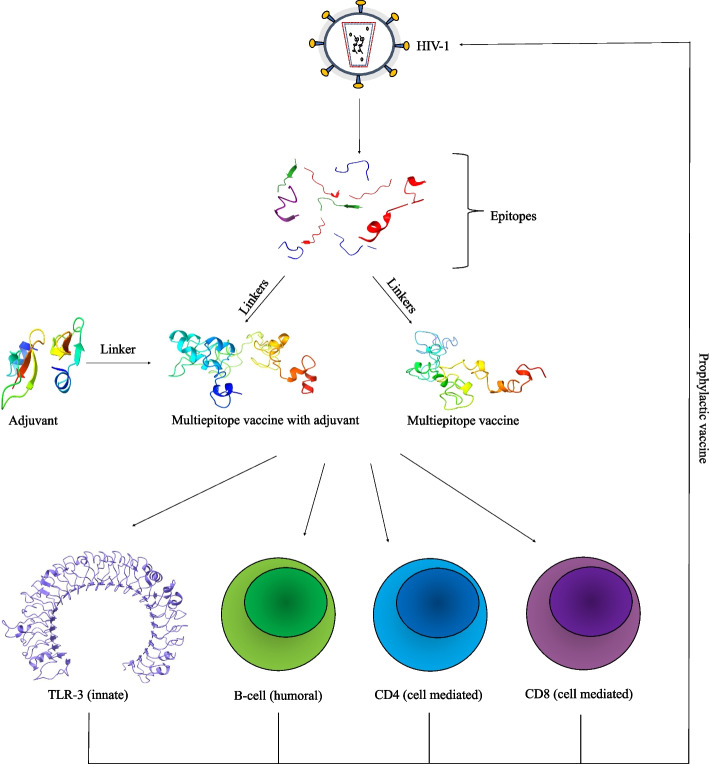

**Supplementary Information:**

The online version contains supplementary material available at 10.1186/s12864-023-09330-4.

## Background

An estimated 1.7 million people were infected with the Human Immunodeficiency Virus (HIV) in 2019, and despite the availability of effective treatments, the death toll was 690,000 [1]. Although these figures were 23% less than those reported in 2010 [1], and besides the availability of many successful drugs such as dolutegravir, efavirenz, and ritonavir [2], it is still believed that a successful vaccine will be required to eradicate Acquired Immunodeficiency Syndrome (AIDS) (caused by HIV infection) completely from the human population [3]. The importance of vaccination is highlighted by the present COVID pandemic, where only an effective vaccine has helped significantly reduce the rate of infection rate and decrease COVID-19 related morbidity and mortality [4]. The inadequate management of HIV infections has been mainly attributed to the lack of accessibility, expensiveness, and the inability of drugs. Apart from these factors, drug resistance in patients receiving antiretroviral therapy (ART) has also played a major role in the global expansion of HIV infections [5]. Out of two types of HIV (HIV-1 and HIV-2), HIV-1 is the most prevalent, and potent, and is considered the main reason for AIDS progression around the globe [6].

A subset of HIV infected patients has the ability to remain asymptomatic and maintain high levels of CD4 + cell count for many years without administration of antiretroviral therapy, known as elite controllers [7]. Analysis of these elite controllers demonstrated an abundance of CD4 + and CD8 + cells secreting Th1 promoting interferon-gamma (IFN-ɣ) in these patients [8]. Non-neutralizing antibodies are also seen to be effective against HIV-1 infection but so far no immunological correlates have been found, so the induction of broadly neutralizing antibodies is an important aspect of protection against AIDS [9]. Moreover, induction of both CD4 + and CD8 + cells have proven to be effective against HIV infection [10].

Unfortunately, no effective vaccine is available so far against HIV-1 and the most successful vaccine RV144 provided only 31.2% protection against this deadly microbe [11]. This unavailability of any effective treatment is attributed to the rapid mutation rate of HIV [11], the inability of vaccines to induce both cellular and helper T-cell responses like in the EP HIV-1090 vaccine [10], and the failure to induce broadly neutralizing antibodies [9].

An ideal vaccine should target conserved regions of the virus, induce B-cell, CD4 + T-cell, CD8 + T-cell, and appropriate cytokine response such as IFN-ɣ response, contain an appropriate adjuvant to enhance immunity against HIV-1, and should be able to activate an appropriate innate response. The current study aims to design a multi-epitope vaccine that targets conserved regions of HIV-1, can activate B-cells, CD4 + T-cells, and CD8 + T-cells, can induce appropriate proinflammatory cytokines such as IFN-ɣ, include an appropriate adjuvant such as human β-defensin-3 (HBD-3) [12], and simulate innate response through appropriate TLR docking, such as TLR-3 [13].

In order to design the vaccine with the aforementioned characteristics, the retrieved HIV-1 sequences were aligned to generate a consensus sequence. CD4 + T-cell, CD8 + T-cell, and B-cell epitopes were predicted, and conserved T- cell epitopes overlapping B-cell epitopes were selected. Selected epitopes were filtered for their ability to induce IFN-ɣ. Only antigenic and non-toxic epitopes were filtered for their homology with human sequences. After rigorous analysis of epitope-epitope interactions, non-human homolog epitopes were joined with an appropriate linker, and two constructs were generated, without adjuvant (HIV-1a) and with HBD-3 adjuvant (HIV-1b). The tertiary and secondary structures of both vaccines were designed using various computational tools. Moreover, protein quality analysis, allergenicity analysis, immune simulations, and Molecular Dynamics (MD) simulation were also performed. Molecular docking of both constructs was also performed with TLR-3. Finally, codon optimization and *in-silico* cloning of both constructs were performed.

## Results

### Retrieval of sequences and consensus sequence generation

HIV-1a proteins (VIF, VPR, TAT, REV, VPU, NEF) and polyproteins (GAG, POL, ENV) sequences were downloaded and then subjected to multiple sequence alignment. Consensus sequences were then generated for each protein and polyprotein, and, their conservancy with respective reference sequences (downloaded from NCBI) was analyzed. Details of this analysis are summarized in Supplementary Table S1. This analysis demonstrates that POL polyprotein is most conserved while VPU protein is least conserved.

### Epitope mapping

A total of 11 antigenic, non-toxic, and non-human homologous epitopes were shortlisted that can induce T-cell and B-cell response, moreover, they were also able to induce IFN-ɣ. Details of selected epitopes such as their sequence, symbol that will be used throughout this paper, respective protein they are derived from, ability to induce either cytotoxic or helper T-cell response, conservancy, antigenicity, E-value, and toxicity are mentioned in Table 1. Furthermore, Supplementary Table S2 depicts details of HLA class I and HLA class II molecules targeted by these selected epitopes. Population coverage analysis of the selected epitopes also depicts that they have 97.83% coverage in global population.

**Table 1:** Statistics of selected epitopes

**Sr. No**	**Epitopes**	**Symbol**	**Protein**	**Position**	**HLA-1/HLA-2**	**Antigenicity**	**BLASTp (E-score)**	**Toxicity (SVM value)**	**Conservancy**
**1**	APRKKGCWK	NC1	Nucleocapsid	30–38	HLA-1	0.6832	28	-0.48	83.88
**2**	PRKKGCWKC	NC2	Nucleocapsid	31–39	HLA-1	1.4065	55	-0.26	83.94
**3**	RKKGCWKCG	NC3	Nucleocapsid	32–40	HLA-1	1.5171	55	-0.1	83.94
**4**	PLTEEKIKA	RT1	Reverse Transcriptase	25–33	HLA-1	0.7833	13	-1.4	92.85
**5**	KRTQDFWEV	RT2	Reverse Transcriptase	82–90	HLA-1	1.1553	1.6	-1.49	91.78
**6**	RTQDFWEVQ	RT3	Reverse Transcriptase	83–91	HLA-1	1.421	1.6	-1.61	91.86
**7**	TQDFWEVQL	RT4	Reverse Transcriptase	84–92	HLA-1	0.716	1.6	-1.79	95.45
**8**	AHKGIGGNE	RT5	Reverse Transcriptase	538–546	HLA-1	0.6955	78	-1.05	98.21
**9**	FRVYYRDSR	Int1	Integrase	223–231	Both	0.8423	6.7	-1.36	88.12
**10**	VYYRDSRDP	Int2	Integrase	225–233	HLA-2	0.7874	1.2	-0.86	86.67
**11**	ERAEDSGNE	VPU1	VPU	48–56	HLA-1	0.6154	4.7	-1.14	82.84

Symbols adapted, parent proteins, reference protein positions, Targeted HLA type, antigenicity, BLASTp analysis with human proteome, toxicity, and conservancy analysis of selected epitopes

### Multi-epitope vaccine design

Structure of 11 epitopes finalized in Table 1 were then derived from their respective proteins and these epitopes were then analyzed in all possible combinations using HADDOCK 2.4. HADDOCK analysis revealed that NC2-RT4 has the lowest score among all combinations (Supplementary Table S3), hence the best combination. Further NC2-RT4 was joined using a flexible linker GGGS, its structure was predicted and its combination with remaining epitopes was analyzed which revealed that NC2-RT4-RT2 is the best possible combination of three epitopes. Subsequently, these steps were repeated until the final construct of the multi-epitope vaccine (HIV-1a) is achieved (NC2-RT4-RT2-Int1-Int2-RT3-NC1-NC3-VPU1-RT1-RT5). Each possible combination selected for vaccine construct along with their respective HADDOCK score is mentioned in Table 2. Moreover, another multi-epitope vaccine construct (HIV-1b) was formulated with an adjuvant, HBD-3 with RR motif (GIINTLQKYYCRVRGGRCAVLSCLPKEEQIGKCSTRGRKCCRRKKRR), attached to N-terminus of HIV-1a multi-epitope vaccine through a rigid linker (EAAAK).

**Table 2:** Selected combinations of epitopes and their HADDOCK score

**Epitope combination**	**HADDOCK score**
NC2–-RT4	-88.3 ± 1.1
NC2-RT4–-RT2	-84.0 ± 4.2
NC2-RT4-RT2–-Int1	-67.4 ± 2.7
NC2-RT4-RT2-Int1–-Int2	-84.0 ± 3.5
NC2-RT4-RT2-Int1-Int2–-RT3	-72.6 ± 4.6
NC2-RT4-RT2-Int1-Int2-RT3–-NC1	-61.7 ± 2.2
NC2-RT4-RT2-Int1-Int2-RT3-NC1–-NC3	-52.2 ± 4.2
NC2-RT4-RT2-Int1-Int2-RT3-NC1-NC3–-VPU1	-60.9 ± 9.0
NC2-RT4-RT2-Int1-Int2-RT3-NC1-NC3-VPU1–-RT1	-58.4 ± 7.7
NC2-RT4-RT2-Int1-Int2-RT3-NC1-NC3-VPU1-RT1–-RT5	-46.4 ± 1.7

### Prediction of vaccine construct physio-chemical properties based on sequences

Physicochemical analysis of both vaccine constructs predicted that both constructs have < 50 kDa molecular weight and lower GRAVY index. Although the instability index of both vaccines is rather high, their half-lives in mammalian reticulocytes, yeast, and *E. coli* indicate that both vaccines are relatively stable *in-vitro* and *in-vivo*. Moreover, the high aliphatic index of HIV-1b vaccine qualifies it as thermostable. Physiochemical properties of both constructs are mentioned in Table 3.

**Table 3:** Physio-chemical properties of HIV-1a and HIV-1b multi-epitope vaccines

**Physicochemical properties**	**HIV-1a**	**HIV-1b**
**Number of amino acids**	139	191
**Molecular weight**	~ 14.6 kDa	~ 20.5 kDa
**Theoretical pI**	9.41	9.82
**Estimated half-life in mammalian reticulocytes (** ***in-vitro*** **)**	> 20 h	30 h
**Estimated half-life in yeast (** ***in-vivo*** **)**	> 20 h	> 20 h
**Estimated half-life in ** ***E. coli*** ** (** ***in-vivo*** **)**		> 10 h
**Instability index**	49.5	53.27
**Aliphatic index**	24.53	35.24
**GRAVY index**	-1.157	-1.064

### Antigenicity and allergenicity of HIV-1a and HIV-1b

VaxiJen v2.0 predicted 0.8778 and 0.8028 antigenicity scores for HIV-1a and HIV-1b respectively, while ANTIGENpro predicted 0.810806 and 0.746564 scores for HIV-1a and HIV-1b, respectively. Both software demonstrated that HIV-1a and HIV-1b constructs have retained their antigenic potential. Moreover, AlgPred and AllergenFP v.1.0 classified both constructs as non-allergen.

### Immune simulation

**Fig. 1:**
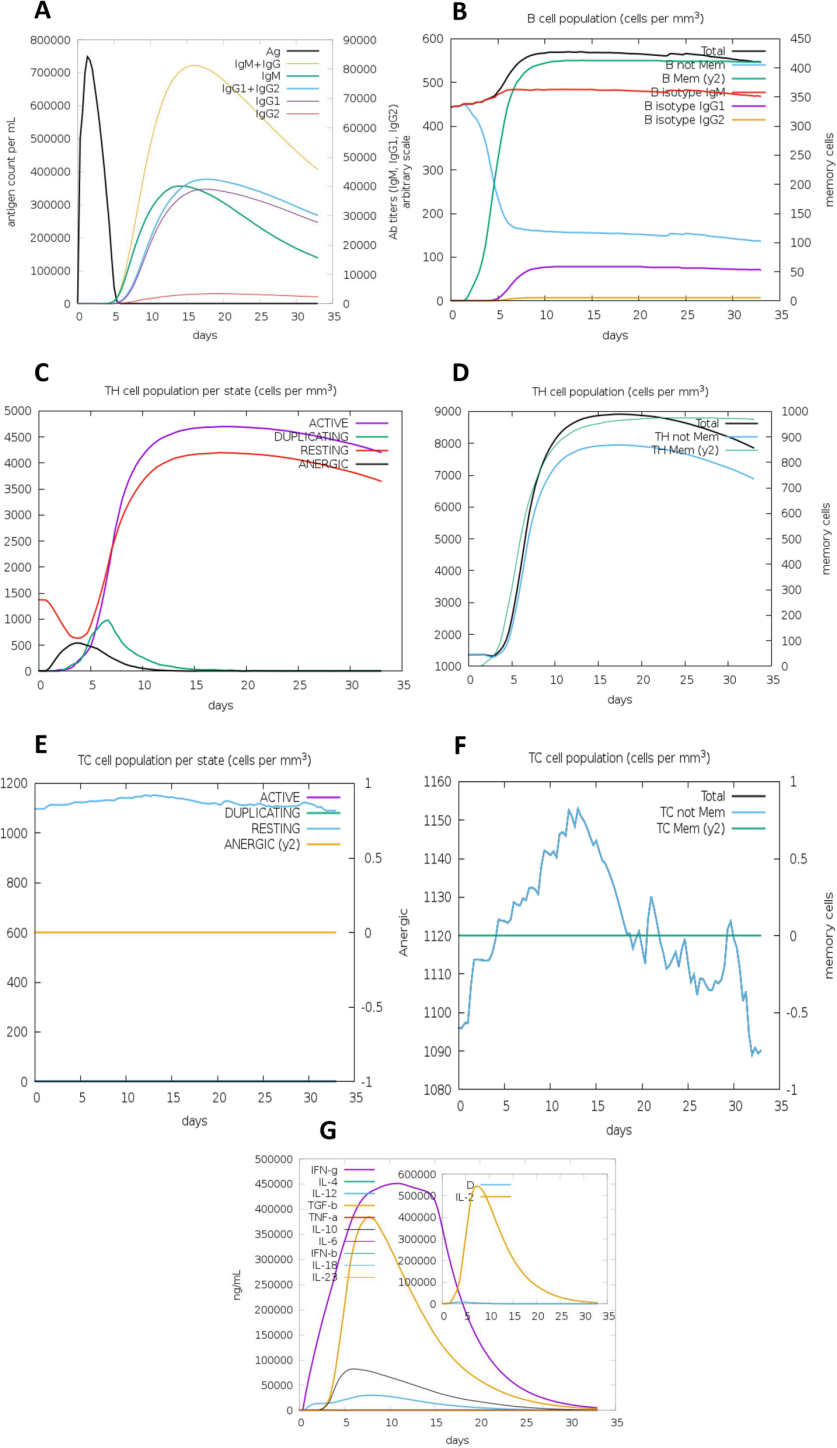
Immune simulations results of HIV-1a as obtained from C-IMMSIM. **A** Production of various types of Igs and immunocomplexes formation induced by antigen. **B** B cell population. **C** T-helper cell population per state. **D** Total TH cell population. **E** Cytotoxic T cell population per state. **F** TC cell population. **G** Elevated levels of interleukins and cytokines. Inset plot shows high production of IL-2 along with danger signal

**Fig. 2:**
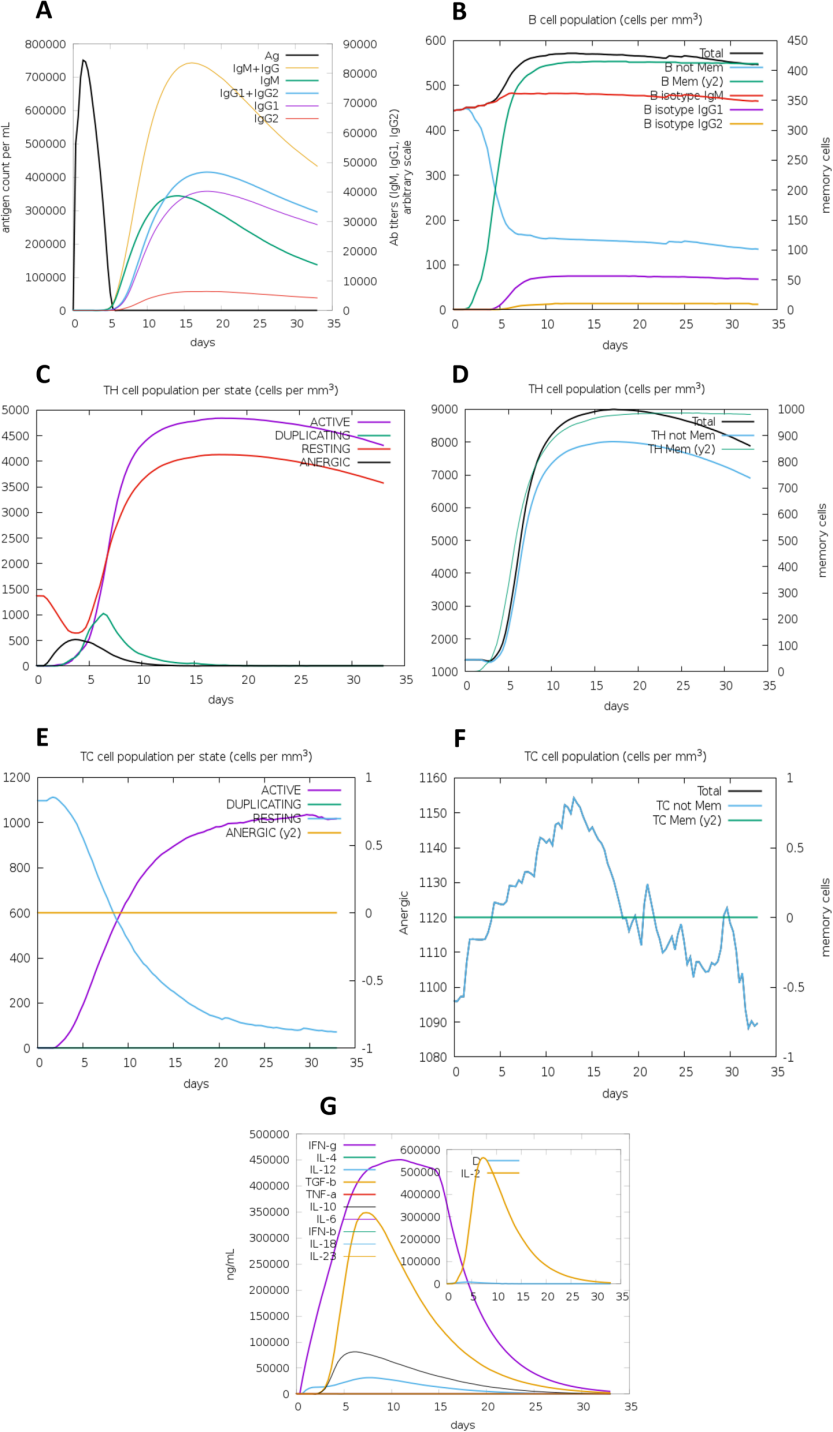
Immune simulations results of HIV-1b as obtained from C-IMMSIM. **A** Production of various types of Igs and immunocomplexes formation induced by antigen. **B** B cell population. **C** T-helper cell population per state. **D** Total TH cell population. **E** Cytotoxic T cell population per state. **F** TC cell population. **G** Elevated levels of interleukins and cytokines. Inset plot shows high production of IL-2 along with danger signal

The immunological response of HIV-1a and HIV-1b vaccine constructs was simulated using the C-IMMSIM interface. HIV-1b was predicted to have a stronger immune response as it depicts a diverse cytotoxic T-cell response. After the vaccines were injected, several immunological responses were identified, including TC (cytotoxic) concentrations, TH-cell (helper) and B-cell population levels, IgM and IgG production, and cytokine generation. HIV-1b has also shown to induce slightly more Ab titers (particularly IgG2), B isotype IgG2 cell population, active TH cell population, TH Mem (y2) cell population and IL-2 levels than HIV-1a vaccine. The results of the immunological simulation showed that both proposed vaccines elicited adequate innate and adaptive immune responses as shown in Figs. 1 and 2.

### Tertiary structure prediction, refinement, and quality analysis

Tertiary structures of HIV-1a and HIV-1b were predicted using the 3Dpro tool and these structures were further refined to obtain most Rama-favored and energy minimized structure. Statistics of before and after refinement for both structures are mentioned in Table 4.

**Table 4:** Statistics of HIV-1a and HIV-1b structures before and after refinement

**Characteristics**	**HIV-1a**		**HIV-1b**	
	**Before refinement**	**After refinement**	**Before refinement**	**After refinement**
**GALAXY energy**	17,623.80	-1874.85	29,357.35	-2694.01
**Rama-favored rotamers (%)**	86.9	95.8	84.7	97.2
**Poor rotamers (%)**	10.7	2.1	2.4	2.8
**RMSD**	0.0	2.096	0.0	1.158

GALAXY energy, Rama-favored and poor rotamers, and Root Mean Square Deviation (RMSD) of HIV-1a and HIV-1b structures before and after refinement

**Fig. 3:**
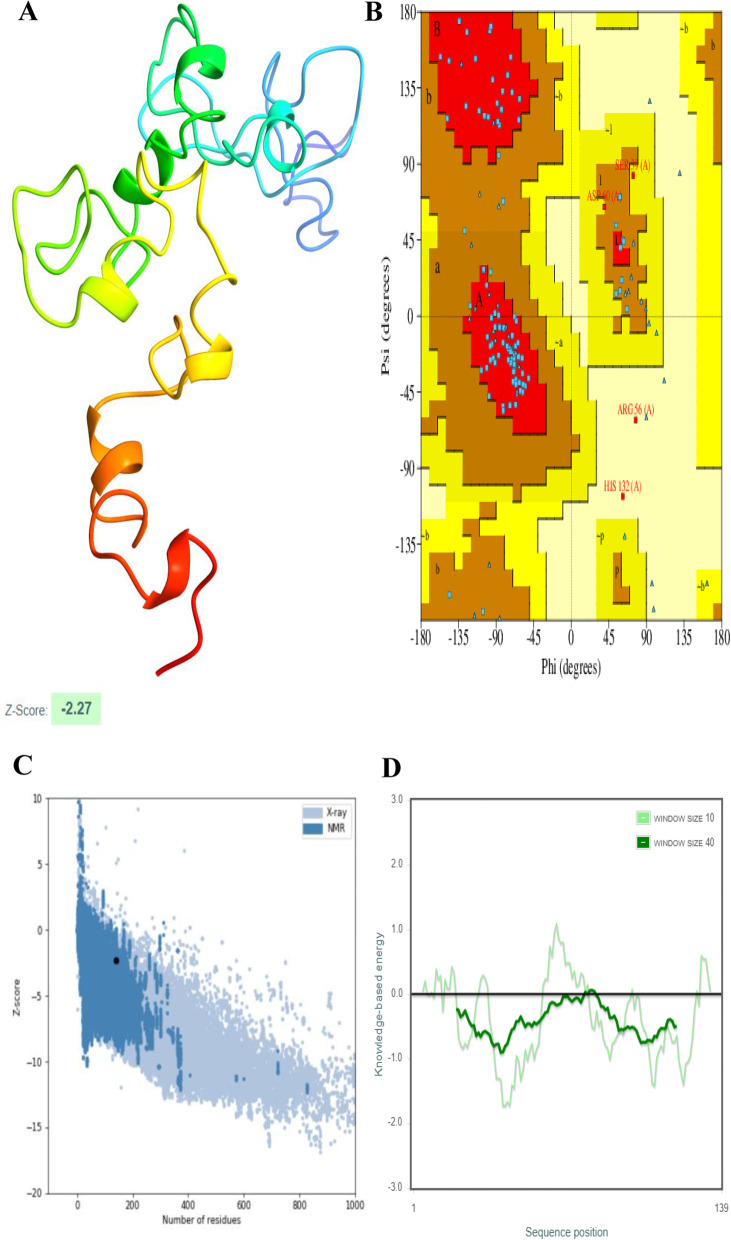
Predicted tertiary structure, Ramachandran plot and model quality analysis of HIV-1a. **A** Refined structure. **B** Ramachandran plot of the refined structure. **C** Overall model quality showed that protein structure lies in the allowed region. **D** Local model quality through a window size of 40 amino acids also showed that most part of the protein falls under the knowledge-based energy

**Fig. 4:**
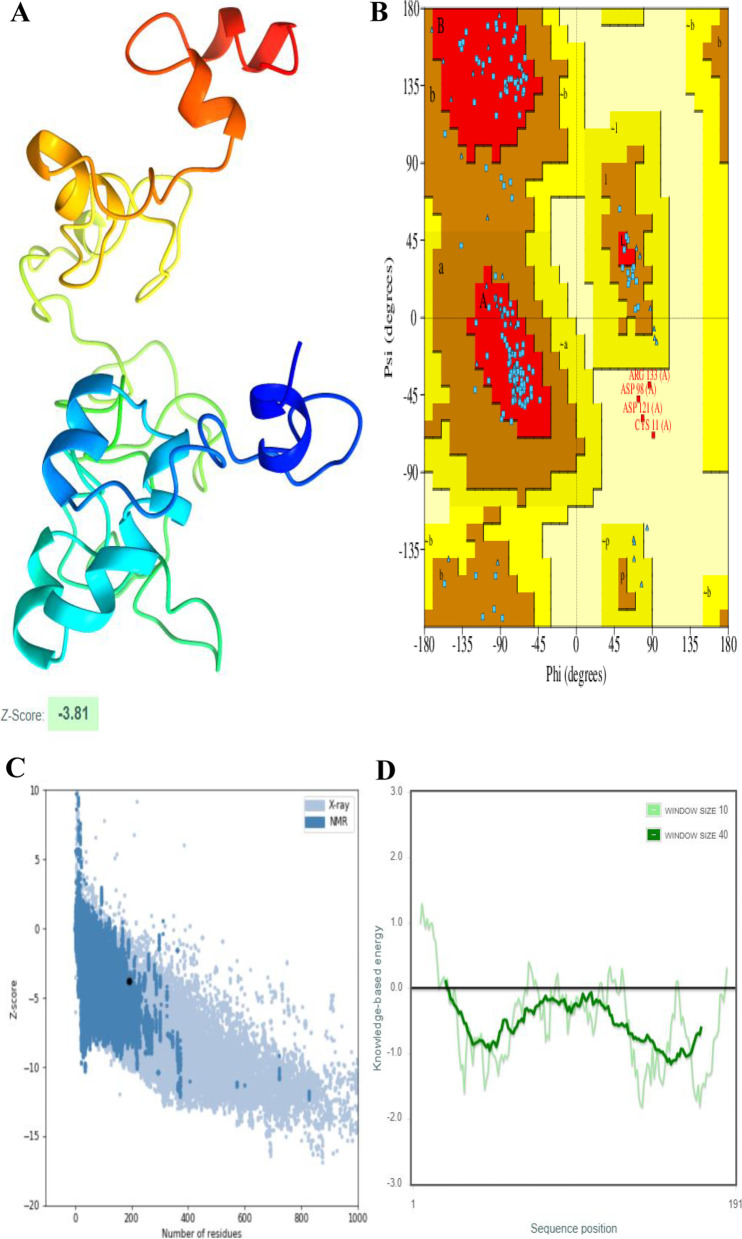
Predicted tertiary structure, Ramachandran plot and model quality analysis of HIV-1b. **A** Refined structure. **B** Ramachandran plot of the refined structure. **C** Overall model quality showed that protein structure lies in the allowed region. **D** Local model quality through a window size of 40 amino acids also showed that most part of the protein falls under the knowledge-based energy

Moreover, overall and local model quality (window size of 40 amino acids) showed that both HIV-1a and HIV-1b refined structures lie inside the range of acceptable z-score i.e., -2.27 and -3.81 respectively and knowledge-based energy. Refined structures, Ramachandran plots, along with Z-score and knowledge base energy graphs for both vaccines are shown in Figs. 3 and 4.

### MD simulation

**Fig. 5:**
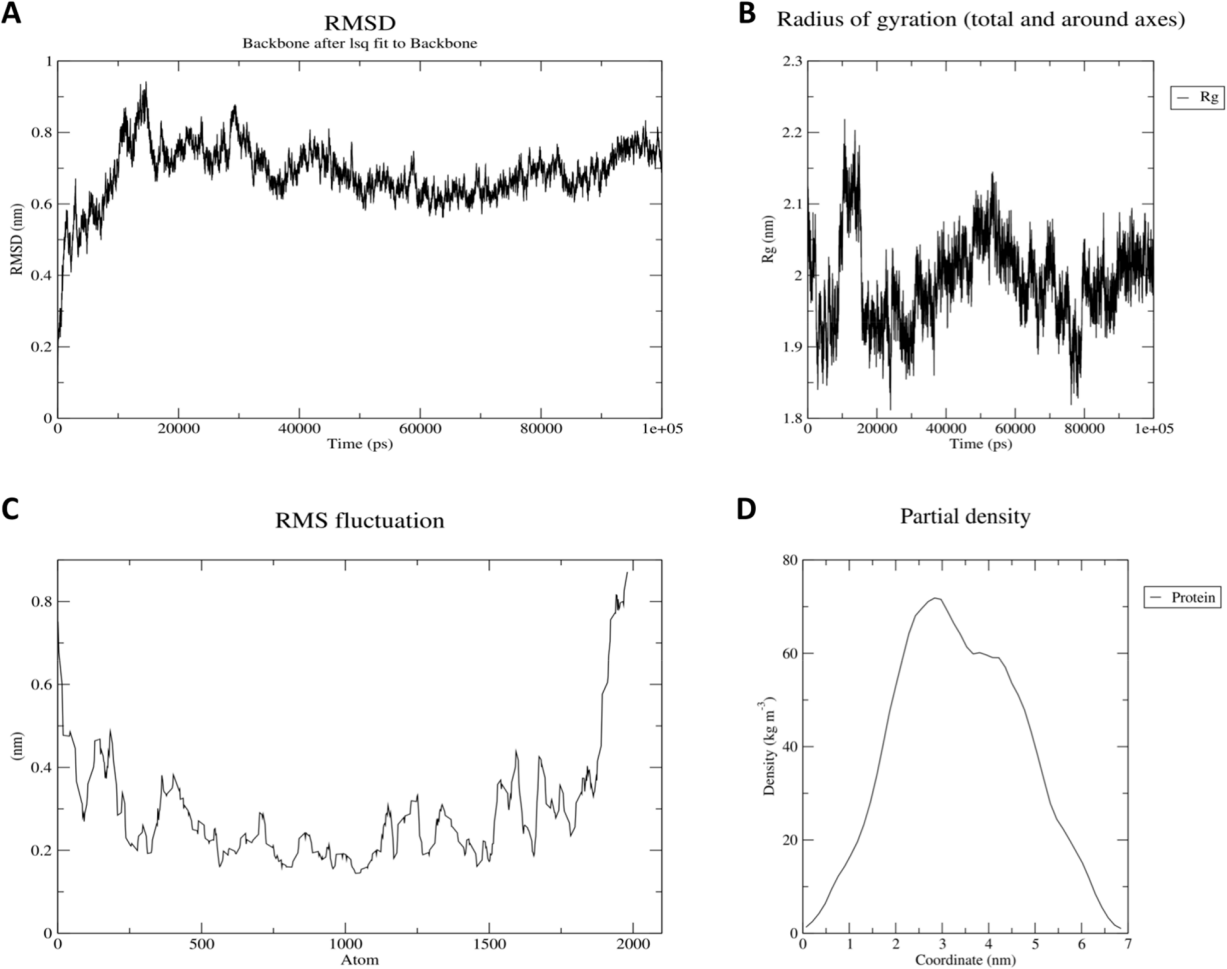
RMSD, Rg, RMSF, and partial density of HIV-1a. **A** The RMSD graph has the peak around 17,000 and 29,000 ps of almost 0.9 nm and 0.85 nm respectively, which is still much less and still in the required zone of 1.0 nm. The overall graph shows the stability, with an average RMSD value of almost 0.65 nm. **B** Radius of gyration shows a little peak at the start but has overall stable Rg value and shows that the protein is stably folded. **C** RMSF doesn’t show much deviation as per atom, which determines that the structure is stable. **D** Protein density graph is smooth and stable as per the graph for HIV-1a

**Fig. 6:**
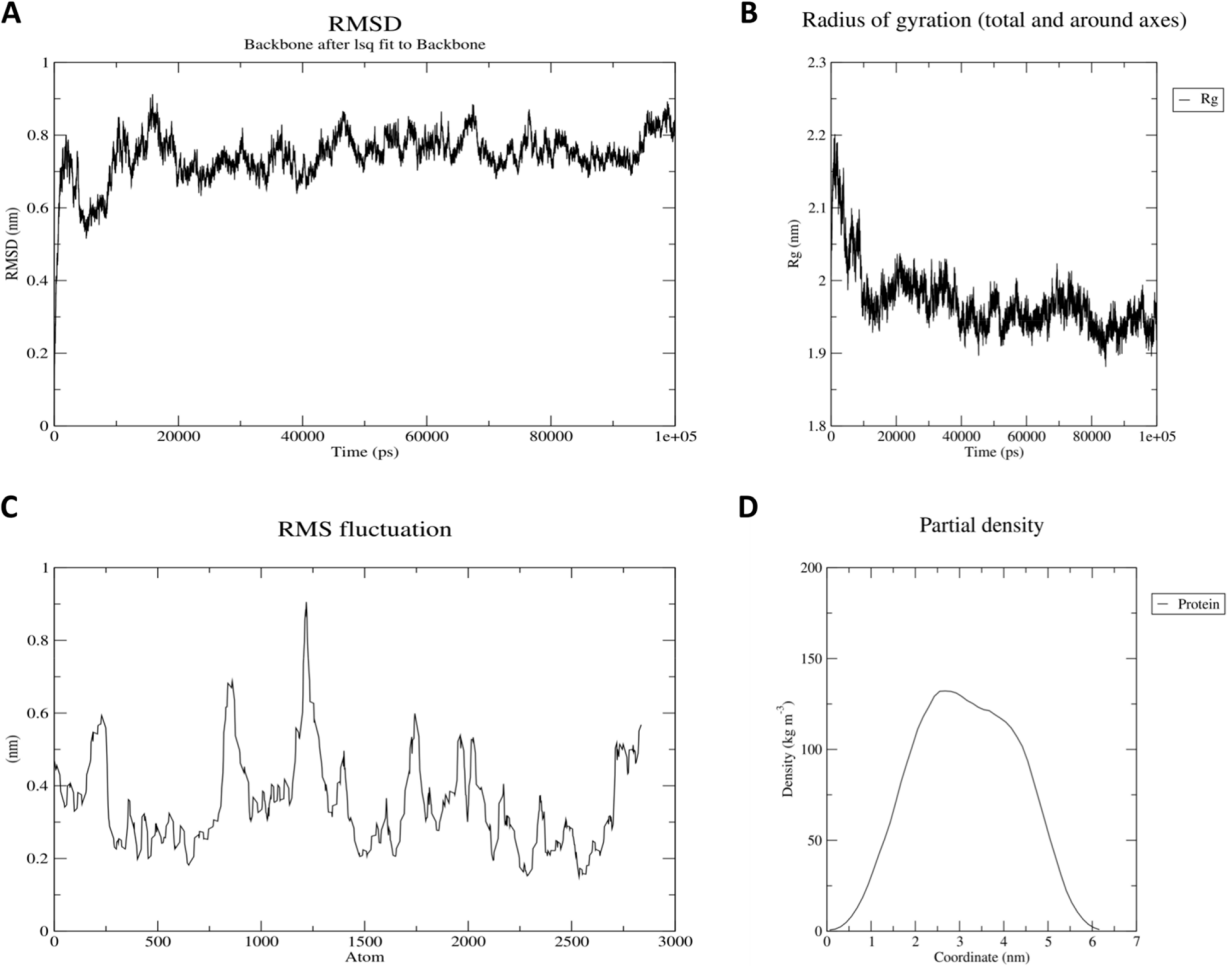
RMSD, Rg, RMSF, and partial density of HIV-1b. **A** The RMSD graph is almost stable around 0.8 nm and has no recognizable peak. Although the value is a bit higher as compared to HIV1a but is still less than 1.0 nm. The overall graph shows the stability, with an average RMSD value of almost 0.8 nm. **B** Radius of gyration shows a little peak at the start but has overall stable Rg value and shows that the protein is stably folded. **C** RMSF shows some peaks, suggesting that the protein structure has flexibility in some areas but still is in quite acceptable range, which determines that the structure is almost stable. **D** Protein density graph is smooth and stable as per the graph for HIV-1b

For HIV-1a and HIV-1b vaccine constructs, energy minimization, pressure, temperature, density, radius of gyration (Rg), and potential energy calculation were also done or calculated. During the MD simulation, both constructs overall structure remained stable, as evidenced by the radius of the gyration graph (Figs. 5 and 6). For RMSD and RMSF, a study of the trajectory generated after a 100 ns simulation yielded a radius of gyration. The RMSD levels reach up to 0.95 nm for HIV-1a and 0.9 nm for HIV-1b and showed stability with an average RMSD value of 0.65 nm for HIV-1a and 0.8 nm for HIV-1b during the simulation time, indicating that both structures are relatively stable (Figs. 5 and 6). The overall structure of the protein remained stable during the MD simulation, according to RMSF (Figs. 5 and 6).

### Secondary structure prediction

**Fig. 7:**
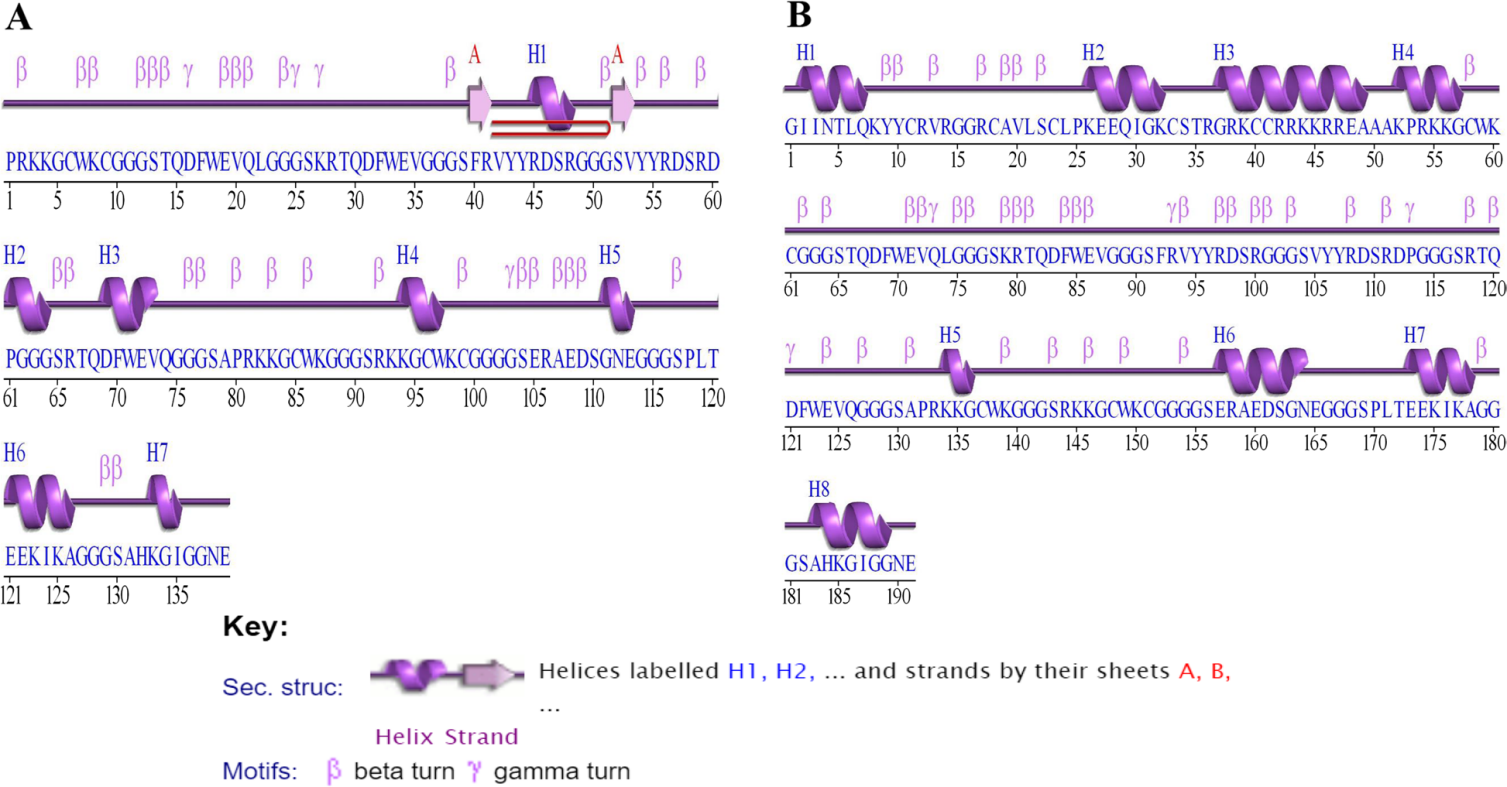
Secondary structure of (**A**) HIV-1a, and (**B**) HIV-1b

Secondary structure of HIV-1a and HIV-1b predicted through PDBsum demonstrated the presence of 1 β-sheet, 2 β-strands, 1 β-hairpin, 7 helices, 32 β-turns, and 4 ɣ-turns in HIV-1a, while 8 helices, 39 β-turns, and 4 ɣ-turns in HIV-1b. Secondary structures of both vaccines are illustrated in Fig. 7.

### TLR-3 docking

**Fig. 8:**
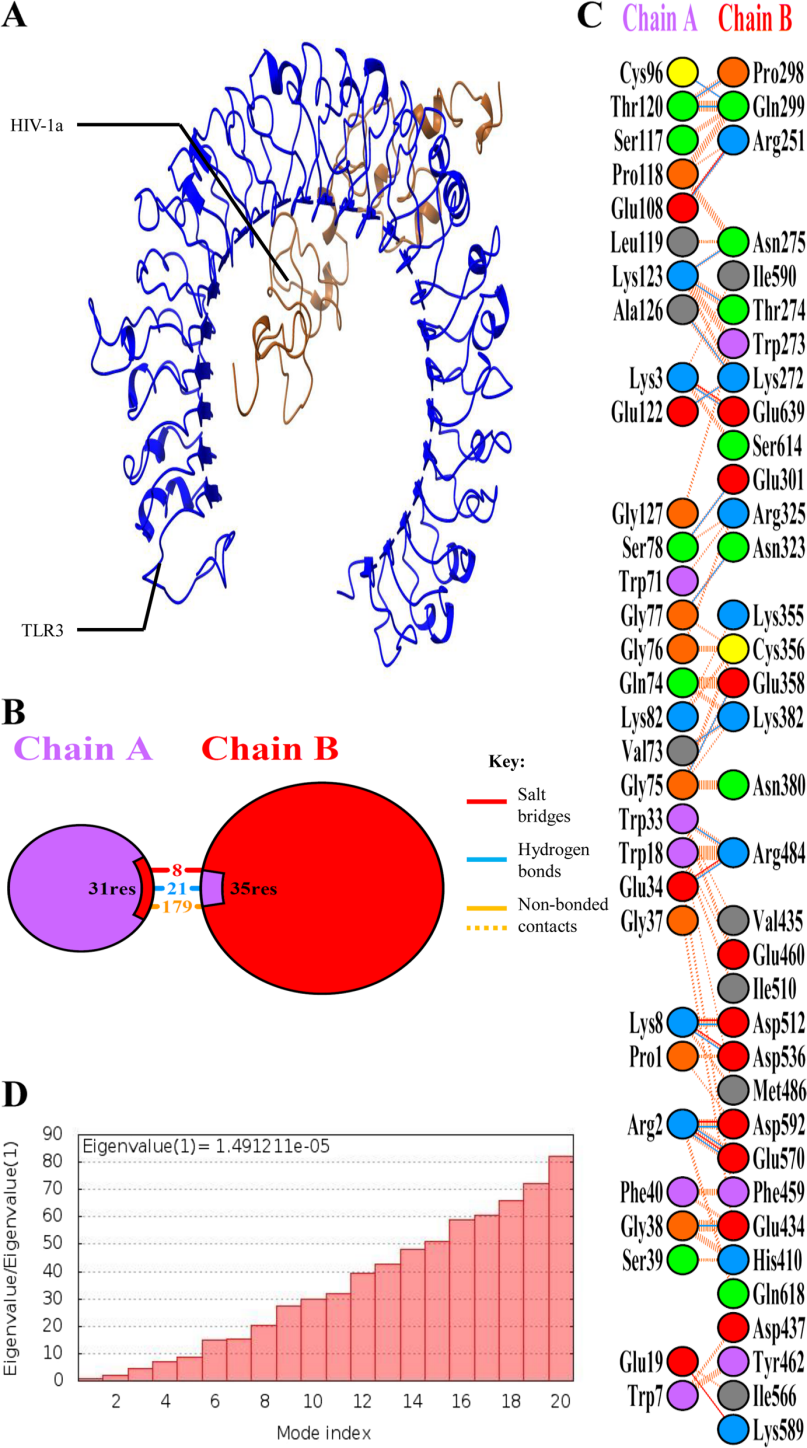
HIV-1a-TLR-3 complex structure and interaction details. **A** HIV-1a-TLR-3 complex refined structure. **B** Summary of HIV-1a-TLR-3 interactions. **C** Interacting residues of HIV-1a and TLR-3. HIV-1a forms 8 salt bridges, 21 hydrogen bonds, and 179 non-bonded contacts with TLR-3. **D** Eigen value of HIV-1a-TLR-3 complex

**Fig. 9:**
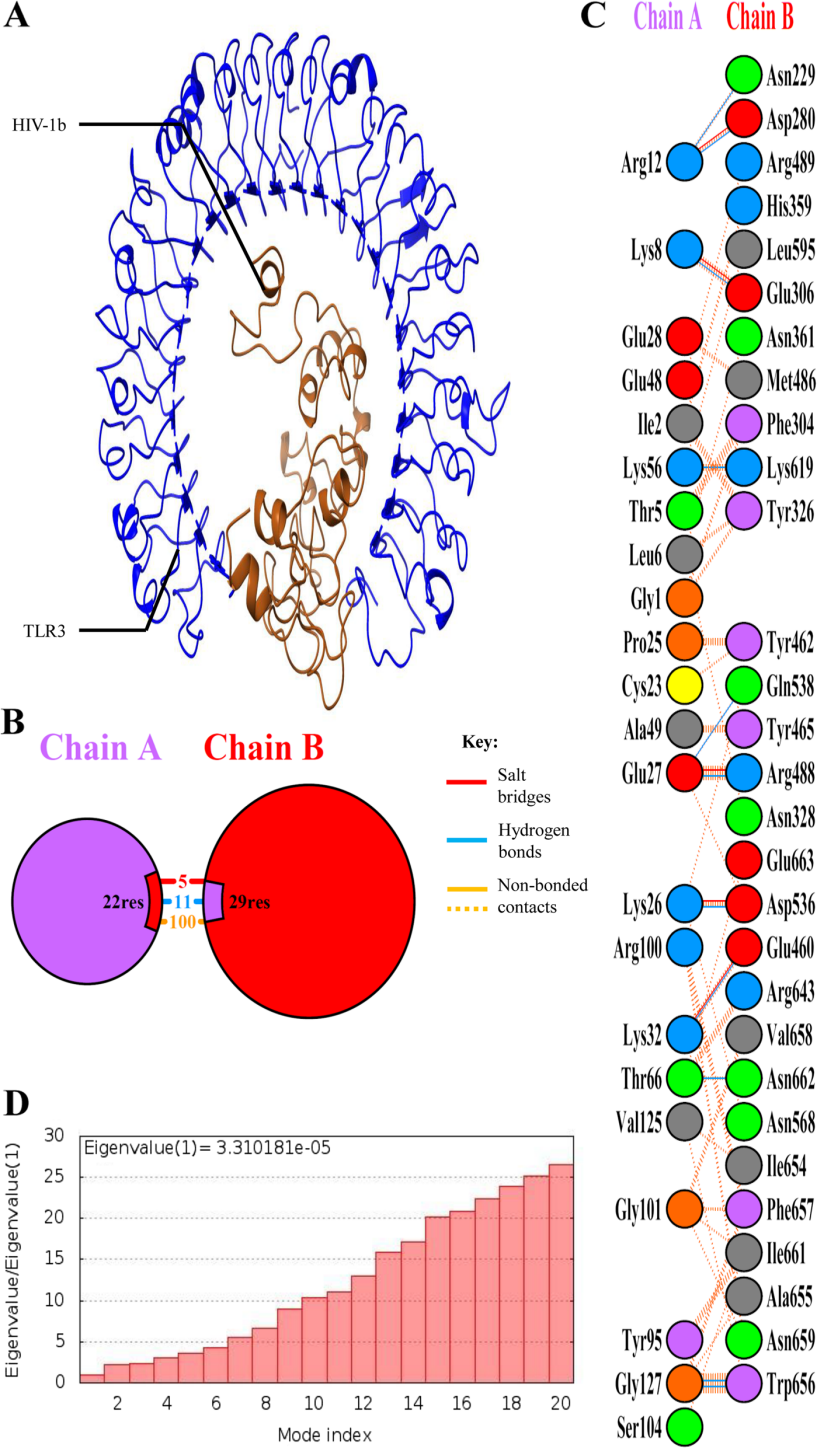
HIV-1b-TLR-3 complex structure and interaction details. **A** HIV-1b-TLR-3 complex refined structure. **B** Summary of HIV-1b-TLR-3 interactions. **C** Interacting residues of HIV-1b and TLR-3. HIV-1b forms 5 salt bridges, 11 hydrogen bonds, and 100 non-bonded contacts with TLR-3. **D** Eigen value of HIV-1b-TLR-3 complex

TLR-3 docking with HIV-1a and HIV-1b was performed using HADDOCK 2.4. Both vaccines returned with a negative HADDOCK score which suggests strong binding with TLR-3. After HADDOCK refinement, these scores were significantly improved. Detailed parameters of HIV-1a and HIV-1b docking with TLR-3 before and after refinement are mentioned in Table 5. NMA of these complexes were performed to determine the eigen value (energy required to deform the structure). Refined structure of HIV-1a-TLR-3 and HIV-1b-TLR-3 complexes along with their residual interaction details, summary of interactions, and eigen value are illustrated in Figs. 8 and 9. Eigen value or deformation energy of HIV-1b-TLR-3 is higher than HIV-1a-TLR-3 which represents HIV-1b-TLR-3 stronger docking.

**Table 5:** Molecular docking parameters of HIV-1a-TLR-3 and HIV-1b-TLR-3 complexes

**Parameter**	**HIV-1a**		**HIV-1b**	
	**Before refinement**	**After refinement**	**Before refinement**	**After refinement**
**HADDOCK score**	-82.3 ± 26.8	-241.6 ± 7.2	-70.4 ± 26.8	-179.5 ± 2.7
**Cluster size**	6	20	5	20
**RMSD from the overall lowest energy structure**	1.0 ± 0.7	0.6 ± 0.3	0.9 ± 0.5	0.6 ± 0.3
**Van der Waals energy**	-57.9 ± 15.8	80.2 ± 7.7	-86.7 ± 3.5	-95.2 ± 3.7
**Electrostatic energy**	-643.0 ± 93.1	-788.5 ± 41.5	-358.3 ± 76.1	-320.2 ± 23.7
**Desolvation energy**	-1.8 ± 3.8	3.6 ± 1.4	-17.9 ± 1.4	-20.3 ± 5.3
**Restraints violation energy**	1059.9 ± 181.2	0.0 ± 0.0	1058.0 ± 232.6	0.0 ± 0.0
**Buried surface area**	2812.3 ± 177.3	3119.2 ± 68.0	3320.6 ± 184.1	3329.5 ± 71.9
**Z-score**	-1.4	0.0	-1.1	0.0

### Codon optimization and *in-silico* cloning

**Fig. 10:**
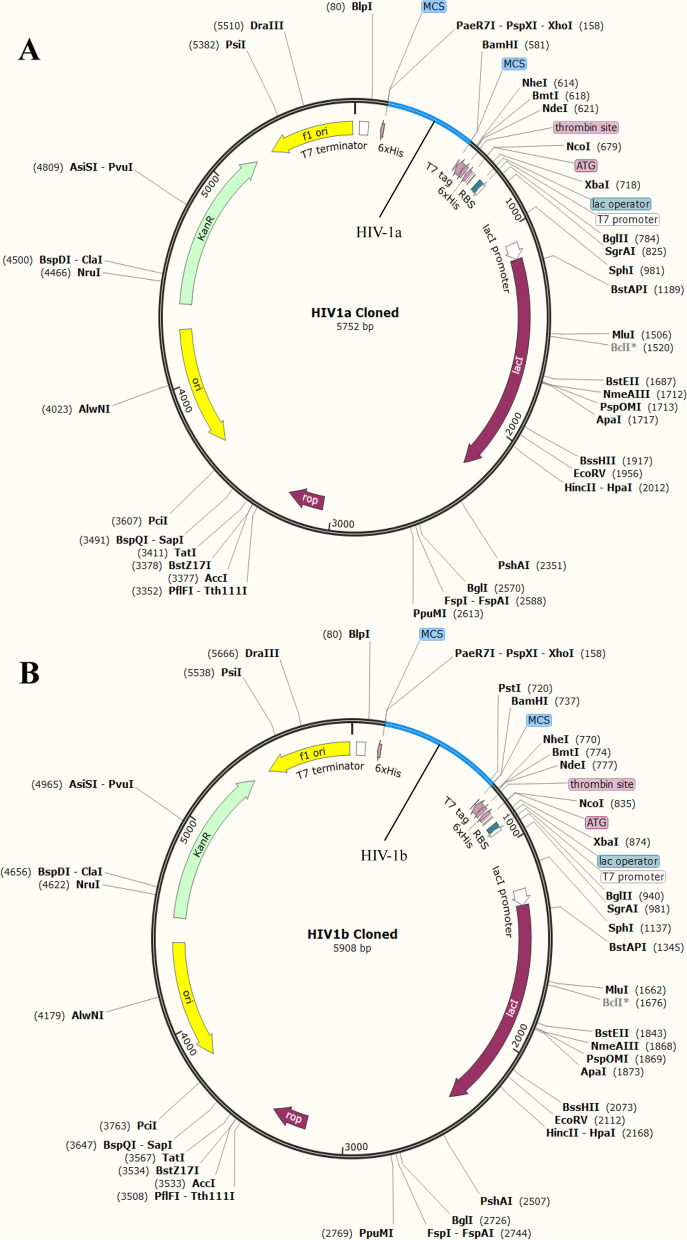
Cloned (**A**) HIV-1a, and (**B**) HIV-1b in pET28(a) + plasmid. CAI value of both multi-epitope vaccines suggest high expression levels in *E. Coli* system

GC content of both vaccine HIV-1a and HIV-1b were under the optimum limits i.e., 52.04 and 51.48, respectively, after codon optimization. The Codon Adaptation Index (CAI) value was also predicted to be 1.0 for both vaccines which estimates their high expression in *E. Coli* K12 strain. After addition of restriction sites at N- and C- termini of both vaccines, they were cloned in pET28(a) + plasmid as demonstrated in Fig. 10.

## Discussion

Despite advancements in tackling AIDS through antiretroviral treatments, there is a dire need to formulate an effective vaccine against HIV-1 to control this deadly global epidemic [3]. However, most of the devised vaccines have failed so far partly due to their less efficacy against this rapidly mutating organism as previously designed vaccines tend to target a few genotypes of HIV [13]. This ineffectiveness of vaccines to target HIV-1 can also be attributed to their inability to induce both cellular and helper T-cell responses as suggested in trials of an in-silico designed multi-epitope vaccine EP HIV-1090 [10], or their incompetence to induce broadly neutralizing antibodies as shown by BALB/c mice trials on three multi-epitope vaccines [9], appropriate cytokines, or inability to activate desired innate immune response. The methodology adopted in this study is similar to prior research used to design multiepitope vaccines against microbes such as HIV, Orthohantavirus, and *T. whipplei* [13–15]. However, other multiepitope vaccines are primarily limited by their ability to elicit an immune response against a few genotypes only. Particularly in the case of HIV, a vaccine designed by Pandey et al. only confers immunity against subtypes B and C of the group M virus. The current research is distinctive in many aspects since it targeted all the genotypes of HIV, has included both humoral and cellular immune responses, and used multiple tools for the evaluation of vaccines’ stability, efficacy, and toxicity.

The current study aimed to develop a multi-epitope vaccine against HIV-1 through an immuno-informatics approach similar to other studies [16, 17]. Multi epitope-based vaccines have the ability to induce both cellular and helper T-cell responses. Moreover, methodology followed in these studies helped to propose a multi-epitope vaccine that can efficiently induce broadly neutralizing antibodies and an appropriate cytokine such as IFN-ɣ [8] along with induction of appropriate innate response through docking with TLR-3 [13] and inclusion of an adjuvant (HBD-3 with RR motif at C-terminal) [12] at N-terminal of the vaccine.

Multi-epitope vaccines designed through *in-silico* approaches have gained tremendous importance following the promising results of various studies. An *in-silico* study was used to design a multi-epitope vaccine against Onchocerciasis caused by microflaria [18]. Another multi-epitope vaccine against MERS was designed using an immuno-informatics approach [19]. A multi-epitope vaccine against brucellosis demonstrated promising T-cell response [20]. Moreover, pre-clinical trials of multi-epitope vaccine against Epstein-Barr virus in mice models have also shown promising results [21]. Similarly, studies on malaria and cancer [22] validate the methodology used in our study as multi-epitope vaccines have shown to be far more progressive than monovalent vaccines due to their ability to induce both cellular and humoral immune responses [23]. These approaches save a lot of financial and time investments, that pose a considerable burden for vaccine research.

In the current study, epitopes were predicted using the entire HIV-1 proteome (VIF, VPR, TAT, REV, VPU, NEF, GAG, POL, and ENV). These 9 constituents proteins and polyproteins play crucial roles in the HIV-1 life cycle and are necessary for the virus to reproduce and cause disease; hence, targeting these proteins can be an effective measure for preventing or treating HIV-1 infection [24–26]. Several of these polyproteins, including GAG, POL, and ENV, are known to be immunogenic, or capable of eliciting a robust immune response [27]. In addition, HIV-1 is known for its high genetic diversity, and the protein sequences of various strains of the virus can differ significantly. Thus, targeting numerous proteins can aid in the development of vaccine that is effective against a wide variety of HIV-1 strains [28]. 11 epitopes were selected to generate two multi-epitope vaccines, one without adjuvant (HIV-1a) and one with adjuvant (HIV-1b). First, B and T-cell epitopes were predicted as this is the fundamental step in vaccine designing. Then, T-cell epitopes overlapping with B-cell epitopes and inducing IFN-ɣ were filtered [29]. Thus, the selected epitopes have the ability to induce cellular T-cell, helper T-cell, B-cell, and IFN-ɣ responses. Subunit vaccines comprising of cellular and helper T-cell epitopes are under clinical trials [30]. Safety profile of selected epitopes was also evaluated through toxicity and allergenicity analysis.

Selected epitopes were then docked in every possible combination to determine the final vaccine construct with lowest HADDOCK score. The HADDOCK scoring method evaluates the binding affinity of the complex based on the energy of the interaction between the molecules. The energy is calculated by considering several factors, including electrostatics, van der Waals forces, Desolvation energy, and the change in the surface area of the molecules upon binding [31]. Hence, the HADDOCK scoring method ranks the docking pose based on their energy score, with lower energy scores indicating stronger binding affinity [32]. HADDOCK scores can help identify the most stable and energetically favorable epitope combinations. Linkers are an important component of multi-epitope vaccines as they help to maintain the individuality of each epitope after their introduction to the human body [33]. GGGS was used as a linker to join epitopes as suggested by Yang et al. [9]. A variant of antimicrobial peptide (β-defensin), HBD-3, with RR motif at C-terminus was added to the N-terminus in HIV-1b vaccine. Previously, defensin peptides have shown a potential role as a mucosal adjuvant in mouse models. When combined with a vaccine prototype as a synthetic adjuvant, the cellular immunogenicity of HIV peptide with defensin formulations revealed a substantially greater (p 0.001) proliferative response than HIV peptides administered alone in mouse models [34]. HBD-3 helps in antigen specific antibody production through Th1 and Th2 dependent pathways [35]. Moreover, enhancing positive charge of the adjuvant has shown to enhance antibody production against HIV-1 [12], so the RR motif was attached to C-terminus of HBD-3. EAAAK linker was used to join adjuvant to multi-epitope vaccine as it reduces the chance of interaction among functional domains [36].

TLR-3 docking showed that both vaccines have strong binding capacities. However, NMA showed that HIV-1b-TLR-3 has higher deformation energy than the HIV-1a-TLR-3 complex. TLR-3 recognizes double stranded RNA, and it has shown to inhibit HIV-1 infection through the induction of type 1 interferon and inflammatory cytokine/chemokine [37]. Activation of TLR-3 can lead to a TRIF dependent signaling cascade, which can induce the transcription of inflammatory genes [38]. Its activation in dendritic cells (DCs) causes them to develop into powerful immunostimulatory cells that can effectively cross-prime T lymphocytes [39]. In the case of HIV-1 infection, activation of dendritic cells is dependent on TLR-3 activation [40]. HBD-3 is specifically chemotactic for cells expressing CCR6, which is primarily expressed by immature dendritic cells [41]. Thus, the chemotactic activity of HBD-3 on immature dendritic cells and their TLR-3 dependent activation along with cross-priming of T-lymphocytes can lead to better induction of an innate immune response and hence a better adaptive immune response. While other TLRs like TLR-4 can lead to the induction of IL-6 which can reactivate the virus from its latency stage [42]. Similarly, TLR-10 is also known to enhance HIV-1 infection [43]. Physicochemical properties of both vaccines suggested that both vaccines are relatively stable considering their half-lives *in-vitro* and *in-vivo*, although instability index has shown that both vaccines maybe relatively unstable, aliphatic index has shown that HIV-1b is relatively more thermostable than HIV-1a. Moreover, Structural parameters like GALAXY energy, Rama-favored rotamers, and Z-score also suggested that although both vaccines are fairly stable, HIV-1b is more stable than HIV-1a. MD simulations has also suggested that both vaccines are fairly stable. Immune simulations suggested that both vaccines elicited sufficient immune response, however, HIV-1b has elicited more promising results. The CAI values of both vaccine constructs also showed high compatibility and expression in their host system. Despite the numerous promising results of this study, experimental validation is required to verify the results.

## Conclusion

In conclusion, immuno-informatics approaches were used to design two multi-epitope vaccines, HIV-1a (with adjuvant) and HIV-1b (without adjuvant). Both vaccines are predicted to be antigenic, non-allergenic, fairly stable, non-human homologue, and can induce cellular T-cell, helper T-cell, B-cell, and IFN-ɣ responses. However, HIV-1b has demonstrated better docking results and stability than HIV-1a. Thus, the proposed vaccine constructs merit further in vitro as well as in vivo testing.

## Methods

**Fig. 11:**
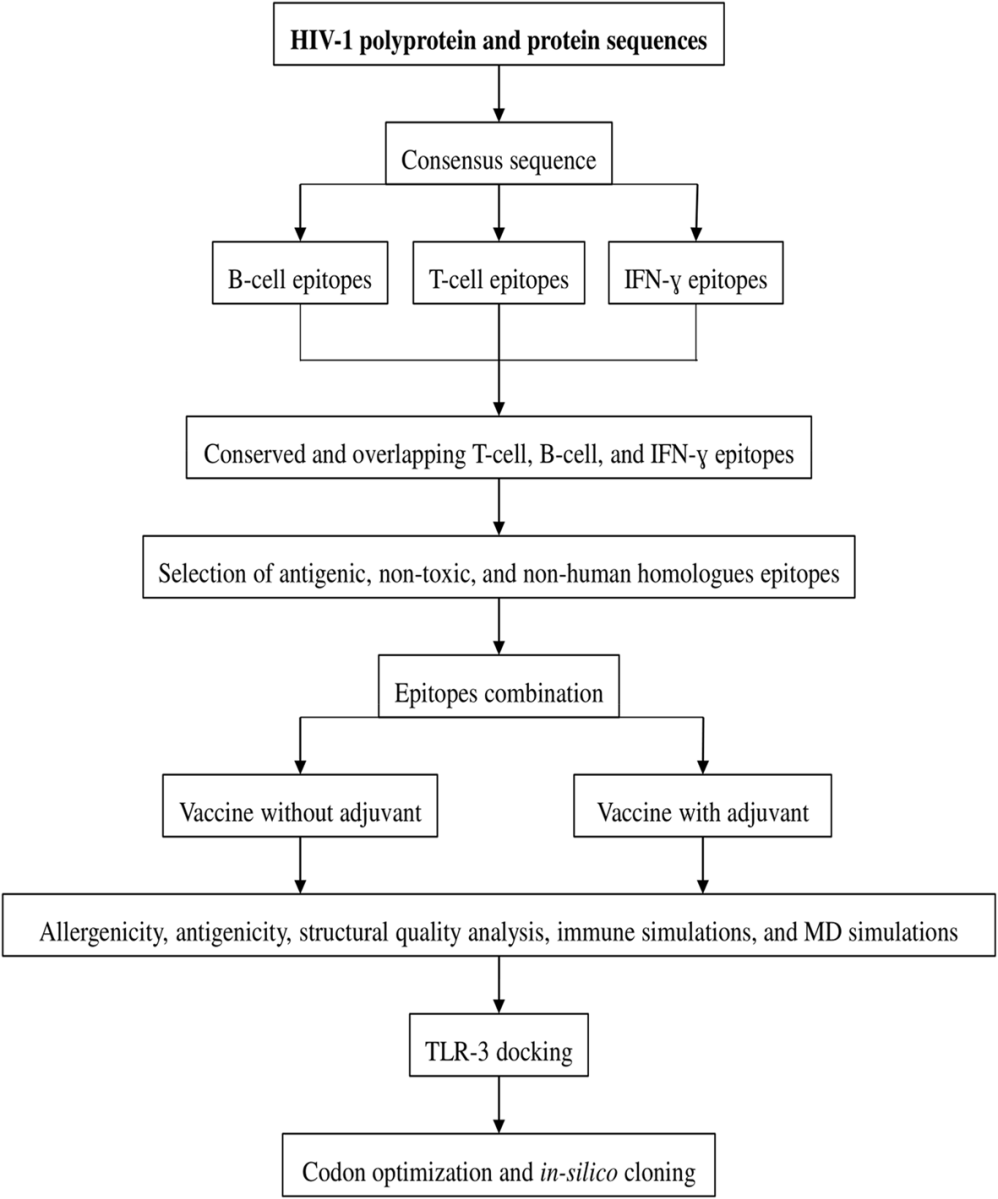
Flowchart of the methodology followed in this study

The methodology followed in this study comprises the following parts (1) T-cell, B-cell, and IFN-ɣ epitopes prediction from the viral proteins; (2) screening of conserved T-cell epitopes overlapping with B-cell and IFN-ɣ epitopes; (3) designing two vaccine constructs (with and without adjuvant) through epitope-epitope interaction analysis and structural modelling using appropriate linkers; (4) characteristic analysis of protein through molecular dynamics evaluation; (5) molecular docking with TLR-3; and (6) Codon optimization and *in-silico* cloning. Figure 11 summarizes the methodology adapted in this study in the form of a flowchart.

### Sequence retrieval and consensus sequence generation

All HIV-1 polyprotein and protein sequences were retrieved from the Los Alamos National Lab (LANL) HIV sequence database http://www.hiv.lanl.gov/ on 4^th^ December, 2020 using only one sequence/patient filter, accession numbers are listed in Supplementary Table S4. Downloaded sequences were aligned using the tree based progressive method of MAFFT version 7 https://mafft.cbrc.jp/alignment/server/index.html [44], which is a multiple alignment tool for nucleotide or amino acid sequences, with normal mode memory usage and other default values. The consensus sequences of these polyproteins and proteins were then generated using Qiagen CLC Genomics Workbench 20.0.4 [45].

### T- and B-cell epitope mapping

Both cellular and helper T-cell epitopes were mapped using the HLAPred server http://crdd.osdd.net/raghava/hlapred/index.html, which is designed for the identification and prediction of CD8 + and CD4 + T-cell epitopes by predicting binders, identification of experimental binders, and by default values. HLA-A3, HLA-A11, HLA-B27, HLA-B*2702, HLA-B*2705, HLA-B*3801, HLA-B*51, HLA-B*5801, HLA-DRB1*1301, HLA-DRB1*1302, HLA-DRB1*1502 alleles were prioritized for T-cell epitope mapping, as they have shown to be effective in controlling HIV-1 infection [46–53]. Predicted T-cell epitopes were analyzed for their conservancy using Unipro Ugene [54] and only 80% conserved epitopes were selected.

B-cell epitopes were mapped using BepiPred-2.0: Sequential B-cell epitope predictor tool of the IEDB Analysis Resource http://tools.iedb.org/bcell/ [55]. This tool uses the Random Forest algorithm which is trained on crystal structure-determined epitopes and non-epitope amino acids; afterward, it performs a sequential prediction smoothing for B-cell epitope prediction. After B-cell epitopes prediction, overlapping conserved T- and B-cell epitopes were selected for further analysis.

### Interferon-gamma epitope mapping

Overlapping T- and B-cell epitopes were predicted for their ability to induce IFN-ɣ using the IFNepitope server, a server for predicting and designing epitopes with the ability to induce IFN-ɣ http://crdd.osdd.net/raghava/ifnepitope/contact.php.

### Antigenicity

Selected epitopes were filtered for their antigenic capabilities using VaxiJen v2.0 (online available at http://www.ddg-pharmfac.net/vaxijen/VaxiJen/VaxiJen.html) [56]. This online server relies on the physiochemical properties of proteins instead of length or alignment for antigenicity prediction. Virus was selected as the target organism and a threshold of 0.5 was used to predict antigens.

### Non-human homologous epitopes selection

An ideal vaccine candidate should not contain human homologous epitopes to avoid the chances of autoimmunity. BLASTp (standard protein BLAST) https://blast.ncbi.nlm.nih.gov/Blast.cgi?PAGE=Proteins [57] was used to screen non-human homologous epitopes and only those epitopes were selected that had E-score values greater than 1.0.

### Toxicity and population coverage analysis

Filtered epitopes were checked for their ability to induce toxicity using ToxinPred (available at http://crdd.osdd.net/raghava/toxinpred/). Toxicity of epitopes was analyzed using SVM (Swiss-Prot) + motif-based method of ToxinPred, where SVM threshold value was set to 0. Moreover, only those epitopes were selected that were present in the mature proteins of HIV-1. Population coverage analysis was then also performed on the selected epitopes using IEDB resource server [58].

### Construction of multi-epitope vaccine

Finalized epitopes were checked for their compatibility among themselves to determine the order of these epitopes in the final construct. For this purpose, HIV-1 mature proteins (from which epitopes were derived) nucleocapsid (PDB ID 5I1R) [59], reverse transcriptase (PDB ID 1HMV) [60], integrase (PDB ID 6U8Q) [61], and VPU (PDB ID 1VPU) [62] were downloaded from the RCSB PDB. Further methodology for the construction of a multi-epitope vaccine can be categorized into three steps:

1. Epitopes and epitope combinations’ structure prediction.
2. Determining active and passive residues of epitopes and epitope combinations.
3. Docking different epitope combinations.

Epitopes’ structures were clipped from their respective proteins using ChimeraX v1.1 [63]. CPORT https://alcazar.science.uu.nl/services/CPORT/ [64] was then used to determine the active and passive residues of these epitopes. Afterwards, epitopes were docked among each other to determine the best possible combination using the HADDOCK 2.4 GURU level interface with default values https://bianca.science.uu.nl/haddock2.4/ [65].

The best possible combination of epitopes was then joined using an appropriate flexible linker such as GGGS as devised by Yang et al. [9]. Following this, the structure of this combination of epitopes with a suitable linker was generated using Phyre2 http://www.sbg.bio.ic.ac.uk/~phyre2/html/page.cgi?id=index [66], and then its active and passive residues were determined, and it was docked with remaining epitopes to determine best possible combination of three epitopes. Finally, these three steps were then repeated several times until the final construct of multi-epitope vaccine (HIV-1a) was generated.

Another multi-epitope vaccine construct (HIV-1b) was also generated by linking an adjuvant devised by Mohan et al. [12], human β-defensin-3 (HBD-3), a soluble peptide with an RR motif at the C-terminus, at the N-terminus of HIV-1a with a suitable rigid linker, EAAAK [17].

### Prediction of vaccine construct physio-chemical properties based on sequences

ProtParam tool https://web.expasy.org/protparam/ was used to analyze various physicochemical properties like number of amino acids, molecular weight, theoretical isoelectric point (pI), estimated half-life (*in-vitro*, and *in-vivo*) using N-end rule, aliphatic index, and Grand Average of Hydropathy (GRAVY) index of both vaccine constructs [67].

### Allergenicity of multi-epitope vaccine

AllergenFP v.1.0 https://ddg-pharmfac.net/AllergenFP/ [68] and AlgPred https://webs.iiitd.edu.in/raghava/algpred/submission.html [69] were used for evaluation of the allergenicity of both constructs (HIV-1a and HIV-1b). All 6 prediction approaches of Algpred were used to evaluate the allergenicity of both constructs i.e., mapping of IgE epitopes and PID, MEME/MAST motif, SVM module based on amino acid composition, SVM module based on dipeptide composition, BLAST search on allergen representative peptides (ARPs), and hybrid approach. Moreover, by using a four-step approach and comparing the Tanimoto coefficient, AllergenFP predicts the allergenicity of peptides using an alignment-free method.

### Antigenicity of multi-epitope vaccine

VaxiJen v2.0 [56] and ANTIGENpro [70] were used to evaluate the antigenicity of final constructs of multi-epitope vaccine. ANTIGENpro is available online at the Scratch Protein Predictor server http://scratch.proteomics.ics.uci.edu/.

### Immune simulation

Immune simulation analysis of HIV-1a and HIV-1b vaccines was performed using C-IMMSIM https://kraken.iac.rm.cnr.it/C-IMMSIM/ (accessed on August 1, 2022) [71]. Default settings of the tool were used, except HLA-A (HLA-A*0201, HLA-A*2402), HLA-B (HLA-B*0702, HLA-B*0801), and HLA-II (DRB1*0701, DRB1*1501) were selected in the host HLA selection as they have shown higher genotypic frequencies in the worldwide population according to the IEDB analysis resource server.

### Tertiary structure prediction and refinement

HIV-1a and HIV-1b tertiary structures were predicted using the 3Dpro tool hosted at Scratch Protein Predictor [72]. Afterwards, the designed structure was refined using the GalaxyRefine2 tool of GalaxyWEB server https://galaxy.seoklab.org/cgi-bin/submit.cgi?type=REFINE2 [73]. Structure with the most Rama-favored residues and minimum energy was selected for further analysis.

### Model quality analysis and Ramachandran plot analysis

ProSA-web https://prosa.services.came.sbg.ac.at/prosa.php was used to analyze z-score and local model quality [74], while PDBsum http://www.ebi.ac.uk/thornton-srv/databases/pdbsum/Generate.html was used to generate Ramachandran plots of HIV-1a and HIV-1b refined structures [75].

### MD simulation

GROMACS was used as a tool to stabilize the vaccine structure through MD simulation. GROMACS is a tool that gives the real-life environmental conditions for different biological models [76]. OPLS (Optimized Potential for Liquid Simulation) was applied as a force-field, and the proteins were contained in the rhombic dodecahedron cubic box to remain intact with the solvent molecules. With a distance of 2 nm from the boundaries, the proteins were put in the cube’s center. With the force constant (kpr) of 1,000,000 J/mol-1/nm-2, the solvent water spc216 was used for protein simulation. The ion balancing was performed with the help of built-in Genion tool, in which cutoff scheme Verlet was used, while the electrostatic forces being applied. Then, energy minimization was carried out, and the final structure with Energy Minimization (EM) was obtained. The graphic visualization of the results was generated using qtgrace software. NVT isothermal-isochoric ensemble equilibration at 100 ps was used to stabilize the temperature of proteins up to a specified value. During NVT equilibration, velocity was created so that a range of simulations may run at different speeds. Temperature, pressure, and density of the stabilized vaccine constructs were examined using an NPT ensemble with 50,000 steps for the entire process. For the equilibrated construct, an MD simulation was run at 100 ns with 50,000,000 steps. The RMSD of the backbone of energy was minimized, and the findings were generated in the form of graphs. The radius of gyration and density plots were also determined along with the RMSF protocol performance for MD simulation.

### Secondary structure prediction

PDBsum http://www.ebi.ac.uk/thornton-srv/databases/pdbsum/Generate.html was used to generate the secondary structure and topology of both vaccine constructs [75].

### Molecular interaction with TLR-3

For docking of TLR-3 with HIV-1a and HIV-1b, extracellular domain structure of TLR-3 was downloaded from the RCSB PDB (PDB ID: 1ZIW) [77]. CPORT [64] was used to determine active and passive residues of TLR-3, HIV-1a and HIV-1b. Afterwards, docking of HIV-1a and HIV-1b with TLR-3 was performed using the HADDOCK 2.4 GURU level interface with default values [65]. Then, HADDOCK refinement interface https://wenmr.science.uu.nl/haddock2.4/refinement/1 [65] with default values was used to refine HIV-1a-TLR-3 and HIV-1b-TLR-3 complexes. Normal Mode Analysis (NMA) of complexes was also performed using the iMODS server https://imods.iqfr.csic.es/ [78] to determine the deformation potential of both constructs. Finally, PDBsum [75] was used to perform a detailed analysis of protein–protein interactions in complexes.

### Codon optimization and *in-silico* cloning

JCat http://www.jcat.de/ [79] was used to perform codon optimization of HIV-1a and HIV-1b. Codons were adapted according to the K12 strain of *E. coli* using default values. BamH1 and Xho1 restriction sites were added to the N and C terminal of optimized sequences of vaccines respectively. pET28(a) + plasmid was downloaded from the SnapGene database. Optimized sequences with restriction sites were reversed and then cloned to the plasmid by using SnapGene 5.2.4 [13].

## Supplementary Information

**Additional file 1:** Supplementary data for this article is present in Supplementary file 1.pdf, and it contains four tables, i.e., **Table S1**, **Table S2**, **Table S3**, and **Table S4**, comprising of conservational analysis of HIV-1 sequences, epitopes binding capacity with HLA molecules, HADDOCK scores, and accession IDs of retrieved HIV-1 sequences, respectively.

## Data Availability

HIV-1 polyprotein and protein sequences are available and retrieved from http://www.hiv.lanl.gov/ on 4^th^ December 2020 (accession IDs are listed in Supplementary Table S4). Structures of HIV-1 nucleocapsid (PDB ID: 5I1R), HIV-1 reverse transcriptase (PDB ID 1HMV), HIV-1 integrase (PDB ID 6U8Q), HIV-1 VPU (PDB ID 1VPU), and TLR-3 (1ZIW) were downloaded from the RCSB PDB https://www.rcsb.org/. The amino acid sequence of HBD-3 (Accession ID: Q5U7J2) was retrieved from https://www.uniprot.org/uniprotkb/Q5U7J2/entry.

## Abbreviations

AIDS: Acquired immunodeficiency syndrome
ARPs: Allergen representative peptides
ART: Anti-retroviral therapy
BLAST: Basic local alignment search tool
CAI: Codon adaptation index
CD4 +: Cluster of differentiation 4
CD8 +: Cluster of differentiation 8
COVID: Coronavirus disease
ENV: Envelope
E-value: Expect value
GAG: Group specific antigen
GRAVY: Grand average of hydropathy
HADDOCK: High ambiguity driven protein–protein docking
HBD-3: Human β-defensin-3
HIV-1: Human immunodeficiency virus-1
HLA: Human leukocyte antigen
IEDB: Immune epitope database
IFN-ɣ: Interferon-gamma
IgE: Immunoglobulin E
IL: Interleukin
iMODS: Internal coordinates normal mode analysis
Int: Integrase
LANL: Los Alamos national laboratory
MAFFT: Multiple alignment using fast Fourier transform
MD: Molecular dynamics
MERS: Middle east respiratory syndrome
NC: Nucleocapsid
NCBI: National center for biotechnology information
NMA: Normal mode analysis
NMR: Nuclear magnetic resonance
PDB: Protein data bank
Phyre2: Protein fold recognition server
pI: Isoelectric point
POL: Polymerase
ProSA: Protein structure analysis
RCSB: Research Collaboratory for structural bioinformatics
REV: Regulator of expression of virion proteins
RMSD: Root mean square deviation
RMSF: Root mean square fluctuation
RT: Reverse transcriptase
SVM: Support vector machine
TAT: Trans-activator of transcription
Th1: T-helper cell 1
Th2: T-helper cell 2
TLR: Toll like receptor
TRIF: TIR-domain-containing adapter-inducing interferon- β
VIF: Viral infectivity factor
VPR: Viral protein R
VPU: Viral protein U

## References

[1] Data U. Available online: https://www.unaids.org/sites/default/files/media_asset2020.

[2] Organization WH. Policy brief: update of recommendations on first-and second-line antiretroviral regimens. Geneva: World Health Organization; 2019.

[3] Burton DR (2019). Advancing an HIV vaccine; advancing vaccinology. Nat Rev Immunol.

[4] Ella KM, Mohan VK (2020). Coronavirus vaccine: light at the end of the tunnel. Indian Pediatr..

[5] Yan L, Yu F, Zhang H, Zhao H, Wang L, Liang Z (2020). Transmitted and acquired HIV-1 drug resistance from a family: a case study. Infect Drug Resist.

[6] Martinez-Steele E, Awasana AA, Corrah T, Sabally S, van der Sande M, Jaye A (2007). Is HIV-2-induced AIDS different from HIV-1-associated AIDS? Data from a West African clinic. AIDS.

[7] Grabar S, Selinger-Leneman H, Abgrall S, Pialoux G, Weiss L, Costagliola D (2009). Prevalence and comparative characteristics of long-term nonprogressors and HIV controller patients in the French Hospital Database on HIV. AIDS.

[8] Kumar P (2013). Long term non-progressor (LTNP) HIV infection. Indian J Med Res.

[9] Yang Y, Sun W, Guo J, Zhao G, Sun S, Yu H (2015). In silico design of a DNA-based HIV-1 multi-epitope vaccine for Chinese populations. Hum Vaccin Immunother.

[10] Gorse GJ, Baden LR, Wecker M, Newman MJ, Ferrari G, Weinhold KJ (2008). Safety and immunogenicity of cytotoxic T-lymphocyte poly-epitope, DNA plasmid (EP HIV-1090) vaccine in healthy, human immunodeficiency virus type 1 (HIV-1)-uninfected adults. Vaccine.

[11] Pavlakis GN, Felber BK (2018). A new step towards an HIV/AIDS vaccine. The Lancet.

[12] Mohan T, Mitra D, Rao D (2014). Nasal delivery of PLG microparticle encapsulated defensin peptides adjuvanted gp41 antigen confers strong and long-lasting immunoprotective response against HIV-1. Immunol Res.

[13] Pandey RK, Ojha R, Aathmanathan VS, Krishnan M, Prajapati VK (2018). Immunoinformatics approaches to design a novel multi-epitope subunit vaccine against HIV infection. Vaccine.

[14] Joshi A, Ray NM, Singh J, Upadhyay AK, Kaushik V (2022). T-cell epitope-based vaccine designing against Orthohantavirus: a causative agent of deadly cardio-pulmonary disease. Netw Model Anal Health Inform Bioinform.

[15] Joshi A, Krishnan S, Kaushik V (2022). Codon usage studies and epitope-based peptide vaccine prediction against Tropheryma whipplei. J Genet Eng Biotechnol.

[16] Ikram A, Zaheer T, Awan FM, Obaid A, Naz A, Hanif R (2018). Exploring NS3/4A, NS5A and NS5B proteins to design conserved subunit multi-epitope vaccine against HCV utilizing immunoinformatics approaches. Sci Rep.

[17] Zaheer T, Waseem M, Waqar W, Dar HA, Shehroz M, Naz K (2020). Anti-COVID-19 multi-epitope vaccine designs employing global viral genome sequences. PeerJ.

[18] Shey RA, Ghogomu SM, Esoh KK, Nebangwa ND, Shintouo CM, Nongley NF (2019). In-silico design of a multi-epitope vaccine candidate against onchocerciasis and related filarial diseases. Sci Rep.

[19] Srivastava S, Kamthania M, Singh S, Saxena AK, Sharma N (2018). Structural basis of development of multi-epitope vaccine against middle east respiratory syndrome using in silico approach. Infect Drug Resist.

[20] Saadi M, Karkhah A, Nouri HR (2017). Development of a multi-epitope peptide vaccine inducing robust T cell responses against brucellosis using immunoinformatics based approaches. Infect Genet Evol.

[21] Lin X, Chen S, Xue X, Lu L, Zhu S, Li W (2016). Chimerically fused antigen rich of overlapped epitopes from latent membrane protein 2 (LMP2) of Epstein-Barr virus as a potential vaccine and diagnostic agent. Cell Mol Immunol.

[22] Oyarzún P, Kobe B (2016). Recombinant and epitope-based vaccines on the road to the market and implications for vaccine design and production. Hum Vaccin Immunother.

[23] Amanna IJ, Slifka MKJV (2011). Contributions of humoral and cellular immunity to vaccine-induced protection in humans. Virology.

[24] Freed EO (2001). HIV-1 replication. Somat Cell Mol Genet.

[25] Andrew A, Strebel K, Vicenzi E, Poli G (2014). HIV-1 accessory proteins: Vpu and Vif. Human Retroviruses: Methods and Protocols.

[26] Schwartz S, Felber B, Benko D, Fenyö E, Pavlakis G (1990). Cloning and functional analysis of multiply spliced mRNA species of human immunodeficiency virus type 1. J Virol.

[27] McElrath MJ, Haynes BF (2010). Induction of immunity to human immunodeficiency virus type-1 by vaccination. Immunity.

[28] McBurney SP, Ross TM (2008). Viral sequence diversity: challenges for AIDS vaccine designs. Expert Rev Vaccines.

[29] Van Regenmortel MHJM (1996). Mapping epitope structure and activity: from one-dimensional prediction to four-dimensional description of antigenic specificity. Methods.

[30] Newman MJ, Livingston B, McKinney DM, Chesnut RW, Sette A, Subsets-immunology TJFB (2002). T-lymphocyte epitope identification and their use in vaccine development for HIV-1. Front Biosci.

[31] Dominguez C, Boelens R, Bonvin AM (2003). HADDOCK: a protein− protein docking approach based on biochemical or biophysical information. J Am Chem Soc.

[32] De Vries SJ, Van Dijk M, Bonvin AM (2010). The HADDOCK web server for data-driven biomolecular docking. Nat Protoc.

[33] Pandey RK, Bhatt TK, Prajapati VK (2018). Novel immunoinformatics approaches to design multi-epitope subunit vaccine for malaria by investigating anopheles salivary protein. Sci Rep.

[34] Mohan T, Sharma C, Bhat AA, Rao DJV (2013). Modulation of HIV peptide antigen specific cellular immune response by synthetic α-and β-defensin peptides. Vaccine.

[35] Tani K, Murphy WJ, Chertov O, Salcedo R, Koh CY, Utsunomiya I (2000). Defensins act as potent adjuvants that promote cellular and humoral immune responses in mice to a lymphoma idiotype and carrier antigens. Int Immunol.

[36] Arai R, Ueda H, Kitayama A, Kamiya N, Nagamune T (2001). Design of the linkers which effectively separate domains of a bifunctional fusion protein. Protein Eng Des Sel.

[37] Zhou Y, Wang X, Liu M, Hu Q, Song L, Ye L (2010). A critical function of toll-like receptor-3 in the induction of anti-human immunodeficiency virus activities in macrophages. Immunology.

[38] Saxena M, Sabado RL, La Mar M, Mohri H, Salazar AM, Dong H (2019). Poly-ICLC, a TLR3 agonist, induces transient innate immune responses in patients with treated HIV-infection: a randomized double-blinded placebo controlled trial. Front Immunol.

[39] Gauzzi MC, Del Cornò M, Gessani S (2010). Dissecting TLR3 signalling in dendritic cells. Immunobiology.

[40] Abdulla F, Adhikari UK, Uddin MK (2019). Exploring T & B-cell epitopes and designing multi-epitope subunit vaccine targeting integration step of HIV-1 lifecycle using immunoinformatics approach. Microb Pathog.

[41] Yang D, Chertov O, Bykovskaia S, Chen Q, Buffo M, Shogan J (1999). β-defensins: linking innate and adaptive immunity through dendritic and T cell CCR6. Science.

[42] Hoshino S, Konishi M, Mori M, Shimura M, Nishitani C, Kuroki Y (2010). HIV-1 Vpr induces TLR4/MyD88-mediated IL-6 production and reactivates viral production from latency. J Leukoc Biol.

[43] Henrick BM, Yao XD, Zahoor MA, Abimiku Al, Osawe S, Rosenthal KL (2019). TLR10 senses HIV-1 proteins and significantly enhances HIV-1 infection. Front Immunol.

[44] Katoh K, Rozewicki J, Yamada KD (2019). MAFFT online service: multiple sequence alignment, interactive sequence choice and visualization. Brief Bioinform.

[45] Matvienko M, editor CLC Genomics Workbench. Plant and Animal Genome Conference Qiagen Bioinformatics Workshop at PAG; 2015.

[46] Magierowska M, Theodorou I, Debré P, Sanson F, Autran B, Rivière Y (1999). Combined genotypes of CCR5, CCR2, SDF1, and HLA genes can predict the long-term nonprogressor status in human immunodeficiency virus-1–infected individuals. Blood.

[47] Selvaraj P, Swaminathan S, Alagarasu K, Raghavan S, Narendran G, Narayanan P (2006). Association of human leukocyte antigen-A11 with resistance and B40 and DR2 with susceptibility to HIV-1 infection in south India. J Acquir Immune Defic Syndr.

[48] Adland E, Hill M, Lavandier N, Csala A, Edwards A, Chen F (2018). Differential immunodominance hierarchy of CD8+ T-cell responses in HLA-B* 27: 05-and-B* 27: 02-mediated control of HIV-1 infection. J Virol.

[49] Salgado M, Simón A, Sanz-Minguela B, Rallón NI, López M, Vicario JL (2011). An additive effect of protective host genetic factors correlates with HIV nonprogression status. J Acquir Immune Defic Syndr.

[50] Kawashima Y, Pfafferott K, Frater J, Matthews P, Payne R, Addo M (2009). Adaptation of HIV-1 to human leukocyte antigen class I. Nature.

[51] Munkanta M, Terunuma H, Takahashi M, Hanabusa H, Miura T, Ikeda S (2005). HLA-B Polymorphism in Japanese HIV-1–infected long-term surviving hemophiliacs. Viral Immunol.

[52] Chen Y, Winchester R, Korber B, Gagliano J, Bryson Y, Hutto C (1997). Influence of HLA alleles on the rate of progression of vertically transmitted HIV infection in children: association of several HLA-DR13 alleles with long-term survivorship and the potential association of HLA-A2301 with rapid progression to AIDS. Hum Immunol.

[53] Ranasinghe S, Cutler S, Davis I, Lu R, Soghoian DZ, Qi Y (2013). Association of HLA-DRB1–restricted CD4+ T cell responses with HIV immune control. Nat Med.

[54] Okonechnikov K, Golosova O, Fursov M, Team U (2012). Unipro UGENE: a unified bioinformatics toolkit. Bioinformatics.

[55] Jespersen MC, Peters B, Nielsen M, Marcatili P (2017). BepiPred-2.0: improving sequence-based B-cell epitope prediction using conformational epitopes. Nucleic Acids Res.

[56] Doytchinova IA, Flower DR (2007). VaxiJen: a server for prediction of protective antigens, tumour antigens and subunit vaccines. BMC Bioinformatics.

[57] Altschul SF, Gish W, Miller W, Myers EW, Lipman DJ (1990). Basic local alignment search tool. J Mol Biol.

[58] Bui HH, Sidney J, Dinh K, Southwood S, Newman MJ, Sette AJ (2006). Predicting population coverage of T-cell epitope-based diagnostics and vaccines. BMC Bioinformatics.

[59] Deshmukh L, Schwieters CD, Grishaev A, Clore GM (2016). Quantitative Characterization of Configurational Space Sampled by HIV-1 Nucleocapsid Using Solution NMR, X-ray Scattering and Protein Engineering. ChemPhysChem.

[60] Rodgers D, Gamblin S, Harris B, Ray S, Culp J, Hellmig B (1995). The structure of unliganded reverse transcriptase from the human immunodeficiency virus type 1. Proc Natl Acad Sci.

[61] Waqar W, Altaf S, Nazir S, Javed A (2020). Novel association of genetic variants in non-coding regulatory regions with HIV-1 infection. Infect Genet Evol.

[62] Willbold D, Hoffmann S, Rösch P (1997). Secondary structure and tertiary fold of the human immunodeficiency virus protein U (Vpu) cytoplasmic domain in solution. Eur J Biochem.

[63] Pettersen EF, Goddard TD, Huang CC, Meng EC, Couch GS, Croll TI (2021). UCSF ChimeraX: Structure visualization for researchers, educators, and developers. Protein Sci.

[64] de Vries SJ, Bonvin AM (2011). CPORT: a consensus interface predictor and its performance in prediction-driven docking with HADDOCK. PLoS One.

[65] Van Zundert G, Rodrigues J, Trellet M, Schmitz C, Kastritis P, Karaca E (2016). The HADDOCK2. 2 web server: user-friendly integrative modeling of biomolecular complexes. J Mol Biol.

[66] Kelley LA, Mezulis S, Yates CM, Wass MN, Sternberg MJ (2015). The Phyre2 web portal for protein modeling, prediction and analysis. Nat Protoc.

[67] Gasteiger E, Hoogland C, Gattiker A, Duvaud Se, Wilkins MR, Appel RD, et al. Protein identification and analysis tools on the ExPASy server. In: Walker JM, editor. The Proteomics Protocols Handbook. Totowa: Humana Press; 2005. p. 571–607.

[68] Dimitrov I, Naneva L, Doytchinova I, Bangov I (2014). AllergenFP: allergenicity prediction by descriptor fingerprints. Bioinformatics.

[69] Saha S, Raghava G (2006). AlgPred: prediction of allergenic proteins and mapping of IgE epitopes. Nucleic Acids Res.

[70] Magnan CN, Zeller M, Kayala MA, Vigil A, Randall A, Felgner PL (2010). High-throughput prediction of protein antigenicity using protein microarray data. Bioinformatics.

[71] Rapin N, Lund O, Bernaschi M, Castiglione F (2010). Computational immunology meets bioinformatics: the use of prediction tools for molecular binding in the simulation of the immune system. PLoS One.

[72] Cheng J, Randall AZ, Sweredoski MJ, Baldi P (2005). SCRATCH: a protein structure and structural feature prediction server. Nucleic Acids Res.

[73] Ko J, Park H, Heo L, Seok C (2012). GalaxyWEB server for protein structure prediction and refinement. Nucleic Acids Res.

[74] Wiederstein M, Sippl MJ (2007). ProSA-web: interactive web service for the recognition of errors in three-dimensional structures of proteins. Nucleic Acids Res.

[75] Laskowski RA, Jabłońska J, Pravda L, Vařeková RS, Thornton JM (2018). PDBsum: Structural summaries of PDB entries. Protein Sci.

[76] Abraham MJ, Murtola T, Schulz R, Páll S, Smith JC, Hess B (2015). GROMACS: High performance molecular simulations through multi-level parallelism from laptops to supercomputers. SoftwareX.

[77] Choe J, Kelker MS, Wilson IA (2005). Crystal structure of human toll-like receptor 3 (TLR3) ectodomain. Science.

[78] López-Blanco JR, Aliaga JI, Quintana-Ortí ES, Chacón P (2014). iMODS: internal coordinates normal mode analysis server. Nucleic Acids Res.

[79] Grote A, Hiller K, Scheer M, Münch R, Nörtemann B, Hempel DC (2005). JCat: a novel tool to adapt codon usage of a target gene to its potential expression host. Nucleic Acids Res.

