# Supplementary material for: Employing computational tools to design a multi-epitope vaccine targeting human immunodeficiency virus-1 (HIV-1)
Source: BMC Genomics. 2023 May 24;24:276. doi: 10.1186/s12864-023-09330-4 (PMC10206567; doi:10.1186/s12864-023-09330-4)
Supplement: Supplementary file 1 — Additional file 1: Supplementary data for this article is present in Supplementary file 1.pdf, and it contains four tables, i.e., Table S1, Table S2, Table S3, and Table S4, comprising of conservational analysis of HIV-1 sequences, epitopes binding capacity with HLA molecules, HADDOCK scores, and accession IDs of retrieved HIV-1 sequences, respectively. [file 12864_2023_9330_MOESM1_ESM.pdf]

Table S1: Summary of retrieved sequences and the conservational analysis of their consensus sequences with respective reference sequences.

Sr. No.	Proteins and polyproteins	No. of downloaded sequences	Reference sequence accession no.	Consensus mismatches	Consensus gaps	Consensus conservancy (%)
				With reference sequence		
1	GAG	19,492	NP_057850.1	29	1	94
2	POL	10,062	NP_789740.1	42	1	95.68
3	VIF	14,332	NP_057851.1	12	0	93.75
4	VPR	14,474	NP_057852.2	6	0	93.75
5	TAT	11,737	NP_057853.1	13	0	84.88
6	REV	11,951	NP_057854.1	13	0	88.79
7	VPU	16,021	NP_057855.1	17	1	78.05
8	ENV	20,648	NP_057856.1	95	24	86.16
9	NEF	18,079	NP_057857.2	31	0	84.95

Table S2: HLA class I and HLA class II molecules binding with selected epitopes.

Sr. No.	Epitopes	HLA class I molecules	HLA class II molecules
**1**	APRKKGCWK	HLA-A*0205, HLA-A*2402, HLA-B*2706	
**2**	PRKKGCWKC	HLA-B*2702	
**3**	RKKGCWKCG	HLA-A2	
**4**	PLTEEKIKA	HLA-A2	
**5**	KRTQDFWEV	HLA-B*2702	
**6**	RTQDFWEVQ	HLA-A*0206, HLA-A3, HLA-A11, HLA-A31, HLA-A*3301, HLA-B44	
**7**	TQDFWEVQL	HLA-A1, HLA-B*2702, HLA-B*2705, HLA-B*3801, HLA-B*3902, HLA-B35, HLA-B*2703	
**8**	AHKGIGGNE	HLA-A*2402, HLA-B8, HLA-B*51, HLA-A*3301	
**9**	FRVYYRDSR	HLA-A20, HLA-B*2702	HLA-DRB1*0309, HLA-DRB1*0801, HLA-DRB1*0802, HLA-DRB1*0813, HLA-DRB1*1114, HLA-DRB1*1120, HLA-DRB1*1302, HLA-DRB1*1323, HLA-DRB1*1501, HLA-DRB1*1502, HLA-DRB1*1506
**10**	VYYRDSRDP		HLA-DRB1*0402, HLA-DRB1*0802, HLA-DRB1*0804, HLA-DRB1*0806, HLA-DRB1*1301, HLA-DRB1*1327, HLA-DRB1*1328
**11**	ERAEDSGNE	HLA-B*3501, HLA-B*51, HLA-B*5301, HLA-B*5401, HLA-A*3301	

Table S3: Haddock score for different combinations of epitope.

Combination of 2	HADDOCK score	Combination of 3	HADDOCK score
NC1-NC2	-60.9 +/- 4.5	NC2-RT4-NC1	-68.3 +/- 3.9
NC1-NC3	-67.9 +/- 3.1	NC2-RT4-NC3	-66.3 +/- 0.7
NC1-RT1	-48.8 +/- 6.3	NC2-RT4-RT1	-58.3 +/- 6.9
NC1-RT2	-82.6 +/- 3.6	NC2-RT4-RT2	-84.0 +/- 4.2
NC1-RT3	-85.8 +/- 9.0	NC2-RT4-RT3	-77.2 +/- 1.8
NC1-RT4	-80.2 +/- 5.3	NC2-RT4-RT5	-56.6 +/- 8.4
NC1-RT5	-73.5 +/- 4.2	NC2-RT4-Int1	-71.1 +/- 1.6
NC1-Int1	-63.6 +/- 2.1	NC2-RT4-Int2	-78.8 +/- 3.9
NC1-Int2	-78.1 +/- 2.5	NC2-RT4-VPU1	-66.6 +/- 9.0
NC1-VPU1	-77.4 +/- 7.4	**Combination of 4**	**HADDOCK score**
NC2-NC3	-63.3 +/- 2.6	NC2-RT4-RT2-NC1	-58.3 +/- 4.5
NC2-RT1	-52.3 +/- 3.9	NC2-RT4-RT2-NC3	-53.9 +/- 3.6
NC2-RT2	-81.9 +/- 5.1	NC2-RT4-RT2-RT1	-34.0 +/- 8.3
NC2-RT3	-82.1 +/- 5.7	NC2-RT4-RT2-RT3	-60.7 +/- 2.8
NC2-RT4	-88.3 +/- 1.1	NC2-RT4-RT2-RT5	-47.1 +/- 4.2
NC2-RT5	-71.4 +/- 1.3	NC2-RT4-RT2-Int1	-67.4 +/- 2.7
NC2-Int1	-77.0 +/- 2.0	NC2-RT4-RT2-Int2	-66.9 +/- 10.0
NC2-Int2	-78.0 +/- 11.8	NC2-RT4-RT2-VPU1	-53.9 +/- 4.5
NC2-VPU1	-77.0 +/- 3.0	**Combination of 5**	**HADDOCK score**
NC3-RT1	-50.0 +/- 5.6	NC2-RT4-RT2-Int1-NC1	-72.7 +/- 3.2
NC3-RT2	-80.7 +/- 6.7	NC2-RT4-RT2-Int1-NC3	-75.9 +/- 4.6
NC3-RT3	-72.5 +/- 1.9	NC2-RT4-RT2-Int1-RT1	-41.5 +/- 1.0
NC3-RT4	-74.6 +/- 1.7	NC2-RT4-RT2-Int1-RT3	-60.9 +/- 11.8
NC3-RT5	-63.3 +/- 1.7	NC2-RT4-RT2-Int1-RT5	-35.0 +/- 8.6
NC3-Int1	-72.3 +/- 0.5	NC2-RT4-RT2-Int1-Int2	-84.0 +/- 3.5
NC3-Int2	-76.0 +/- 4.0	NC2-RT4-RT2-Int1-VPU1	-54.0 +/- 3.9
NC3-VPU1	-77.2 +/- 2.5	**Combination of 6**	**HADDOCK score**
RT1-RT2	-58.7 +/- 2.8	NC2-RT4-RT2-Int1-Int2-NC1	-54.6 +/- 11.5
RT1-RT3	-51.0 +/- 2.5	NC2-RT4-RT2-Int1-Int2-NC3	-63.4 +/- 1.4
RT1-RT4	-55.9 +/- 2.9	NC2-RT4-RT2-Int1-Int2-RT1	-42.4 +/- 4.3
RT1-RT5	-43.8 +/- 0.7	NC2-RT4-RT2-Int1-Int2-RT3	-72.6 +/- 4.6
RT1-Int1	-47.9 +/- 0.6	NC2-RT4-RT2-Int1-Int2-RT5	-46.0 +/- 2.4

RT1-Int2	-64.6 +/- 1.9
RT1-VPU1	-53.4 +/- 0.9
RT2-RT3	-66.0 +/- 1.4
RT2-RT4	-61.6 +/- 1.5
RT2-RT5	-48.1 +/- 3.0
RT2-Int1	-74.1 +/- 2.3
RT2-Int2	-69.2 +/- 3.0
RT2-VPU1	-68.7 +/- 2.0
RT3-RT4	-58.2 +/- 2.8
RT3-RT5	-54.2 +/- 3.2
RT3-Int1	-70.4 +/- 4.7
RT3-Int2	-59.8 +/- 1.5
RT3-VPU1	-51.0 +/- 4.5
RT4-RT5	-58.5 +/- 2.5
RT4-Int1	-78.5 +/- 1.9
RT4-Int2	-61.2 +/- 1.9
RT4-VPU1	-55.2 +/- 3.0
RT5-Int1	-49.9 +/- 5.5
RT5-Int2	-64.5 +/- 5.6
RT5-VPU1	-49.9 +/- 3.0
Int1-Int2	-70.0 +/- 3.9
Int1-VPU1	-55.2 +/- 6.1
Int2-VPU1	-67.2 +/- 4.0

NC2-RT4-RT2-Int1-Int2-VPU1	-53.2 +/- 4.2
**Combination of 7**	**HADDOCK score**
NC2-RT4-RT2-Int1-Int2-RT3-NC1	-61.7 +/- 2.2
NC2-RT4-RT2-Int1-Int2-RT3-NC3	-56.2 +/- 5.8
NC2-RT4-RT2-Int1-Int2-RT3-RT1	-47.4 +/- 7.0
NC2-RT4-RT2-Int1-Int2-RT3-RT5	-41.8 +/- 6.8
NC2-RT4-RT2-Int1-Int2-RT3-VPU1	-41.7 +/- 4.5
**Combination of 8**	**HADDOCK score**
NC2-RT4-RT2-Int1-Int2-RT3-NC1-NC3	-52.2 +/- 4.2
NC2-RT4-RT2-Int1-Int2-RT3--NC1-RT1	-36.0 +/- 3.1
NC2-RT4-RT2-Int1-Int2-RT3-NC1-RT5	-41.0 +/- 4.3
NC2-RT4-RT2-Int1-Int2-RT3-NC1-VPU1	-42.6 +/- 2.2
**Combination of 9**	**HADDOCK score**
NC2-RT4-RT2-Int1-Int2-RT3-NC1-NC3-RT1	-46.5 +/- 2.0
NC2-RT4-RT2-Int1-Int2-RT3-NC1-NC3-RT5	-54.0 +/- 4.8
NC2-RT4-RT2-Int1-Int2-RT3-NC1-NC3-VPU1	-60.9 +/- 9.0
**Combination of 10**	**HADDOCK score**
NC2-RT4-RT2-Int1-Int2-RT3-NC1-NC3-VPU1-RT1	-58.4 +/- 7.7
NC2-RT4-RT2-Int1-Int2-RT3-NC1-NC3-VPU1-RT5	-43.1 +/- 6.7
**Combination of 11**	**HADDOCK score**
NC2-RT4-RT2-Int1-Int2-RT3-NC1-NC3-VPU1-RT1-RT5	-46.4 +/- 1.7

Table S4: Accession ID of HIV-1 polyprotein and protein sequences.

K03455	HM639117	KT878024	MK272390	MN635398	MT194284
A04321	HM639160	KT878025	MK272391	MN635399	MT194285
AB023804	HM639204	KT878026	MK272392	MN635400	MT194286
AB032740	HM769943	KT878027	MK272393	MN643057	MT194287
AB032741	HM771423	KT878028	MK272394	MN650487	MT194288
AB049811	HM776938	KT878029	MK272395	MN650503	MT194289
AB052867	HM776939	KT878030	MK272396	MN650512	MT194290
AB052995	HQ110664	KT878031	MK272397	MN650531	MT194291
AB070352	HQ122397	KT878032	MK272398	MN654104	MT194292
AB078005	HQ179987	KT878033	MK272399	MN654105	MT194293
AB097865	HQ215552	KT878034	MK272400	MN654106	MT194294
AB097866	HQ215553	KT878035	MK272401	MN654107	MT194295
AB097867	HQ215554	KT878036	MK272402	MN654108	MT194297
AB097868	HQ215555	KT878037	MK272403	MN654109	MT194298
AB097869	HQ215556	KT878038	MK272404	MN659380	MT194300
AB097870	HQ216367	KT878039	MK272405	MN659381	MT194301
AB097871	HQ216397	KT878040	MK272406	MN690612	MT194302
AB097872	HQ216471	KT896551	MK272407	MN690613	MT194303
AB097873	HQ216501	KT896565	MK272408	MN690614	MT194304
AB098330	HQ216557	KT960983	MK272409	MN690615	MT194305
AB098332	HQ216577	KT961002	MK272410	MN690616	MT194306
AB220944	HQ216601	KT982201	MK272411	MN690617	MT194307
AB220945	HQ216632	KT983615	MK272412	MN690618	MT194308
AB220946	HQ216675	KT986553	MK272413	MN690619	MT194309
AB220947	HQ216702	KT986933	MK272414	MN690620	MT194310
AB220948	HQ216730	KT999999	MK272415	MN690621	MT194311
AB221005	HQ216755	KU051564	MK272416	MN690622	MT194312
AB221125	HQ216785	KU161143	MK272417	MN690623	MT194314
AB231893	HQ216810	KU161144	MK272418	MN690624	MT194315
AB231894	HQ216841	KU161145	MK272419	MN690625	MT194316
AB231895	HQ216861	KU168256	MK272420	MN690626	MT194317
AB231896	HQ216893	KU168257	MK272421	MN690627	MT194318
AB231897	HQ216915	KU168258	MK272422	MN690628	MT194319
AB231898	HQ216949	KU168259	MK272423	MN690629	MT194320
AB253421	HQ216971	KU168260	MK272424	MN690630	MT194321
AB253423	HQ217019	KU168261	MK272425	MN690631	MT194322
AB253424	HQ217043	KU168262	MK272426	MN690632	MT194323
AB253426	HQ217079	KU168263	MK272427	MN690633	MT194324
AB253428	HQ217099	KU168264	MK272428	MN690634	MT194325
AB253430	HQ217115	KU168265	MK272429	MN690635	MT194326
AB253635	HQ217144	KU168266	MK272430	MN690636	MT194327
AB253647	HQ217167	KU168267	MK272431	MN690637	MT194328
AB253659	HQ217193	KU168268	MK272432	MN690638	MT194329
AB253692	HQ217256	KU168269	MK272433	MN690639	MT194330
AB253703	HQ217283	KU168270	MK272434	MN690640	MT194331
AB254141	HQ217315	KU168271	MK272435	MN690641	MT194332

AB254142	HQ217340	KU168272	MK272436	MN690642	MT194333
AB254143	HQ217368	KU168273	MK272438	MN690643	MT194334
AB254146	HQ217393	KU168274	MK272439	MN690644	MT194335
AB254148	HQ217418	KU168275	MK272440	MN690645	MT194336
AB254149	HQ217443	KU168276	MK272539	MN690646	MT194337
AB254150	HQ217474	KU168277	MK272540	MN690647	MT194338
AB254155	HQ217505	KU168278	MK272541	MN690648	MT194339
AB254156	HQ217541	KU168279	MK272542	MN690649	MT194340
AB262952	HQ217574	KU168280	MK272543	MN690650	MT194343
AB286849	HQ217651	KU168281	MK272544	MN690651	MT194344
AB286851	HQ217708	KU168282	MK272545	MN690652	MT194345
AB286853	HQ217733	KU168283	MK272546	MN690653	MT194346
AB286855	HQ217760	KU168284	MK272547	MN690654	MT194347
AB286857	HQ217784	KU168285	MK272548	MN690655	MT194348
AB286859	HQ217818	KU168286	MK272549	MN690656	MT194349
AB286862	HQ217840	KU168288	MK272550	MN690657	MT194350
AB286863	HQ217860	KU168289	MK272551	MN690658	MT194351
AB286955	HQ217874	KU168290	MK272552	MN690659	MT194352
AB287363	HQ217892	KU168291	MK272553	MN690660	MT194353
AB287364	HQ217914	KU168292	MK272554	MN690661	MT194354
AB287366	HQ217930	KU168293	MK272555	MN690662	MT194355
AB287368	HQ217947	KU168294	MK272556	MN690663	MT194356
AB287369	HQ217967	KU168295	MK272557	MN690664	MT194357
AB287376	HQ217984	KU168296	MK272558	MN690665	MT194358
AB287378	HQ218001	KU168297	MK272559	MN690666	MT194359
AB289587	HQ218020	KU168298	MK272560	MN690667	MT194360
AB289589	HQ218034	KU168299	MK272561	MN690668	MT194361
AB428551	HQ225812	KU168300	MK272562	MN690669	MT194362
AB428552	HQ236564	KU168301	MK272563	MN690670	MT194363
AB428553	HQ236569	KU168302	MK272564	MN690671	MT194364
AB428554	HQ236571	KU168303	MK272565	MN690672	MT194365
AB428555	HQ236575	KU168304	MK272566	MN690673	MT194366
AB428556	HQ236576	KU168305	MK272567	MN690674	MT194369
AB428557	HQ236581	KU168306	MK272568	MN690675	MT194370
AB428558	HQ236587	KU168307	MK272569	MN690676	MT194371
AB428559	HQ236594	KU168308	MK272570	MN690677	MT194372
AB428560	HQ236606	KU168309	MK272571	MN690678	MT194373
AB428561	HQ236607	KU168310	MK272572	MN690679	MT194374
AB428562	HQ236608	KU168311	MK272573	MN690680	MT194375
AB480044	HQ236609	KU200869	MK272574	MN690681	MT194376
AB480047	HQ236610	KU230417	MK272575	MN690682	MT194377
AB480298	HQ236611	KU230418	MK272576	MN690683	MT194378
AB480300	HQ236615	KU230419	MK272577	MN690684	MT194379
AB480692	HQ236619	KU230420	MK272578	MN690685	MT194380
AB480694	HQ238279	KU230421	MK272579	MN690686	MT194381
AB480696	HQ326124	KU230422	MK272580	MN690687	MT194382
AB480698	HQ326125	KU230423	MK272581	MN690688	MT194383

AB485632	HQ326126	KU230424	MK272582	MN690689	MT194384
AB485633	HQ326127	KU230426	MK272583	MN690690	MT194385
AB485634	HQ326129	KU230430	MK272584	MN690691	MT194386
AB485636	HQ326130	KU230432	MK272585	MN690692	MT194387
AB485638	HQ326131	KU230433	MK272586	MN690693	MT194388
AB485641	HQ326132	KU230434	MK272587	MN690694	MT194389
AB485643	HQ326133	KU230435	MK272588	MN690695	MT194390
AB485645	HQ326134	KU230436	MK272589	MN690696	MT194391
AB485648	HQ326135	KU230437	MK272590	MN690697	MT194392
AB485650	HQ326136	KU252650	MK272591	MN690698	MT194393
AB485652	HQ326137	KU310618	MK272592	MN690699	MT194394
AB485654	HQ326138	KU310619	MK272593	MN690700	MT194395
AB485656	HQ326139	KU310620	MK272594	MN690701	MT194396
AB485658	HQ326140	KU319528	MK272595	MN690702	MT194397
AB485660	HQ326141	KU319529	MK272596	MN690703	MT194398
AB485662	HQ326142	KU319530	MK272597	MN690704	MT194399
AB485664	HQ326143	KU319531	MK272598	MN690705	MT194400
AB485666	HQ326144	KU319532	MK272599	MN690706	MT194401
AB485668	HQ326146	KU319533	MK272600	MN690707	MT194402
AB547463	HQ338111	KU319534	MK272601	MN690708	MT194403
AB547464	HQ338112	KU319535	MK272602	MN690709	MT194404
AB564744	HQ338113	KU319536	MK272603	MN690710	MT194405
AB564746	HQ338115	KU319537	MK272604	MN690711	MT194406
AB565478	HQ338116	KU319538	MK272605	MN690712	MT194407
AB565495	HQ338117	KU319539	MK272606	MN690713	MT194408
AB565497	HQ377375	KU319540	MK272607	MN690714	MT194409
AB565499	HQ377411	KU319541	MK272608	MN690715	MT194410
AB565501	HQ377435	KU319542	MK272609	MN690716	MT194411
AB565503	HQ377459	KU319543	MK272610	MN690717	MT194412
AB588196	HQ377483	KU319544	MK272611	MN690718	MT194413
AB588207	HQ385443	KU319545	MK272612	MN690719	MT194414
AB588209	HQ385444	KU319546	MK272613	MN690720	MT194415
AB588211	HQ385445	KU319547	MK272614	MN690721	MT194416
AB588223	HQ385446	KU319548	MK272615	MN690722	MT194417
AB588232	HQ385447	KU319549	MK272616	MN690723	MT194418
AB588243	HQ385448	KU319550	MK272617	MN690724	MT194419
AB588253	HQ385449	KU319551	MK272618	MN690725	MT194420
AB588263	HQ385450	KU356857	MK272619	MN690726	MT194421
AB588271	HQ385451	KU501256	MK272620	MN690727	MT194422
AB588283	HQ385452	KU501257	MK272621	MN690728	MT194423
AB588291	HQ385453	KU562843	MK272622	MN690729	MT194424
AB588311	HQ385454	KU600814	MK272623	MN690730	MT194425
AB588329	HQ385455	KU600815	MK272624	MN690731	MT194426
AB604946	HQ385456	KU600816	MK272625	MN690732	MT194427
AB604948	HQ385457	KU600817	MK272626	MN690733	MT194428
AB604950	HQ385458	KU600818	MK272627	MN690734	MT194429
AB641836	HQ385459	KU612900	MK272628	MN691961	MT194430

AB646289	HQ385460	KU612901	MK272629	MN692145	MT194431
AB703607	HQ385461	KU641402	MK272630	MN692146	MT194432
AB703608	HQ385462	KU677989	MK272631	MN692147	MT194433
AB703609	HQ385477	KU678025	MK272632	MN692148	MT194434
AB703610	HQ385478	KU678049	MK272633	MN692189	MT194435
AB703611	HQ385479	KU678069	MK272634	MN694995	MT194436
AB703612	HQ385811	KU678125	MK272635	MN695006	MT194437
AB703613	HQ385818	KU678139	MK272636	MN703127	MT194438
AB703614	HQ385819	KU678161	MK272637	MN703128	MT194439
AB703615	HQ385820	KU685581	MK272638	MN703129	MT194440
AB703616	HQ385821	KU685582	MK272639	MN703130	MT194441
AB731663	HQ385826	KU685583	MK272640	MN703131	MT194442
AB731665	HQ385830	KU685584	MK272641	MN703132	MT194443
AB731667	HQ385834	KU685585	MK272642	MN703133	MT194444
AB742145	HQ385835	KU685586	MK272643	MN703134	MT194445
AB746342	HQ385837	KU685587	MK272644	MN703135	MT194446
AB746344	HQ385838	KU685588	MK272645	MN703136	MT194447
AB773884	HQ385840	KU685589	MK272646	MN703137	MT194448
AB845344	HQ385841	KU685590	MK272647	MN703138	MT194449
AB845345	HQ385843	KU685591	MK272648	MN703139	MT194450
AB845346	HQ386140	KU685592	MK272649	MN703140	MT194451
AB845347	HQ386159	KU724103	MK272650	MN703141	MT194452
AB845348	HQ386173	KU724104	MK272651	MN703142	MT194453
AB845349	HQ386192	KU724105	MK272652	MN703143	MT194454
AB859012	HQ386210	KU749387	MK272653	MN703144	MT194455
AF003887	HQ540689	KU749394	MK272654	MN703145	MT194456
AF004394	HQ540690	KU749396	MK272655	MN703146	MT194457
AF004885	HQ540691	KU749399	MK272656	MN703147	MT194458
AF005494	HQ595742	KU749400	MK272657	MN703148	MT194459
AF005495	HQ595743	KU749401	MK272658	MN703149	MT194460
AF005496	HQ595744	KU749403	MK272659	MN703150	MT194461
AF009033	HQ595745	KU749404	MK272660	MN703151	MT194463
AF015916	HQ595746	KU749405	MK272661	MN703343	MT194464
AF015919	HQ595747	KU749407	MK272662	MN703344	MT194465
AF025749	HQ595748	KU749408	MK272663	MN703345	MT194466
AF025750	HQ595749	KU749409	MK272664	MN703346	MT194467
AF025751	HQ595750	KU749410	MK272665	MN703347	MT194468
AF025752	HQ595751	KU749411	MK272666	MN703348	MT194469
AF025753	HQ595752	KU749412	MK272667	MN703349	MT194470
AF025754	HQ595753	KU749413	MK272668	MN703350	MT194473
AF025755	HQ595755	KU749414	MK272669	MN703351	MT194475
AF025756	HQ595756	KU749415	MK272670	MN703352	MT194476
AF025757	HQ595757	KU749416	MK272671	MN703353	MT194477
AF025758	HQ595758	KU749417	MK272672	MN703354	MT194478
AF025759	HQ595759	KU749418	MK272673	MN703355	MT194479
AF025760	HQ595760	KU749419	MK272674	MN703356	MT194480
AF025761	HQ595761	KU749420	MK272675	MN703357	MT194481

AF025762	HQ595762	KU749421	MK272676	MN703358	MT194482
AF025763	HQ595765	KU749422	MK272677	MN703359	MT194483
AF025764	HQ595766	KU749423	MK272678	MN703360	MT194484
AF035532	HQ595767	KU749424	MK272679	MN703361	MT194485
AF041125	HQ595768	KU749425	MK272680	MN703362	MT194486
AF041126	HQ595769	KU749426	MK272681	MN703363	MT194487
AF041127	HQ595770	KU749428	MK272682	MN703364	MT194488
AF041128	HQ595771	KU749429	MK272683	MN703365	MT194489
AF041129	HQ595772	KU749430	MK272684	MN703366	MT194490
AF041130	HQ595773	KU749431	MK272685	MN703367	MT194491
AF041131	HQ595774	KU749432	MK272686	MN703368	MT194492
AF041132	HQ595775	KU820822	MK272687	MN703369	MT194493
AF041133	HQ595776	KU820823	MK272688	MN703370	MT194494
AF041134	HQ595777	KU820824	MK272689	MN703371	MT194495
AF041135	HQ595778	KU820825	MK272690	MN703372	MT194496
AF042100	HQ595779	KU820826	MK272691	MN703391	MT194497
AF042101	HQ595780	KU820827	MK272692	MN703392	MT194498
AF042102	HQ595781	KU820828	MK272693	MN703393	MT194499
AF042103	HQ595782	KU820829	MK272694	MN703394	MT194500
AF042104	HQ595783	KU820830	MK272695	MN703395	MT194502
AF042105	HQ595784	KU820831	MK272727	MN703396	MT194503
AF049337	HQ595785	KU820832	MK272728	MN736698	MT194504
AF049494	HQ595786	KU820833	MK272729	MN736699	MT194505
AF049495	HQ595787	KU820834	MK272730	MN736700	MT194506
AF061642	HQ595788	KU820835	MK272731	MN736701	MT194507
AF063224	HQ595789	KU820836	MK272732	MN736702	MT194508
AF064699	HQ595790	KU820837	MK272733	MN736703	MT194509
AF067154	HQ595791	KU820838	MK272734	MN736704	MT194510
AF067155	HQ595792	KU820839	MK272735	MN736705	MT194512
AF067156	HQ595793	KU820840	MK298150	MN736706	MT194513
AF067157	HQ595794	KU820841	MK303347	MN736707	MT194514
AF067158	HQ595795	KU820842	MK303348	MN736708	MT194515
AF067159	HQ595797	KU820843	MK303349	MN737451	MT194516
AF069139	HQ595798	KU820844	MK303350	MN737452	MT194517
AF069524	HQ595799	KU820845	MK303351	MN737453	MT194518
AF069669	HQ595800	KU820846	MK303352	MN752125	MT194519
AF069670	HQ595801	KU820847	MK303353	MN752126	MT194520
AF069671	HQ595802	KU820848	MK303354	MN752127	MT194521
AF069672	HQ595803	KU820849	MK303355	MN752128	MT194522
AF069673	HQ595804	KU820850	MK341078	MN752171	MT194524
AF069932	HQ595805	KU820851	MK371104	MN791130	MT194525
AF069933	HQ595806	KU820852	MK383384	MN791131	MT194526
AF069934	HQ595810	KU869532	MK383385	MN791132	MT194527
AF069935	HQ595978	KU869580	MK383386	MN791133	MT194528
AF069937	HQ596030	KU869582	MK383387	MN791134	MT194529
AF069939	HQ596137	KU869583	MK383388	MN791135	MT194530
AF069941	HQ615941	KU869584	MK383389	MN791136	MT194531

AF069943	HQ615942	KU869585	MK383390	MN791137	MT194532
AF069945	HQ615943	KU869586	MK383391	MN791138	MT194533
AF069947	HQ615944	KU869589	MK383392	MN791139	MT194534
AF070521	HQ615945	KU869595	MK383393	MN791140	MT194535
AF070703	HQ615946	KU869597	MK383394	MN791141	MT194536
AF070704	HQ615947	KU869602	MK383395	MN791142	MT194537
AF070705	HQ615948	KU869604	MK383396	MN791143	MT194538
AF070707	HQ615949	KU869607	MK383397	MN791144	MT194539
AF070708	HQ615950	KU886698	MK383398	MN791145	MT194540
AF070709	HQ615951	KU896101	MK383399	MN791146	MT194541
AF070710	HQ615952	KU896108	MK383400	MN791147	MT194542
AF070711	HQ615953	KU896115	MK383401	MN791148	MT194543
AF070713	HQ615955	KU896116	MK383402	MN791149	MT194544
AF071473	HQ615956	KU896118	MK383403	MN791151	MT194545
AF071474	HQ615957	KU896119	MK383404	MN791154	MT194546
AF075701	HQ615958	KU896123	MK383405	MN791158	MT194547
AF075702	HQ615959	KU896124	MK383406	MN791160	MT194548
AF075703	HQ615960	KU896125	MK383407	MN791162	MT194549
AF075720	HQ615961	KU901727	MK383408	MN791164	MT194550
AF076474	HQ615962	KU901757	MK383409	MN791165	MT194551
AF076475	HQ615963	KU901788	MK383410	MN791167	MT194552
AF076998	HQ615964	KU901807	MK383411	MN791168	MT194553
AF077336	HQ615966	KU901831	MK383412	MN791169	MT194554
AF082394	HQ615967	KU901856	MK383413	MN791170	MT194555
AF082395	HQ615968	KU901875	MK383414	MN791171	MT194556
AF082485	HQ615969	KU901898	MK383415	MN791172	MT194557
AF082486	HQ615970	KU921705	MK383416	MN791173	MT194558
AF084936	HQ615971	KU921708	MK383417	MN791174	MT194559
AF086817	HQ615972	KU921712	MK383418	MN791175	MT194560
AF107770	HQ615973	KU921715	MK383419	MN791176	MT194561
AF107771	HQ615974	KU921719	MK383420	MN791177	MT194562
AF110959	HQ615975	KU921722	MK383421	MN791178	MT194563
AF110962	HQ615976	KU921726	MK383422	MN791179	MT194564
AF110967	HQ615978	KU921730	MK383423	MN791180	MT194565
AF110969	HQ615979	KU921732	MK383424	MN791181	MT194566
AF110972	HQ615981	KU921734	MK383425	MN791182	MT194567
AF110973	HQ615982	KU921738	MK383426	MN791183	MT194568
AF110976	HQ615983	KU921740	MK383427	MN791184	MT194569
AF110979	HQ615984	KU921743	MK383428	MN791185	MT194570
AF119819	HQ616081	KU921745	MK383429	MN791186	MT194571
AF119820	HQ616082	KU921748	MK383430	MN791187	MT194572
AF128126	HQ616083	KU921749	MK383431	MN791188	MT194573
AF146728	HQ616085	KU921752	MK383432	MN791190	MT194574
AF164485	HQ616086	KU921755	MK383433	MN791192	MT194575
AF179368	HQ616088	KU921759	MK383434	MN791194	MT194576
AF184155	HQ616091	KU921765	MK383435	MN791196	MT194577
AF190127	HQ616093	KU921767	MK383436	MN791197	MT194578

AF190128	HQ616097	KU921768	MK383437	MN791198	MT194579
AF192135	HQ616100	KU921772	MK383438	MN791200	MT194580
AF193253	HQ625565	KU921776	MK383439	MN791202	MT194582
AF193275	HQ625566	KU921779	MK383440	MN791204	MT194583
AF193276	HQ625567	KU921781	MK383441	MN791206	MT194584
AF193277	HQ625568	KU921785	MK383442	MN791209	MT194585
AF193278	HQ625569	KU921788	MK383443	MN791210	MT194586
AF197340	HQ625570	KU921789	MK383444	MN791211	MT194587
AF197341	HQ625571	KU921793	MK383445	MN791212	MT194588
AF217150	HQ625572	KU921796	MK383446	MN791213	MT194590
AF219261	HQ625573	KU921798	MK383447	MN791214	MT194591
AF219262	HQ625574	KU921799	MK383448	MN791215	MT194594
AF219263	HQ625575	KU921802	MK383449	MN791216	MT194595
AF219264	HQ625576	KU921804	MK383450	MN791217	MT194596
AF219265	HQ625577	KU921805	MK383451	MN791218	MT194597
AF219266	HQ625578	KU921807	MK383452	MN791219	MT194598
AF219267	HQ625579	KU921810	MK383453	MN791220	MT194599
AF219268	HQ625580	KU921814	MK383454	MN791221	MT194600
AF219269	HQ625581	KU921823	MK383455	MN791222	MT194601
AF219270	HQ625582	KU921827	MK383456	MN791223	MT194602
AF219271	HQ625583	KU921831	MK383457	MN791224	MT194603
AF219272	HQ625584	KU921835	MK383458	MN791225	MT194604
AF219273	HQ625585	KU921838	MK383459	MN791226	MT194605
AF219274	HQ625586	KU921841	MK383460	MN791227	MT194606
AF219627	HQ625587	KU921852	MK383461	MN791228	MT194607
AF224507	HQ625588	KU992928	MK383462	MN791230	MT194608
AF227272	HQ625589	KU992929	MK383463	MN791233	MT194609
AF256204	HQ625590	KU992930	MK383464	MN791234	MT194610
AF259954	HQ625591	KU992931	MK383465	MN791237	MT194611
AF268277	HQ625592	KU992932	MK383466	MN791239	MT194612
AF277055	HQ625593	KU992934	MK383467	MN791242	MT194613
AF277056	HQ625594	KU992935	MK383468	MN791244	MT194614
AF277057	HQ625595	KU992936	MK383469	MN791246	MT194615
AF277058	HQ659584	KU992937	MK383470	MN791247	MT194616
AF277059	HQ659593	KX010453	MK383471	MN791248	MT194617
AF277061	HQ659612	KX027737	MK383472	MN791249	MT194618
AF277063	HQ659623	KX027965	MK383473	MN791250	MT194619
AF277064	HQ659632	KX028066	MK383474	MN791251	MT194620
AF277065	HQ690964	KX028188	MK383475	MN791252	MT194621
AF277066	HQ690973	KX028313	MK383476	MN791253	MT194622
AF277067	HQ691003	KX028499	MK383477	MN791254	MT194623
AF277068	HQ691004	KX028620	MK383478	MN791255	MT194624
AF277069	HQ691013	KX069219	MK383479	MN791256	MT194625
AF277070	HQ691020	KX069220	MK383480	MN791257	MT194626
AF277071	HQ691028	KX069221	MK383481	MN791258	MT194627
AF277072	HQ691039	KX069222	MK383482	MN791259	MT194628
AF277073	HQ691052	KX069223	MK383483	MN791260	MT194629

AF277074	HQ691073	KX069224	MK383484	MN791261	MT194630
AF277075	HQ691082	KX069225	MK383485	MN791262	MT194631
AF286223	HQ697934	KX069226	MK383486	MN791263	MT194632
AF286224	HQ697983	KX069227	MK383487	MN791264	MT194633
AF286225	HQ698029	KX069228	MK383488	MN791265	MT194634
AF286226	HQ698034	KX129208	MK383489	MN791266	MT194635
AF286227	HQ699949	KX129293	MK383490	MN791267	MT194636
AF286228	HQ699950	KX156365	MK383491	MN791268	MT194637
AF286229	HQ699951	KX156366	MK383492	MN791269	MT194638
AF286230	HQ699952	KX156371	MK383493	MN791270	MT194639
AF286231	HQ699953	KX156372	MK383494	MN791271	MT194640
AF286232	HQ699954	KX156374	MK383495	MN791272	MT194641
AF286233	HQ699955	KX156375	MK383496	MN791273	MT194642
AF286234	HQ699956	KX156387	MK383497	MN791274	MT194643
AF286235	HQ699957	KX156422	MK383498	MN791275	MT194644
AF286236	HQ699958	KX156436	MK383499	MN791276	MT194645
AF286237	HQ699959	KX156444	MK383500	MN791277	MT194646
AF286238	HQ699961	KX156482	MK383501	MN791278	MT194647
AF286239	HQ699962	KX156542	MK383502	MN791279	MT194648
AF286241	HQ699963	KX159285	MK383503	MN791280	MT194649
AF286365	HQ699964	KX168070	MK383504	MN791281	MT194650
AF289548	HQ699965	KX168085	MK383505	MN791282	MT194651
AF289549	HQ699966	KX181891	MK383506	MN791283	MT194652
AF289550	HQ699967	KX181894	MK383507	MN791284	MT194653
AF290027	HQ699968	KX181895	MK383508	MN791285	MT194654
AF316544	HQ699969	KX181896	MK383509	MN791286	MT194655
AF321079	HQ699970	KX181897	MK383510	MN791287	MT194656
AF321080	HQ699971	KX181898	MK383511	MN791288	MT194657
AF321081	HQ699972	KX181899	MK383512	MN791289	MT194658
AF321082	HQ699973	KX181900	MK383513	MN791290	MT194659
AF321083	HQ699974	KX181901	MK383514	MN791291	MT194660
AF321084	HQ699975	KX181902	MK383515	MN791292	MT194661
AF332867	HQ699976	KX181903	MK383516	MN791293	MT194662
AF361871	HQ699977	KX181904	MK383517	MN791294	MT194663
AF361872	HQ699978	KX181905	MK383518	MN791295	MT194664
AF361873	HQ699979	KX181906	MK383519	MN791296	MT194665
AF361874	HQ699980	KX181907	MK383520	MN791297	MT194666
AF361875	HQ699981	KX181908	MK383521	MN791298	MT194667
AF361876	HQ699982	KX181909	MK383522	MN791300	MT194668
AF361877	HQ699983	KX181910	MK383523	MN791301	MT194669
AF361878	HQ699984	KX181911	MK383524	MN791303	MT194670
AF361879	HQ699985	KX181912	MK383525	MN791304	MT194671
AF362994	HQ699986	KX181913	MK383526	MN791305	MT194672
AF377954	HQ699987	KX181914	MK383527	MN791308	MT194673
AF377955	HQ699988	KX181915	MK383528	MN791310	MT194674
AF377956	HQ699989	KX181916	MK383529	MN791312	MT194675
AF377957	HQ699990	KX181917	MK383530	MN791313	MT194676

AF377958	HQ699991	KX181918	MK383531	MN791314	MT194677
AF377959	HQ699992	KX181919	MK383532	MN791316	MT194679
AF383228	HQ699993	KX181920	MK383533	MN791319	MT194680
AF383229	HQ699994	KX181921	MK383534	MN791321	MT194681
AF383230	HQ699995	KX181922	MK383535	MN791322	MT194682
AF383231	HQ699996	KX181923	MK383536	MN791323	MT194683
AF383232	HQ699997	KX181924	MK383537	MN791325	MT194684
AF383233	HQ699998	KX181925	MK383538	MN791326	MT194685
AF383234	HQ699999	KX181926	MK383539	MN791328	MT194686
AF383235	HQ700000	KX181927	MK383540	MN791330	MT194687
AF383236	HQ700001	KX181928	MK383541	MN791331	MT194690
AF383237	HQ700002	KX181929	MK383542	MN791332	MT194691
AF383238	HQ700003	KX181930	MK383543	MN791333	MT194692
AF383240	HQ700004	KX181931	MK383544	MN791334	MT194693
AF383241	HQ834965	KX181932	MK383545	MN791335	MT194694
AF383242	HQ846900	KX181933	MK383546	MN791336	MT194695
AF383243	HQ896489	KX181934	MK383547	MN791337	MT194696
AF383244	HQ908109	KX181935	MK383548	MN791338	MT194697
AF383245	HQ908139	KX181936	MK383549	MN791339	MT194698
AF383246	HQ908150	KX181937	MK383550	MN791340	MT194699
AF383247	HQ908184	KX181938	MK383551	MN791341	MT194700
AF383248	HQ908219	KX181939	MK383552	MN791342	MT194701
AF383250	HQ908232	KX181940	MK383553	MN791344	MT194702
AF383251	HQ912706	KX181941	MK383554	MN791346	MT194703
AF383252	HQ912707	KX185086	MK383555	MN791348	MT194704
AF383253	HQ912708	KX216883	MK383556	MN791350	MT194705
AF383254	HQ912709	KX228799	MK383557	MN791351	MT194706
AF383255	HQ912710	KX228803	MK383558	MN791353	MT194707
AF383256	HQ912711	KX228807	MK383559	MN791355	MT194708
AF383257	J03653	KX228809	MK383560	MN791357	MT194709
AF383258	JF297221	KX228810	MK383561	MN791359	MT194710
AF383259	JF297222	KX228814	MK383562	MN791361	MT194711
AF383260	JF297225	KX228815	MK383563	MN791363	MT194712
AF383261	JF297229	KX228818	MK383564	MN791364	MT194713
AF383262	JF320002	KX228819	MK383565	MN791365	MT194714
AF385934	JF320007	KX228821	MK383566	MN791366	MT194715
AF385935	JF320008	KX228823	MK383567	MN791367	MT194716
AF385936	JF320011	KX228824	MK383568	MN791368	MT194717
AF391230	JF320012	KX228825	MK383569	MN791369	MT194718
AF391232	JF320017	KX232596	MK383570	MN791370	MT194719
AF391233	JF320018	KX232597	MK383571	MN791371	MT194720
AF391234	JF320028	KX232598	MK383572	MN791372	MT194721
AF391235	JF320036	KX232600	MK383573	MN791373	MT194722
AF391238	JF320038	KX232601	MK383574	MN791374	MT194723
AF391240	JF320043	KX232603	MK383575	MN791375	MT194724
AF391242	JF320044	KX232605	MK383576	MN791376	MT194725
AF391243	JF320045	KX232607	MK383577	MN791377	MT194726

AF391245	JF320048	KX232609	MK383578	MN791378	MT194727
AF391247	JF320054	KX232610	MK383579	MN791379	MT194728
AF391249	JF320059	KX232611	MK383580	MN791380	MT194729
AF391250	JF320072	KX232612	MK383581	MN791381	MT194730
AF407148	JF320083	KX232613	MK383582	MN791382	MT194731
AF407151	JF320099	KX232614	MK383583	MN791383	MT194732
AF407154	JF320120	KX232615	MK383584	MN791384	MT194733
AF407157	JF320131	KX232616	MK383585	MN791385	MT194734
AF407160	JF320144	KX232617	MK383586	MN791386	MT194735
AF407418	JF320145	KX232618	MK383587	MN791387	MT194736
AF408626	JF320150	KX232619	MK383588	MN791388	MT194737
AF408627	JF320160	KX232620	MK383589	MN791389	MT194738
AF408628	JF320173	KX232621	MK383590	MN791390	MT194739
AF408629	JF320179	KX232622	MK383592	MN791391	MT194740
AF408630	JF320183	KX232623	MK383593	MN791392	MT194741
AF408631	JF320184	KX232624	MK383594	MN791393	MT194742
AF408632	JF320185	KX232625	MK383595	MN791394	MT194743
AF411964	JF320186	KX232626	MK383596	MN791395	MT194744
AF411965	JF320189	KX232627	MK383597	MN791396	MT194745
AF411966	JF320191	KX232628	MK383598	MN791397	MT194746
AF411967	JF320196	KX232629	MK383599	MN791398	MT194747
AF413987	JF320197	KX353919	MK383600	MN791399	MT194748
AF414006	JF320198	KX364401	MK383601	MN791400	MT194749
AF423755	JF320230	KX378999	MK383602	MN791401	MT194750
AF423756	JF320241	KX379000	MK383603	MN791402	MT194751
AF423757	JF320259	KX389608	MK383604	MN791403	MT194752
AF423758	JF320276	KX389609	MK383605	MN791404	MT194753
AF423759	JF320296	KX389610	MK383606	MN791405	MT194754
AF423760	JF320308	KX389611	MK383607	MN791406	MT194755
AF442565	JF320315	KX389612	MK383608	MN791407	MT194756
AF442566	JF320329	KX389613	MK383609	MN791408	MT194757
AF442569	JF320356	KX389614	MK383610	MN791409	MT194758
AF442570	JF320361	KX389615	MK383611	MN791410	MT194759
AF443074	JF320363	KX389616	MK383612	MN791411	MT194760
AF443075	JF320375	KX389617	MK383613	MN791412	MT194761
AF443076	JF320380	KX389618	MK383614	MN791413	MT194762
AF443077	JF320387	KX389619	MK383615	MN791414	MT194763
AF443078	JF320394	KX389620	MK383616	MN791415	MT194764
AF443079	JF320411	KX389621	MK383617	MN791416	MT194765
AF443080	JF320422	KX389622	MK383618	MN791417	MT194766
AF443081	JF320427	KX389623	MK383619	MN791418	MT194767
AF443082	JF320460	KX389624	MK383620	MN791419	MT194768
AF443083	JF320482	KX389625	MK383621	MN791420	MT194770
AF443084	JF320497	KX389626	MK383622	MN791421	MT194771
AF443085	JF320520	KX389627	MK383623	MN791422	MT194772
AF443086	JF320530	KX389628	MK383624	MN791423	MT194774
AF443087	JF320539	KX389629	MK383625	MN791424	MT194775

AF443088	JF320559	KX389630	MK383626	MN791425	MT194776
AF443089	JF320564	KX389631	MK383627	MN791427	MT194777
AF443090	JF320570	KX389632	MK383628	MN791429	MT194778
AF443091	JF320592	KX389633	MK383629	MN791431	MT194779
AF443092	JF320613	KX389634	MK383630	MN791433	MT194781
AF443093	JF320615	KX389635	MK383631	MN791435	MT194782
AF443094	JF320630	KX389636	MK383632	MN791437	MT194783
AF443095	JF327807	KX389637	MK383633	MN791438	MT194784
AF443096	JF327808	KX389638	MK383634	MN791440	MT194785
AF443097	JF340054	KX389639	MK383635	MN791442	MT194788
AF443098	JF346900	KX389640	MK383636	MN791443	MT194789
AF443099	JF346901	KX389641	MK383637	MN791444	MT194790
AF443100	JF346902	KX389642	MK383638	MN791445	MT194792
AF443101	JF346903	KX389643	MK383639	MN791446	MT194793
AF443102	JF346904	KX389644	MK383640	MN791447	MT194796
AF443103	JF346905	KX389645	MK383641	MN791448	MT194797
AF443104	JF346906	KX389646	MK383642	MN791449	MT194798
AF443105	JF346907	KX389647	MK383643	MN791450	MT194799
AF443107	JF346908	KX389648	MK383644	MN791451	MT194800
AF443108	JF346909	KX389649	MK383645	MN791452	MT194801
AF443109	JF346910	KX398187	MK383646	MN791453	MT194802
AF443110	JF346911	KX434794	MK383647	MN791455	MT194803
AF443111	JF346912	KX434795	MK383648	MN791457	MT194804
AF443112	JF346913	KX434796	MK383649	MN791460	MT194805
AF443113	JF346914	KX434797	MK383650	MN791462	MT194806
AF443114	JF346915	KX434798	MK383651	MN791464	MT194807
AF443115	JF346916	KX434799	MK383652	MN791467	MT194808
AF450096	JF346917	KX505395	MK383653	MN791469	MT194809
AF450097	JF346918	KX505412	MK383654	MN791471	MT194810
AF450098	JF346919	KX505435	MK383655	MN791472	MT194811
AF457051	JF421359	KX505441	MK383656	MN791474	MT194812
AF457052	JF421360	KX505460	MK383657	MN791476	MT194813
AF457053	JF421361	KX505504	MK383658	MN791478	MT194814
AF457054	JF421362	KX505535	MK383659	MN791479	MT194816
AF457055	JF421363	KX505552	MK383660	MN791481	MT194818
AF457056	JF421364	KX505583	MK383661	MN791483	MT194819
AF457058	JF421365	KX505602	MK383662	MN791485	MT194820
AF457059	JF421366	KX505643	MK383663	MN791487	MT194821
AF457061	JF421367	KX505649	MK383664	MN791489	MT194822
AF457062	JF421368	KX505664	MK383665	MN791492	MT194823
AF457063	JF421369	KX505695	MK383666	MN791493	MT194824
AF457064	JF421370	KX505707	MK383667	MN791494	MT194825
AF457065	JF421371	KX505734	MK383668	MN791495	MT194826
AF457066	JF421372	KX579838	MK383669	MN791496	MT194827
AF457067	JF421373	KX582249	MK383670	MN791497	MT194828
AF457068	JF421374	KX582250	MK383671	MN791498	MT194829
AF457069	JF421375	KX582251	MK383672	MN791499	MT194830

AF457070	JF421376	KX587007	MK383673	MN791500	MT194831
AF457072	JF421377	KX587035	MK383674	MN791501	MT194832
AF457073	JF421378	KX587074	MK383675	MN791502	MT194833
AF457075	JF421379	KX587114	MK383676	MN791503	MT194834
AF457077	JF421380	KX587162	MK383677	MN791505	MT194835
AF457078	JF421381	KX587201	MK383678	MN791507	MT194836
AF457079	JF421382	KX587239	MK383679	MN791509	MT194837
AF457080	JF421383	KX587277	MK383680	MN791510	MT194838
AF457081	JF421384	KX587296	MK383681	MN791511	MT194839
AF457082	JF421385	KX587339	MK383682	MN791513	MT194840
AF457083	JF421386	KX587376	MK383683	MN791515	MT194841
AF457084	JF421387	KX587433	MK383684	MN791517	MT194842
AF457085	JF421388	KX587448	MK383685	MN791520	MT194843
AF457086	JF421389	KX595119	MK383686	MN791522	MT194844
AF457087	JF421390	KX673818	MK383687	MN791524	MT194845
AF457088	JF421391	KX673819	MK383690	MN791525	MT194846
AF457089	JF421392	KX673820	MK383695	MN791526	MT194847
AF457090	JF421393	KX673821	MK383698	MN791527	MT194848
AF457101	JF421394	KX692903	MK383699	MN791528	MT194849
AF460972	JF421395	KX692925	MK383700	MN791529	MT194850
AF460974	JF421414	KX692941	MK383701	MN791530	MT194851
AF468970	JF421415	KX692948	MK383706	MN791531	MT194852
AF484477	JF421416	KX692969	MK383709	MN791532	MT194853
AF484478	JF421417	KX692980	MK383711	MN791533	MT194854
AF484479	JF421418	KX692997	MK383713	MN791535	MT194855
AF484480	JF421419	KX693025	MK383716	MN791537	MT194856
AF484481	JF421420	KX693036	MK383720	MN791539	MT194857
AF484482	JF421421	KX693054	MK383721	MN791541	MT194858
AF484483	JF680905	KX693065	MK383725	MN791543	MT194859
AF484485	JF680906	KX693075	MK383728	MN791545	MT194860
AF484486	JF680907	KX693092	MK383736	MN791547	MT194861
AF484487	JF680908	KX693099	MK383737	MN791549	MT194862
AF484488	JF680909	KX693100	MK383738	MN791551	MT194863
AF484489	JF680910	KX693113	MK383744	MN791553	MT194864
AF484490	JF680911	KX693131	MK383747	MN791555	MT194865
AF484491	JF680912	KX693150	MK383749	MN791557	MT194866
AF484492	JF680913	KX693166	MK383753	MN791559	MT194867
AF484493	JF680914	KX693171	MK383755	MN791561	MT194868
AF484494	JF680915	KX693190	MK383757	MN791562	MT194869
AF484495	JF680916	KX693215	MK383758	MN791564	MT194870
AF484496	JF680917	KX693238	MK383759	MN791567	MT194871
AF484497	JF680918	KX693247	MK383762	MN791569	MT194872
AF484498	JF680919	KX693265	MK383765	MN791572	MT194873
AF484499	JF680920	KX693281	MK383766	MN791573	MT194874
AF484500	JF680921	KX693301	MK383770	MN791575	MT194875
AF484501	JF680922	KX693308	MK383771	MN791577	MT194876
AF484502	JF680923	KX693314	MK383772	MN791579	MT194877

AF484503	JF680924	KX693322	MK383775	MN791580	MT194878
AF484504	JF680925	KX693338	MK383776	MN791582	MT194879
AF484505	JF680926	KX693357	MK383777	MN791584	MT194880
AF484506	JF680927	KX693366	MK383780	MN791585	MT194881
AF484507	JF680928	KX693387	MK383782	MN791586	MT194882
AF484508	JF680929	KX693393	MK383783	MN791587	MT194883
AF484509	JF680930	KX693415	MK383784	MN791588	MT194884
AF484510	JF680931	KX693430	MK383790	MN791589	MT194885
AF484511	JF680932	KX693454	MK383801	MN791590	MT194886
AF484512	JF680933	KX693468	MK383804	MN791591	MT194887
AF484513	JF680934	KX693475	MK383808	MN791592	MT194888
AF484514	JF680935	KX693489	MK383814	MN791593	MT194889
AF484515	JF680936	KX693491	MK383815	MN791594	MT194890
AF484516	JF680937	KX693496	MK383816	MN791595	MT194891
AF484517	JF683736	KX693515	MK383819	MN791596	MT194892
AF484518	JF683737	KX693533	MK383821	MN791597	MT194893
AF484519	JF683738	KX702368	MK383825	MN791598	MT194894
AF484520	JF683739	KX756600	MK383827	MN791599	MT194895
AF484521	JF683740	KX756601	MK383834	MN791600	MT194896
AF484522	JF683741	KX756602	MK383835	MN791601	MT194897
AF490512	JF683742	KX756603	MK383837	MN791602	MT194898
AF490513	JF683743	KX756604	MK383839	MN791603	MT194899
AF490973	JF683744	KX756605	MK383848	MN791604	MT194900
AF490974	JF683745	KX756606	MK383849	MN791605	MT194901
AF491737	JF683746	KX756607	MK383850	MN791606	MT194902
AF491740	JF683747	KX756608	MK383851	MN791607	MT194903
AF492623	JF683748	KX756609	MK383855	MN791608	MT194904
AF492624	JF683749	KX756610	MK383857	MN791609	MT194906
AF503396	JF683750	KX756611	MK383858	MN791612	MT194907
AF516184	JF683751	KX756612	MK383860	MN791614	MT194908
AF529572	JF683752	KX756613	MK383864	MN791616	MT194910
AF529573	JF683753	KX756614	MK383865	MN791617	MT194911
AF530576	JF683754	KX756615	MK383866	MN791619	MT194912
AF538302	JF683755	KX792557	MK383867	MN791621	MT194913
AF538303	JF683756	KX792565	MK383874	MN791622	MT194915
AF538304	JF683757	KX792587	MK383875	MN791623	MT194916
AF538305	JF683758	KX792606	MK383879	MN791626	MT194917
AF538306	JF683759	KX792634	MK383883	MN791627	MT194918
AF538307	JF683760	KX792657	MK383887	MN791629	MT194920
AF539404	JF683761	KX792677	MK383889	MN791630	MT194921
AF544007	JF683763	KX792688	MK383891	MN791631	MT194922
AF544008	JF683764	KX792706	MK383894	MN791632	MT194923
AJ006022	JF683765	KX792725	MK383896	MN791633	MT194924
AJ237565	JF683766	KX792750	MK383898	MN791634	MT194925
AJ239083	JF683767	KX792768	MK383900	MN791635	MT194926
AJ245481	JF683768	KX792798	MK383902	MN791636	MT194927
AJ249235	JF683769	KX792808	MK383904	MN791637	MT194928

AJ249236	JF683770	KX792813	MK383908	MN791638	MT194929
AJ249237	JF683771	KX792831	MK383909	MN791639	MT194930
AJ249238	JF683772	KX792852	MK383910	MN791640	MT194931
AJ249239	JF683773	KX818199	MK383911	MN791641	MT194932
AJ251056	JF683774	KX818200	MK383915	MN791642	MT194933
AJ251057	JF683775	KX907336	MK383916	MN791643	MT194934
AJ271111	JF683776	KX907338	MK383917	MN791644	MT194935
AJ271370	JF683777	KX907342	MK383919	MN791645	MT194936
AJ271445	JF683778	KX907343	MK383920	MN791646	MT194937
AJ276221	JF683779	KX907344	MK383923	MN791648	MT194938
AJ276595	JF683780	KX907346	MK383926	MN791649	MT194939
AJ276596	JF683781	KX907348	MK383927	MN791650	MT194940
AJ277818	JF683782	KX907349	MK383934	MN791652	MT194941
AJ277819	JF683783	KX907351	MK383936	MN791653	MT194942
AJ277820	JF683784	KX907353	MK383938	MN791654	MT194943
AJ277821	JF683785	KX907354	MK383940	MN791655	MT194944
AJ277822	JF683786	KX907360	MK383942	MN791656	MT194945
AJ277823	JF683787	KX907361	MK383945	MN791657	MT194946
AJ277824	JF683788	KX907362	MK383946	MN791658	MT194947
AJ277825	JF683789	KX907364	MK383947	MN791660	MT194948
AJ286133	JF683790	KX907367	MK383950	MN791661	MT194949
AJ286330	JF683791	KX907368	MK383951	MN791662	MT194950
AJ286332	JF683792	KX907375	MK383954	MN791663	MT194951
AJ286334	JF683793	KX907377	MK383955	MN791664	MT194952
AJ286335	JF683794	KX907381	MK383957	MN791666	MT194953
AJ286337	JF683795	KX907382	MK383958	MN791667	MT194954
AJ286339	JF683796	KX907385	MK383963	MN791668	MT194955
AJ286341	JF683797	KX907389	MK383965	MN791669	MT194956
AJ288981	JF683798	KX907390	MK383967	MN791670	MT194959
AJ288982	JF683799	KX907391	MK383971	MN791671	MT194960
AJ291718	JF683800	KX907392	MK383972	MN791672	MT194961
AJ291719	JF683801	KX907394	MK383973	MN791673	MT194962
AJ291720	JF683802	KX907398	MK383976	MN791674	MT194963
AJ293865	JF683803	KX907399	MK383979	MN791675	MT194964
AJ302646	JF683804	KX907401	MK383984	MN791676	MT194965
AJ302647	JF683805	KX907402	MK383993	MN791677	MT194966
AJ401034	JF683806	KX907411	MK384001	MN791678	MT194968
AJ401038	JF683807	KX907414	MK384002	MN791679	MT194969
AJ401039	JF683808	KX907416	MK384007	MN791680	MT194970
AJ401040	JF683809	KX907418	MK384011	MN791681	MT194971
AJ401043	JF689852	KX907419	MK384012	MN791682	MT194972
AJ401044	JF689854	KX907422	MK384013	MN791683	MT194973
AJ404325	JF689856	KX907423	MK384017	MN791684	MT194974
AJ417408	JF689857	KX907425	MK384018	MN791685	MT194975
AJ417411	JF689859	KX907426	MK384022	MN791686	MT194976
AJ417414	JF689860	KX907427	MK384023	MN791687	MT194978
AJ417417	JF689862	KX907428	MK384026	MN791688	MT194979

AJ417420	JF689863	KX907429	MK384027	MN791690	MT194980
AJ417423	JF689864	KX907431	MK384030	MN791693	MT194981
AJ417426	JF689865	KX907432	MK384033	MN791695	MT194982
AJ417429	JF689866	KX960962	MK384034	MN791699	MT194983
AJ418478	JF689867	KX960963	MK384035	MN791701	MT194984
AJ418495	JF689868	KX960964	MK384037	MN791703	MT194985
AJ418514	JF689870	KX960965	MK384038	MN791705	MT194986
AJ418521	JF689871	KX960966	MK384040	MN791706	MT194987
AJ418531	JF689872	KX960967	MK384042	MN791707	MT194988
AJ488926	JF689873	KX960968	MK384045	MN791708	MT194989
AJ488927	JF689874	KX960969	MK384046	MN791709	MT194990
AJ508595	JF689875	KX960970	MK384048	MN791710	MT194991
AJ508596	JF689876	KX960971	MK384050	MN791711	MT194992
AJ508597	JF689877	KX960972	MK384055	MN791712	MT194993
AJ535590	JF689879	KX960973	MK384062	MN791713	MT194994
AJ535600	JF689883	KX960974	MK384064	MN791714	MT194995
AJ535608	JF689884	KX983542	MK384066	MN791715	MT194996
AJ535612	JF689885	KX983566	MK384067	MN791716	MT194997
AJ535616	JF689886	KX983624	MK384071	MN791717	MT194999
AJ866553	JF689887	KX983643	MK384076	MN791719	MT195000
AJ866554	JF689889	KX983685	MK384077	MN791720	MT195001
AJ866555	JF689890	KX983732	MK384078	MN791722	MT195002
AJ866556	JF689892	KX983765	MK384079	MN791723	MT195003
AJ866557	JF689893	KX983797	MK384081	MN791725	MT195004
AJ866558	JF689894	KX983831	MK384085	MN791726	MT195005
AM000054	JF689895	KX983851	MK384086	MN791729	MT195006
AM000055	JF689896	KX983891	MK384089	MN791732	MT195007
AM279343	JF689897	KX984367	MK384091	MN791735	MT195008
AM279344	JF706370	KX984437	MK384095	MN791737	MT195009
AM279345	JF706405	KX984523	MK384099	MN791738	MT195010
AM279346	JF706428	KX984588	MK384110	MN791740	MT195011
AM279347	JF706448	KX984665	MK384115	MN791742	MT195012
AM279348	JF706472	KX984753	MK384116	MN791744	MT195013
AM279349	JF719819	KX984836	MK384117	MN791746	MT195014
AM279350	JF804805	KX984877	MK384118	MN791748	MT195015
AM279351	JF804806	KX984902	MK384122	MN791750	MT195016
AM279352	JF804807	KY019257	MK384123	MN791752	MT195017
AM279353	JF804808	KY057382	MK384124	MN791753	MT195018
AM279354	JF804809	KY057496	MK384128	MN791755	MT195019
AM279355	JF804810	KY057570	MK384129	MN791757	MT195020
AM279356	JF804811	KY057587	MK384130	MN791759	MT195021
AM279357	JF804812	KY111965	MK384131	MN791761	MT195022
AM279358	JF804813	KY111987	MK384132	MN791763	MT195023
AM279359	JF804814	KY112197	MK384138	MN791765	MT195024
AM279360	JF932468	KY112259	MK384140	MN791767	MT195025
AM279361	JF932469	KY112360	MK384143	MN791768	MT195026
AM279362	JF932470	KY112522	MK384144	MN791769	MT195027

AM279363	JF932471	KY113379	MK384145	MN791771	MT195028
AM279364	JF932472	KY113566	MK384146	MN791773	MT195029
AM279365	JF932473	KY113735	MK384149	MN791775	MT195030
AM279366	JF932474	KY113882	MK384157	MN791779	MT195031
AM279367	JF932475	KY200513	MK384161	MN791780	MT195032
AM851090	JF932476	KY200514	MK384165	MN791781	MT195033
AM851091	JF932477	KY200515	MK384167	MN791782	MT195034
AP005206	JF932478	KY200516	MK384168	MN791783	MT195035
AP005207	JF932479	KY200517	MK384169	MN791784	MT195036
AY008714	JF932480	KY203346	MK384170	MN791785	MT195037
AY008715	JF932481	KY213715	MK384175	MN791786	MT195039
AY008716	JF932482	KY213716	MK384186	MN791787	MT195040
AY008717	JF932483	KY213717	MK384191	MN791788	MT195041
AY008718	JF932484	KY213718	MK384196	MN791789	MT195042
AY037266	JF932485	KY213719	MK384199	MN791790	MT195043
AY037267	JF932486	KY213720	MK384200	MN791791	MT195044
AY037268	JF932487	KY213721	MK384201	MN791792	MT195045
AY037269	JF932488	KY213722	MK384206	MN791793	MT195046
AY037270	JF932489	KY213723	MK384207	MN791794	MT195047
AY037271	JF932490	KY213724	MK384208	MN791795	MT195048
AY037272	JF932491	KY213725	MK384209	MN791796	MT195049
AY037275	JF932492	KY213726	MK384210	MN791797	MT195050
AY037277	JF932493	KY213727	MK384212	MN791798	MT195051
AY037278	JF932494	KY213728	MK384215	MN791799	MT195052
AY037280	JF932495	KY213729	MK384217	MN791800	MT195053
AY037281	JF932496	KY213730	MK384219	MN791801	MT195054
AY037282	JF932497	KY213731	MK384220	MN791802	MT195055
AY037283	JF932498	KY213732	MK384223	MN791803	MT195056
AY037285	JF932499	KY213733	MK384224	MN791804	MT195057
AY043174	JF932500	KY213734	MK384226	MN791805	MT195058
AY043175	JF952012	KY213735	MK384231	MN791806	MT195059
AY043176	JF952013	KY213736	MK384237	MN791807	MT195060
AY046058	JF952014	KY213737	MK384241	MN791809	MT195061
AY049708	JF952015	KY213738	MK384242	MN791812	MT195062
AY074891	JF952016	KY213739	MK384254	MN791814	MT195063
AY082968	JF952017	KY213740	MK384259	MN791816	MT195064
AY093603	JF952018	KY213741	MK384261	MN791818	MT195065
AY093604	JN001990	KY213742	MK384265	MN791819	MT195066
AY093605	JN002008	KY213743	MK384268	MN791820	MT195067
AY093607	JN024100	KY213744	MK384269	MN791822	MT195068
AY118165	JN024203	KY213745	MK384272	MN791825	MT195069
AY118166	JN024274	KY213746	MK384273	MN791827	MT195070
AY125894	JN024339	KY213747	MK384276	MN791829	MT195071
AY151001	JN024349	KY213748	MK384277	MN791833	MT195072
AY151002	JN024450	KY213749	MK384278	MN791835	MT195073
AY158533	JN024459	KY213751	MK384279	MN791837	MT195074
AY158534	JN024469	KY216146	MK384280	MN791839	MT195075

AY158535	JN029801	KY216147	MK384286	MN791841	MT195076
AY167123	JN029803	KY216148	MK384287	MN791843	MT195077
AY173951	JN034137	KY229251	MK384292	MN791845	MT195078
AY173952	JN034158	KY229257	MK384295	MN791847	MT195079
AY173954	JN054257	KY229265	MK384296	MN791849	MT195080
AY173955	JN054260	KY229291	MK384297	MN791850	MT195081
AY173957	JN054263	KY229314	MK384301	MN791853	MT195082
AY173959	JN054265	KY229316	MK384302	MN791854	MT195083
AY173960	JN054269	KY229337	MK384306	MN791855	MT195084
AY180905	JN054273	KY229347	MK384308	MN791856	MT195085
AY189526	JN054274	KY229356	MK384310	MN791857	MT195086
AY217545	JN054279	KY229379	MK384311	MN791858	MT195087
AY217546	JN054280	KY229391	MK384312	MN791859	MT195088
AY223743	JN054285	KY229397	MK384313	MN791860	MT195089
AY223761	JN054287	KY229414	MK384317	MN791862	MT195090
AY227107	JN054289	KY229423	MK384324	MN791863	MT195091
AY228556	JN054296	KY229434	MK384327	MN791864	MT195092
AY228557	JN054300	KY229450	MK384328	MN791865	MT195093
AY231152	JN106043	KY229460	MK384329	MN791866	MT195094
AY231153	JN188292	KY229472	MK384331	MN791867	MT195095
AY231154	JN230353	KY229484	MK384334	MN791868	MT195096
AY231155	JN235952	KY229510	MK384337	MN791869	MT195097
AY231156	JN235953	KY229518	MK384339	MN791870	MT195098
AY231157	JN235956	KY229519	MK384342	MN791871	MT195099
AY231158	JN235957	KY229533	MK384344	MN791872	MT195100
AY247218	JN235958	KY229542	MK384346	MN791873	MT195101
AY247219	JN235959	KY229554	MK384347	MN791874	MT195102
AY247220	JN235960	KY229565	MK384350	MN791875	MT195103
AY247221	JN235962	KY229617	MK384358	MN791876	MT195104
AY247222	JN235963	KY229633	MK384361	MN791877	MT195105
AY247223	JN235964	KY229639	MK384364	MN791878	MT195106
AY247224	JN235965	KY229650	MK384372	MN791879	MT195107
AY247225	JN248316	KY229661	MK384373	MN791880	MT195108
AY253303	JN248317	KY229671	MK384374	MN791881	MT195109
AY253304	JN248318	KY229675	MK384379	MN791882	MT195110
AY253305	JN248319	KY275364	MK384382	MN791883	MT195111
AY253307	JN248320	KY323724	MK384385	MN791884	MT195112
AY253308	JN248321	KY323813	MK384387	MN791885	MT195113
AY253309	JN248322	KY323883	MK384390	MN791886	MT195114
AY253310	JN248323	KY323997	MK384395	MN791887	MT195115
AY253311	JN248324	KY324081	MK384397	MN791888	MT195116
AY253312	JN248325	KY324166	MK384398	MN791889	MT195117
AY253313	JN248326	KY324257	MK384399	MN791890	MT195118
AY253314	JN248327	KY324331	MK384400	MN791891	MT195119
AY253315	JN248328	KY324398	MK384403	MN791892	MT195120
AY253316	JN248329	KY324503	MK384404	MN791893	MT195121
AY253317	JN248330	KY324517	MK384407	MN791894	MT195122

AY253318	JN248331	KY324593	MK384410	MN791895	MT195123
AY253319	JN248332	KY324640	MK384412	MN791896	MT195124
AY253320	JN248333	KY324735	MK384413	MN791897	MT195125
AY253321	JN248334	KY324805	MK384415	MN791898	MT195126
AY253322	JN248335	KY345446	MK384421	MN791899	MT195127
AY255823	JN248336	KY345628	MK384425	MN791900	MT195128
AY255824	JN248337	KY345688	MK384426	MN791901	MT195129
AY255825	JN248338	KY345745	MK384429	MN791902	MT195130
AY255826	JN248339	KY345851	MK384430	MN791903	MT195131
AY262830	JN248340	KY345865	MK384433	MN791904	MT195132
AY271690	JN248341	KY345973	MK384434	MN791905	MT195133
AY304496	JN248342	KY346080	MK384436	MN791906	MT195134
AY308760	JN248343	KY346118	MK384446	MN791907	MT195135
AY314044	JN248344	KY346148	MK384447	MN791908	MT195136
AY322184	JN248345	KY346269	MK384450	MN791909	MT195137
AY322187	JN248346	KY346293	MK384456	MN791910	MT195138
AY322189	JN248347	KY346324	MK384457	MN791911	MT195139
AY322190	JN248348	KY346355	MK384461	MN791912	MT195140
AY322191	JN248349	KY346382	MK384463	MN791913	MT195141
AY322193	JN248350	KY346432	MK384465	MN791914	MT195142
AY331282	JN248351	KY346462	MK384467	MN791915	MT195143
AY331284	JN248352	KY359380	MK384470	MN791916	MT195144
AY331285	JN248353	KY359381	MK384473	MN791917	MT195145
AY331287	JN248354	KY359382	MK384476	MN791918	MT195146
AY331289	JN248355	KY359383	MK384477	MN791919	MT195147
AY331291	JN248356	KY359384	MK384478	MN791920	MT195148
AY331292	JN248357	KY359385	MK384480	MN791921	MT195149
AY331294	JN248580	KY392767	MK384481	MN791923	MT195150
AY331296	JN248582	KY392768	MK384483	MN791924	MT195151
AY352275	JN248584	KY392769	MK384484	MN791925	MT195152
AY352654	JN248585	KY392770	MK384490	MN791926	MT195153
AY352655	JN248586	KY392771	MK384493	MN791927	MT195154
AY352656	JN248587	KY392772	MK384495	MN791928	MT195155
AY352657	JN248589	KY392773	MK384496	MN791929	MT195156
AY357338	JN248590	KY392774	MK384499	MN791930	MT195157
AY358036	JN248591	KY392775	MK384500	MN791931	MT195158
AY358037	JN248592	KY392776	MK384501	MN791932	MT195159
AY358038	JN248593	KY392777	MK384502	MN791933	MT195160
AY358039	JN251812	KY392778	MK384504	MN791934	MT195161
AY358040	JN251896	KY392779	MK384509	MN791935	MT195162
AY358041	JN251897	KY392780	MK384514	MN791936	MT195163
AY358042	JN251898	KY406739	MK384516	MN791937	MT195164
AY358043	JN251899	KY465967	MK384521	MN791938	MT195165
AY358044	JN251900	KY465968	MK384522	MN791939	MT195166
AY358045	JN251901	KY465969	MK384526	MN791940	MT195167
AY358046	JN251902	KY496622	MK384528	MN791941	MT195168
AY358047	JN251903	KY496623	MK384529	MN791942	MT195169

AY358048	JN251905	KY496624	MK384532	MN791943	MT195170
AY358049	JN251906	KY498771	MK384535	MN791944	MT195171
AY358050	JN388081	KY514084	MK384538	MN791945	MT195172
AY358051	JN388091	KY514085	MK384541	MN791946	MT195173
AY358052	JN388101	KY557336	MK384542	MN791947	MT195174
AY358056	JN388111	KY580473	MK384543	MN791948	MT195175
AY358057	JN388121	KY580512	MK384544	MN791949	MT195176
AY358059	JN388131	KY580643	MK384546	MN791950	MT195177
AY358060	JN388141	KY612718	MK384547	MN791951	MT195178
AY358061	JN388151	KY628216	MK384548	MN791952	MT195179
AY358062	JN388161	KY628218	MK384549	MN791953	MT195180
AY358063	JN388171	KY628219	MK384550	MN791954	MT195181
AY358064	JN388181	KY628221	MK384554	MN791955	MT195182
AY358065	JN388191	KY628223	MK384559	MN791956	MT195183
AY358066	JN388201	KY628225	MK384561	MN791957	MT195184
AY358067	JN388211	KY658681	MK384563	MN791958	MT195186
AY358068	JN388221	KY658682	MK384565	MN791959	MT195187
AY358069	JN397362	KY658683	MK384566	MN791960	MT195188
AY358070	JN397363	KY658684	MK384570	MN791961	MT195189
AY358071	JN397364	KY658685	MK384571	MN791962	MT195191
AY358072	JN397365	KY658686	MK384573	MN791963	MT195192
AY358073	JN417236	KY658687	MK384579	MN791964	MT195193
AY371121	JN417237	KY658688	MK384583	MN791965	MT195194
AY371122	JN417238	KY658689	MK384589	MN791966	MT195195
AY371123	JN417239	KY658690	MK384592	MN791967	MT195196
AY371124	JN417240	KY658691	MK384596	MN791968	MT195197
AY371125	JN417241	KY658692	MK384602	MN791969	MT195198
AY371126	JN562755	KY658693	MK384604	MN791970	MT195199
AY371127	JN562759	KY658694	MK384607	MN791971	MT195200
AY371128	JN562764	KY658695	MK384608	MN791972	MT195201
AY371129	JN562770	KY658696	MK384615	MN791973	MT195202
AY371130	JN562775	KY658698	MK384616	MN791974	MT195203
AY371131	JN562779	KY658699	MK384617	MN791975	MT195204
AY371132	JN562785	KY658700	MK384620	MN791976	MT195205
AY371134	JN562791	KY658701	MK384623	MN791977	MT195206
AY371136	JN562797	KY658702	MK384624	MN791978	MT195207
AY371137	JN562802	KY658703	MK384625	MN791979	MT195208
AY371138	JN571034	KY658705	MK384628	MN791980	MT195209
AY371139	JN572926	KY658706	MK384629	MN791981	MT195210
AY371140	JN599164	KY713228	MK384631	MN791982	MT195211
AY371141	JN631793	KY713229	MK384632	MN791983	MT195212
AY371142	JN681219	KY713230	MK384633	MN791984	MT195213
AY371143	JN681220	KY713231	MK384637	MN791985	MT195214
AY371145	JN681221	KY713232	MK384638	MN791986	MT195215
AY371146	JN681222	KY713233	MK384640	MN791987	MT195216
AY371147	JN681227	KY713234	MK384641	MN791988	MT195217
AY371149	JN681229	KY713235	MK384643	MN791989	MT195218

AY371150	JN681232	KY713236	MK384644	MN791990	MT195219
AY371151	JN681233	KY713248	MK384648	MN791991	MT195220
AY371153	JN681234	KY748419	MK384650	MN791992	MT195221
AY371154	JN681235	KY748513	MK384653	MN791993	MT195222
AY371155	JN681236	KY766150	MK384654	MN791995	MT195223
AY371156	JN681237	KY778287	MK384655	MN791997	MT195224
AY371157	JN681238	KY778336	MK384656	MN791999	MT195225
AY371158	JN681239	KY778387	MK384658	MN792001	MT195226
AY371159	JN681240	KY778451	MK384660	MN792004	MT195227
AY371160	JN681241	KY778593	MK384663	MN792006	MT195228
AY371161	JN681243	KY929360	MK384667	MN792008	MT195229
AY371162	JN681244	KY929361	MK384669	MN792010	MT195230
AY371163	JN681245	KY929362	MK384671	MN792012	MT195231
AY371164	JN681246	KY929363	MK384674	MN792013	MT195232
AY371165	JN681247	KY929364	MK384675	MN792014	MT195233
AY371166	JN681248	KY929365	MK384676	MN792015	MT195234
AY371167	JN681249	KY929366	MK384678	MN792016	MT195235
AY371168	JN681250	KY929367	MK384680	MN792017	MT195236
AY371169	JN681251	KY929368	MK384682	MN792018	MT195237
AY371170	JN681252	KY929369	MK384683	MN792019	MT195238
AY423381	JN681253	KY929370	MK384685	MN792020	MT195239
AY423971	JN681254	KY950610	MK384687	MN792021	MT195240
AY423984	JN681255	KY953197	MK384689	MN792022	MT195241
AY424079	JN681256	KY953198	MK384690	MN792023	MT195242
AY424138	JN681257	KY953200	MK384693	MN792024	MT195243
AY424163	JN681258	KY953201	MK384695	MN792025	MT195244
AY426125	JN681259	KY953202	MK384698	MN792026	MT195245
AY444799	JN687694	KY968394	MK384703	MN792027	MT195246
AY444800	JN687704	KY968395	MK384704	MN792028	MT195247
AY444801	JN687706	KY968396	MK384705	MN792029	MT195248
AY444803	JN687717	KY968397	MK384710	MN792030	MT195249
AY444804	JN687718	KY968398	MK384711	MN792031	MT195250
AY444805	JN687726	KY968399	MK384714	MN792032	MT195251
AY444806	JN687728	KY968400	MK384716	MN792033	MT195252
AY444809	JN687730	KY968401	MK384717	MN792035	MT195253
AY444811	JN687731	KY968402	MK384718	MN792037	MT195254
AY444812	JN687734	KY968403	MK384719	MN792039	MT195255
AY455778	JN687735	KY968404	MK384720	MN792041	MT195256
AY455779	JN687736	KY968405	MK384728	MN792043	MT195257
AY455780	JN687737	KY968407	MK384729	MN792045	MT195258
AY455781	JN687738	KY968408	MK384734	MN792047	MT195259
AY455782	JN687739	KY989949	MK384735	MN792048	MT195260
AY455783	JN687742	KY989950	MK384736	MN792051	MT195261
AY455784	JN687744	KY989951	MK384739	MN792053	MT195262
AY455785	JN687746	KY989952	MK384740	MN792055	MT195263
AY463217	JN687748	KY989953	MK384743	MN792056	MT195264
AY463218	JN687749	KY989954	MK384744	MN792057	MT195265

AY463219	JN687750	KY989955	MK384745	MN792058	MT195266
AY463220	JN687758	KY989956	MK384746	MN792059	MT195267
AY463221	JN687759	KY989957	MK384748	MN792060	MT195268
AY463222	JN687760	KY995542	MK384749	MN792061	MT195269
AY463223	JN687761	L02317	MK384750	MN792062	MT195270
AY463224	JN687762	L07082	MK384751	MN792063	MT195271
AY463225	JN687763	L07421	MK384754	MN792064	MT195272
AY463226	JN687773	L08655	MK384760	MN792065	MT195273
AY463227	JN687774	L20571	MK384763	MN792066	MT195274
AY463228	JN687775	L20587	MK384764	MN792067	MT195275
AY463229	JN687776	L22939	MK384765	MN792068	MT195276
AY463230	JN687821	L22942	MK384772	MN792069	MT195277
AY463231	JN692431	L22943	MK384775	MN792070	MT195278
AY463232	JN692432	L22948	MK384778	MN792071	MT195279
AY463233	JN692433	L22949	MK384782	MN792072	MT195280
AY463234	JN692434	L22950	MK384784	MN792073	MT195281
AY463235	JN692435	L22955	MK384785	MN792074	MT195282
AY463236	JN692436	L22957	MK384786	MN792075	MT195283
AY463237	JN692437	L23065	MK384787	MN792076	MT195284
AY489739	JN692439	L39106	MK384789	MN792077	MT195285
AY489910	JN692440	LC027100	MK384790	MN792078	MT195286
AY489911	JN692442	LC312713	MK384792	MN792079	MT195287
AY489912	JN692443	LC312714	MK384797	MN792080	MT195288
AY489913	JN692444	LC312715	MK384798	MN792081	MT195289
AY489914	JN692445	M12507	MK384802	MN792082	MT195290
AY489915	JN692446	M13137	MK384803	MN792083	MT195291
AY489916	JN692447	M15654	MK384807	MN792084	MT195292
AY489917	JN692448	M15896	MK384808	MN792085	MT195293
AY489918	JN692449	M17450	MK384811	MN792086	MT195294
AY494966	JN692450	M17451	MK384813	MN792087	MT195295
AY494967	JN692451	M21098	MK384817	MN792088	MT195296
AY494968	JN692452	M21138	MK384818	MN792089	MT195297
AY494971	JN692453	M26727	MK384822	MN792090	MT195298
AY500393	JN692454	M27323	MK384827	MN792091	MT195299
AY505010	JN692455	M38430	MK384829	MN792092	MT195300
AY521629	JN692456	M38431	MK384830	MN792093	MT195301
AY521630	JN692457	M62320	MK384831	MN792094	MT195302
AY521631	JN692459	M66533	MK384835	MN792095	MT195303
AY521632	JN692460	M93258	MK384836	MN792096	MT195304
AY521633	JN692461	M95292	MK384838	MN792097	MT195305
AY522721	JN692462	MA255543	MK384840	MN792098	MT195306
AY522722	JN692463	MF073269	MK384844	MN792099	MT195307
AY522723	JN692465	MF084205	MK384850	MN792100	MT195308
AY522724	JN692467	MF109354	MK384854	MN792101	MT195309
AY522725	JN692468	MF109358	MK384855	MN792102	MT195310
AY522726	JN692470	MF109359	MK384858	MN792103	MT195311
AY522727	JN692471	MF109363	MK384863	MN792104	MT195312

AY522728	JN692472	MF109364	MK384866	MN792105	MT195313
AY522729	JN692473	MF109366	MK384868	MN792106	MT195314
AY522730	JN692474	MF109367	MK384870	MN792107	MT195315
AY522731	JN692475	MF109369	MK384880	MN792108	MT195316
AY522732	JN692476	MF109371	MK384885	MN792109	MT195317
AY522733	JN692478	MF109372	MK384888	MN792110	MT195318
AY522734	JN692479	MF109376	MK384890	MN792111	MT195319
AY522735	JN692480	MF109378	MK384891	MN792112	MT195320
AY522736	JN786724	MF109381	MK384901	MN792113	MT195321
AY529659	JN786767	MF109383	MK384904	MN792114	MT195322
AY529660	JN786795	MF109388	MK384909	MN792115	MT195323
AY529661	JN786861	MF109389	MK384911	MN792116	MT195324
AY529662	JN860761	MF109395	MK384915	MN792117	MT195325
AY529664	JN860762	MF109396	MK384916	MN792119	MT195326
AY529665	JN860763	MF109397	MK384919	MN792120	MT195327
AY529666	JN860764	MF109400	MK384921	MN792122	MT195328
AY529667	JN860765	MF109402	MK384928	MN792123	MT195329
AY529668	JN860766	MF109407	MK384930	MN792125	MT195330
AY529669	JN860767	MF109408	MK384931	MN792127	MT195331
AY529670	JN860768	MF109412	MK384935	MN792129	MT195332
AY529672	JN860769	MF109413	MK384937	MN792131	MT195333
AY529673	JN864047	MF109417	MK384942	MN792133	MT195334
AY529675	JN864048	MF109418	MK384946	MN792134	MT195335
AY529676	JN864049	MF109422	MK384948	MN792136	MT195336
AY529677	JN864050	MF109426	MK384949	MN792139	MT195337
AY529678	JN864051	MF109427	MK384954	MN792140	MT195338
AY532635	JN864052	MF109428	MK384955	MN792142	MT195339
AY535659	JN864053	MF109432	MK384961	MN792144	MT195340
AY535660	JN864054	MF109434	MK384963	MN792146	MT195341
AY536233	JN864055	MF109435	MK384964	MN792148	MT195342
AY536234	JN864056	MF109437	MK384966	MN792150	MT195343
AY536235	JN864057	MF109439	MK384972	MN792152	MT195344
AY536236	JN864058	MF109440	MK384973	MN792153	MT195345
AY536237	JN864059	MF109441	MK384975	MN792155	MT195346
AY536238	JN882651	MF109446	MK384978	MN792157	MT195347
AY560107	JN882652	MF109447	MK384980	MN792159	MT195348
AY560108	JN882653	MF109449	MK384987	MN792162	MT195349
AY560109	JN882654	MF109452	MK384991	MN792164	MT195350
AY560110	JN882655	MF109453	MK384992	MN792166	MT195351
AY561236	JN944654	MF109454	MK384999	MN792169	MT195352
AY561237	JN944655	MF109456	MK385001	MN792172	MT195353
AY561238	JN944656	MF109461	MK385002	MN792174	MT195354
AY561239	JN944657	MF109462	MK385003	MN792176	MT195355
AY561240	JN944658	MF109471	MK385011	MN792178	MT195356
AY563169	JN944659	MF109472	MK385012	MN792179	MT195357
AY563170	JN944663	MF109474	MK385016	MN792180	MT195358
AY585264	JN944664	MF109475	MK385017	MN792182	MT195359

AY585265	JN977603	MF109476	MK385025	MN792184	MT195360
AY585266	JN977604	MF109479	MK385026	MN792186	MT195361
AY585267	JN983803	MF109480	MK385028	MN792188	MT195362
AY585268	JN983804	MF109487	MK385040	MN792190	MT195363
AY586540	JN983805	MF109494	MK385044	MN792192	MT195364
AY586541	JQ061131	MF109499	MK385046	MN792194	MT195365
AY586542	JQ085287	MF109501	MK385047	MN792196	MT195366
AY586543	JQ085289	MF109504	MK385056	MN792198	MT195367
AY586544	JQ085291	MF109506	MK385061	MN792200	MT195368
AY586545	JQ085293	MF109508	MK385062	MN792201	MT195369
AY586546	JQ085295	MF109513	MK385066	MN792202	MT195370
AY586547	JQ085297	MF109516	MK385067	MN792203	MT195371
AY586548	JQ182824	MF109522	MK385068	MN792204	MT195372
AY586549	JQ182896	MF109523	MK385069	MN792205	MT195373
AY588970	JQ250832	MF109524	MK385071	MN792206	MT195374
AY588971	JQ250872	MF109525	MK385074	MN792207	MT195375
AY608576	JQ250925	MF109526	MK385082	MN792208	MT195376
AY612637	JQ251015	MF109527	MK385084	MN792209	MT195377
AY612855	JQ251105	MF109532	MK385091	MN792210	MT195378
AY618998	JQ292892	MF109533	MK385093	MN792211	MT195379
AY623599	JQ292900	MF109534	MK385096	MN792212	MT195380
AY624304	JQ316126	MF109535	MK385101	MN792213	MT195381
AY682547	JQ316127	MF109536	MK385103	MN792214	MT195382
AY703908	JQ316128	MF109538	MK385105	MN792215	MT195383
AY703909	JQ316129	MF109539	MK385106	MN792216	MT195384
AY703910	JQ316130	MF109540	MK385109	MN792217	MT195385
AY703911	JQ316131	MF109543	MK385110	MN792218	MT195386
AY713406	JQ316132	MF109546	MK385114	MN792219	MT195387
AY713408	JQ316134	MF109547	MK385115	MN792221	MT195388
AY713410	JQ316135	MF109550	MK385118	MN792223	MT195389
AY713411	JQ316136	MF109551	MK385119	MN792225	MT195390
AY713412	JQ341411	MF109555	MK385122	MN792227	MT195391
AY713413	JQ352781	MF109557	MK385124	MN792229	MT195392
AY713414	JQ361079	MF109558	MK385126	MN792231	MT195393
AY713415	JQ403019	MF109559	MK385132	MN792233	MT195394
AY713416	JQ403021	MF109561	MK385133	MN792235	MT195395
AY713417	JQ403022	MF109562	MK385134	MN792237	MT195396
AY713418	JQ403023	MF109563	MK385135	MN792239	MT195397
AY713419	JQ403024	MF109564	MK385137	MN792241	MT195398
AY713420	JQ403025	MF109568	MK385141	MN792242	MT195399
AY713421	JQ403026	MF109572	MK385142	MN792243	MT195400
AY713422	JQ403028	MF109578	MK385145	MN792244	MT195401
AY713423	JQ403029	MF109589	MK385147	MN792245	MT195402
AY713424	JQ403030	MF109590	MK385150	MN792246	MT195403
AY713425	JQ403035	MF109591	MK385151	MN792247	MT195404
AY727522	JQ403037	MF109592	MK385155	MN792248	MT195405
AY727523	JQ403038	MF109593	MK385156	MN792249	MT195406

AY727524	JQ403039	MF109595	MK385157	MN792250	MT195407
AY727525	JQ403041	MF109596	MK385160	MN792251	MT195408
AY727526	JQ403042	MF109598	MK385162	MN792252	MT195409
AY727527	JQ403043	MF109599	MK385179	MN792253	MT195410
AY734550	JQ403044	MF109605	MK385181	MN792254	MT195411
AY734551	JQ403045	MF109606	MK385188	MN792255	MT195412
AY734552	JQ403046	MF109607	MK385189	MN792256	MT195413
AY734553	JQ403047	MF109612	MK385192	MN792257	MT195414
AY734554	JQ403048	MF109613	MK385194	MN792258	MT195415
AY734555	JQ403049	MF109616	MK385195	MN792259	MT195416
AY734556	JQ403050	MF109617	MK385196	MN792260	MT195417
AY734558	JQ403056	MF109618	MK385198	MN792262	MT195418
AY734559	JQ403058	MF109620	MK385199	MN792264	MT195419
AY734560	JQ403059	MF109621	MK385200	MN792266	MT195420
AY734562	JQ403060	MF109623	MK385204	MN792268	MT195421
AY736810	JQ403061	MF109624	MK385205	MN792270	MT195422
AY736823	JQ403062	MF109627	MK385211	MN792272	MT195423
AY736838	JQ403063	MF109629	MK385213	MN792274	MT195424
AY736840	JQ403064	MF109630	MK385217	MN792276	MT195425
AY751406	JQ403066	MF109636	MK385220	MN792278	MT195426
AY751407	JQ403067	MF109637	MK385222	MN792281	MT195428
AY771588	JQ403068	MF109640	MK385223	MN792282	MT195429
AY771589	JQ403069	MF109647	MK385226	MN792283	MT195430
AY771590	JQ403070	MF109648	MK385229	MN792284	MT195431
AY771591	JQ403071	MF109649	MK385238	MN792285	MT195432
AY771592	JQ403073	MF109651	MK385240	MN792286	MT195433
AY771593	JQ403074	MF109656	MK385245	MN792287	MT195434
AY772535	JQ403075	MF109657	MK385247	MN792288	MT195435
AY772690	JQ403077	MF109664	MK385254	MN792289	MT195436
AY772691	JQ403078	MF109667	MK385260	MN792290	MT195437
AY772692	JQ403079	MF109670	MK385262	MN792292	MT195438
AY772693	JQ403080	MF109675	MK385264	MN792294	MT195439
AY772694	JQ403082	MF109676	MK385269	MN792296	MT195440
AY772695	JQ403083	MF109677	MK385270	MN792298	MT195441
AY772696	JQ403084	MF109679	MK385272	MN792300	MT195442
AY772698	JQ403085	MF109680	MK385275	MN792302	MT195443
AY772699	JQ403086	MF109684	MK385276	MN792306	MT195444
AY772700	JQ403087	MF109689	MK385277	MN792307	MT195445
AY772701	JQ403088	MF109695	MK385281	MN792309	MT195446
AY773338	JQ403089	MF109697	MK385283	MN792310	MT195447
AY773339	JQ403091	MF109700	MK385290	MN792312	MT195448
AY773340	JQ403092	MF109705	MK385299	MN792314	MT195449
AY773341	JQ403093	MF109706	MK385301	MN792316	MT195450
AY779550	JQ403094	MF109707	MK385305	MN792317	MT195451
AY779553	JQ403095	MF109708	MK385306	MN792319	MT195452
AY779557	JQ403096	MF109709	MK385310	MN792321	MT195453
AY781126	JQ403097	MF109710	MK385324	MN792323	MT195454

AY781127	JQ403099	MF109713	MK385327	MN792325	MT195455
AY781128	JQ403100	MF157740	MK385331	MN792327	MT195456
AY795903	JQ403101	MF157742	MK385337	MN792329	MT195457
AY795904	JQ403102	MF284922	MK385338	MN792331	MT195458
AY795905	JQ403103	MF284939	MK385345	MN792332	MT195459
AY795906	JQ403104	MF284954	MK385347	MN792333	MT195460
AY795907	JQ403105	MF284960	MK385349	MN792334	MT195461
AY805330	JQ403106	MF284964	MK385350	MN792335	MT195462
AY812751	JQ403107	MF284974	MK385351	MN792336	MT195463
AY818644	JQ423923	MF284991	MK385353	MN792337	MT195464
AY819715	JQ429433	MF285020	MK385354	MN792338	MT195465
AY829203	JQ609684	MF285052	MK385355	MN792339	MT195466
AY829204	JQ609870	MF372644	MK385363	MN792340	MT195467
AY829205	JQ609973	MF372645	MK385366	MN792343	MT195468
AY829206	JQ610053	MF372646	MK385374	MN792345	MT195469
AY829207	JQ610120	MF372647	MK385376	MN792347	MT195470
AY829208	JQ730708	MF372648	MK385379	MN792350	MT195471
AY829209	JQ777026	MF372649	MK385381	MN792353	MT195472
AY829210	JQ777061	MF372650	MK385386	MN792355	MT195473
AY829212	JQ777087	MF372651	MK385395	MN792357	MT195474
AY829214	JQ777111	MF373124	MK385397	MN792358	MT195475
AY835435	JQ777137	MF373125	MK385401	MN792359	MT195476
AY835436	JQ777145	MF373126	MK385402	MN792360	MT195477
AY835437	JQ779074	MF373127	MK385406	MN792361	MT195478
AY835438	JQ779170	MF373128	MK385410	MN792362	MT195479
AY835442	JQ779226	MF373129	MK385412	MN792363	MT195480
AY835443	JQ779286	MF373130	MK385416	MN792364	MT195481
AY835444	JX112796	MF373131	MK385420	MN792365	MT195482
AY835445	JX112797	MF373132	MK385422	MN792366	MT195483
AY835446	JX112798	MF373133	MK385423	MN792368	MT195484
AY835447	JX112799	MF373134	MK385428	MN792370	MT195485
AY835448	JX112800	MF373135	MK385429	MN792372	MT195486
AY835449	JX112801	MF373136	MK385434	MN792374	MT195487
AY835450	JX112802	MF373137	MK385438	MN792375	MT195488
AY835451	JX112803	MF373138	MK385439	MN792377	MT195489
AY835452	JX112804	MF373139	MK385444	MN792379	MT195490
AY835748	JX112805	MF373140	MK385445	MN792381	MT195491
AY835753	JX112806	MF373141	MK385446	MN792383	MT195492
AY835754	JX112807	MF373142	MK385456	MN792385	MT195493
AY835755	JX112808	MF373143	MK385459	MN792387	MT195494
AY835757	JX112809	MF373144	MK385460	MN792389	MT195495
AY835759	JX112810	MF373145	MK385461	MN792390	MT195496
AY835762	JX112811	MF373146	MK385462	MN792392	MT195497
AY835766	JX112813	MF373147	MK385464	MN792394	MT195498
AY835769	JX112814	MF373148	MK385465	MN792396	MT195499
AY835770	JX112815	MF373149	MK385466	MN792398	MT195500
AY835774	JX112816	MF373150	MK385467	MN792400	MT195501

AY835777	JX112817	MF373151	MK385480	MN792401	MT195502
AY835779	JX112818	MF373152	MK385481	MN792402	MT195503
AY839827	JX112819	MF373153	MK385483	MN792407	MT195504
AY842786	JX112820	MF373154	MK385484	MN792410	MT195505
AY842808	JX112821	MF373155	MK385485	MN792411	MT195506
AY842824	JX112822	MF373156	MK385491	MN792413	MT195507
AY857022	JX112823	MF373157	MK385494	MN792415	MT195508
AY857052	JX112824	MF373158	MK385499	MN792417	MT195509
AY857127	JX112825	MF373159	MK385500	MN792419	MT195510
AY857144	JX112826	MF373160	MK385503	MN792421	MT195511
AY857165	JX112827	MF373161	MK385504	MN792423	MT195512
AY878054	JX112828	MF373162	MK385505	MN792424	MT195513
AY878055	JX112829	MF373163	MK385508	MN792425	MT195514
AY878056	JX112830	MF373164	MK385509	MN792426	MT195515
AY878057	JX112831	MF373165	MK385511	MN792427	MT195516
AY878058	JX112832	MF373166	MK385514	MN792428	MT195517
AY878059	JX112833	MF373167	MK385515	MN792429	MT195518
AY878060	JX112834	MF373168	MK385516	MN792430	MT195519
AY878061	JX112835	MF373169	MK385518	MN792431	MT195520
AY878062	JX112836	MF373170	MK385519	MN792432	MT195521
AY878063	JX112837	MF373171	MK385520	MN792433	MT195522
AY878064	JX112838	MF373172	MK385523	MN792434	MT195523
AY878065	JX112839	MF373173	MK385525	MN792435	MT195524
AY878068	JX112840	MF373174	MK385526	MN792437	MT195525
AY878069	JX112841	MF373175	MK385528	MN792438	MT195526
AY878070	JX112842	MF373176	MK385530	MN792439	MT195527
AY878071	JX112843	MF373177	MK385533	MN792440	MT195528
AY878072	JX112844	MF373178	MK385534	MN792441	MT195529
AY882421	JX112845	MF373179	MK385538	MN792442	MT195530
AY887501	JX112846	MF373180	MK385539	MN792443	MT195532
AY894993	JX112847	MF373181	MK385542	MN792444	MT195533
AY894994	JX112848	MF373182	MK385543	MN792445	MT195535
AY894995	JX112849	MF373183	MK385548	MN792447	MT222937
AY894996	JX112850	MF373184	MK385551	MN792449	MT222938
AY900571	JX112851	MF373185	MK385552	MN792451	MT222939
AY900572	JX112852	MF373186	MK385556	MN792453	MT222940
AY900574	JX112853	MF373187	MK385557	MN792455	MT222941
AY900575	JX112854	MF373188	MK385560	MN792457	MT222942
AY900576	JX112855	MF373189	MK385576	MN792459	MT222943
AY901965	JX112856	MF373190	MK385578	MN792461	MT222944
AY901966	JX112857	MF373191	MK385581	MN792463	MT222945
AY901967	JX112858	MF373192	MK385582	MN792465	MT222946
AY901968	JX112859	MF373193	MK385584	MN792466	MT222947
AY901969	JX112860	MF373194	MK385589	MN792467	MT222948
AY901970	JX112861	MF373195	MK397789	MN792468	MT222949
AY901971	JX112862	MF373196	MK397970	MN792469	MT222950
AY901972	JX112863	MF373197	MK412338	MN792470	MT222951

AY901973	JX112864	MF373198	MK412339	MN792471	MT222952
AY901974	JX112865	MF373199	MK457775	MN792472	MT222953
AY901975	JX112866	MF373200	MK457782	MN792473	MT222954
AY901976	JX112867	MF373201	MK457783	MN792474	MT222955
AY901977	JX112868	MF373202	MK457788	MN792475	MT222956
AY901978	JX112869	MF373203	MK457789	MN792476	MT222957
AY901979	JX112870	MF373204	MK457794	MN792477	MT222958
AY901980	JX140646	MF373205	MK457795	MN792478	MT222959
AY901981	JX140647	MF373206	MK457796	MN792479	MT222960
AY905493	JX140652	MF373389	MK457797	MN792481	MT222961
AY905494	JX140654	MF379808	MK457798	MN792483	MT224125
AY905495	JX140656	MF381270	MK457800	MN792485	MT224126
AY905496	JX140657	MF381271	MK457801	MN792487	MT224127
AY905497	JX140658	MF381272	MK457802	MN792489	MT227374
AY945710	JX140659	MF381273	MK457803	MN792491	MT227375
AY945711	JX140660	MF381274	MK457804	MN792493	MT227376
AY945712	JX140661	MF381275	MK457807	MN792495	MT227377
AY945713	JX140667	MF381276	MK457808	MN792497	MT227379
AY945716	JX140668	MF381277	MK457809	MN792499	MT227380
AY945717	JX140670	MF381278	MK457811	MN792501	MT227381
AY945718	JX140671	MF381279	MK457812	MN792503	MT227384
AY945719	JX140672	MF381280	MK457813	MN792506	MT227385
AY945720	JX140673	MF381281	MK457814	MN792508	MT227386
AY945721	JX140676	MF381282	MK457816	MN792511	MT227387
AY945722	JX140679	MF381283	MK457822	MN792513	MT227388
AY945724	JX202955	MF381284	MK457823	MN792515	MT227389
AY945725	JX202976	MF381285	MK457825	MN792517	MT227390
AY945726	JX203061	MF381286	MK457832	MN792518	MT227391
AY945727	JX203082	MF499616	MK457840	MN792519	MT227392
AY945728	JX203100	MF500271	MK457849	MN792520	MT227393
AY945730	JX203116	MF500301	MK457850	MN792521	MT227394
AY945731	JX213352	MF501101	MK457851	MN792522	MT227395
AY945732	JX213359	MF510462	MK457859	MN792523	MT227396
AY945733	JX213375	MF565934	MK457862	MN792524	MT227397
AY945734	JX213387	MF591581	MK457873	MN792525	MT227398
AY945736	JX213405	MF614605	MK457876	MN792526	MT227399
AY945737	JX213416	MF614606	MK457884	MN792527	MT227400
AY945738	JX213435	MF614608	MK457894	MN792528	MT227401
AY945739	JX213443	MF614609	MK457899	MN792529	MT227402
AY967803	JX213454	MF614611	MK457907	MN792530	MT227403
AY967804	JX213464	MF614612	MK457910	MN792531	MT227404
AY967805	JX236678	MF614613	MK457915	MN792532	MT227405
AY967806	JX239287	MF614614	MK457919	MN792533	MT227406
AY967807	JX239329	MF614615	MK457928	MN792534	MT227407
AY968312	JX239342	MF767262	MK457929	MN792535	MT227408
AY970946	JX245014	MF957351	MK457933	MN792536	MT227409
D10112	JX245015	MF957426	MK457935	MN792537	MT227410

D12582	JX390610	MF957454	MK457949	MN792538	MT227411
DL258709	JX390611	MF957493	MK457958	MN792539	MT227412
DL258716	JX390612	MF957508	MK457962	MN792540	MT227413
DQ007901	JX390976	MF957553	MK457967	MN792541	MT227414
DQ007902	JX390977	MF957573	MK457968	MN792542	MT227415
DQ007903	JX392378	MF957603	MK457970	MN792543	MT227416
DQ011165	JX392380	MF957618	MK457971	MN792544	MT227417
DQ011166	JX392381	MF957663	MK457972	MN792545	MT227418
DQ011167	JX392382	MF957678	MK457974	MN792546	MT227419
DQ011169	JX392383	MF990465	MK457975	MN792547	MT227420
DQ011170	JX392384	MG000979	MK457976	MN792548	MT227421
DQ011171	JX422193	MG064457	MK457978	MN792549	MT227422
DQ011172	JX422195	MG171201	MK457979	MN792550	MT227423
DQ011173	JX422196	MG196359	MK457982	MN792551	MT227424
DQ011174	JX422197	MG196438	MK457985	MN792552	MT227425
DQ011175	JX422199	MG196549	MK457988	MN792553	MT227426
DQ011176	JX422200	MG196642	MK457989	MN792554	MT227427
DQ011177	JX422201	MG196653	MK457990	MN792556	MT227428
DQ011178	JX422202	MG196663	MK457991	MN792557	MT227429
DQ011179	JX422203	MG196679	MK457992	MN792558	MT227430
DQ011180	JX422204	MG196689	MK457993	MN792559	MT227431
DQ017382	JX422207	MG196699	MK457995	MN792560	MT227432
DQ017383	JX422209	MG196711	MK457998	MN792561	MT227433
DQ020274	JX422210	MG196732	MK457999	MN792562	MT227434
DQ054367	JX422211	MG196745	MK458000	MN792563	MT227435
DQ056404	JX446655	MG196755	MK458002	MN792564	MT227436
DQ056405	JX446666	MG196765	MK458005	MN792565	MT227437
DQ056406	JX446698	MG196775	MK458006	MN792566	MT227438
DQ056408	JX446710	MG196787	MK458007	MN792568	MT227439
DQ056409	JX446728	MG196797	MK458008	MN792569	MT227440
DQ056410	JX446736	MG196816	MK458009	MN792570	MT227441
DQ056411	JX446755	MG196822	MK458012	MN792571	MT227442
DQ056412	JX446775	MG196832	MK458015	MN792572	MT227443
DQ056413	JX446790	MG196836	MK458018	MN792573	MT227444
DQ056414	JX446795	MG196846	MK458020	MN792574	MT227445
DQ056415	JX446817	MG196866	MK458022	MN792575	MT227446
DQ056416	JX446837	MG196877	MK458024	MN792576	MT227447
DQ056417	JX446848	MG196902	MK458025	MN792577	MT227448
DQ056418	JX446853	MG196912	MK458026	MN792578	MT227449
DQ083238	JX446873	MG196922	MK458027	MN792579	MT227450
DQ085867	JX446888	MG196942	MK458028	MN829458	MT227451
DQ085868	JX446899	MG196952	MK458031	MN829459	MT227452
DQ085869	JX446910	MG196963	MK458033	MN870987	MT227453
DQ085870	JX446916	MG196973	MK458040	MN870988	MT227454
DQ085871	JX446927	MG196997	MK458042	MN870989	MT227455
DQ085872	JX446942	MG197013	MK458048	MN870990	MT227456
DQ085873	JX446960	MG197023	MK458050	MN870991	MT227457

DQ085874	JX446976	MG197033	MK458051	MN870992	MT227458
DQ085875	JX446987	MG197043	MK458053	MN870993	MT227459
DQ085876	JX446997	MG197054	MK458054	MN870994	MT227460
DQ093585	JX447013	MG197064	MK458055	MN870995	MT227461
DQ093586	JX447018	MG197074	MK458056	MN870996	MT227462
DQ093587	JX447028	MG197084	MK458057	MN870997	MT227463
DQ093588	JX447043	MG197105	MK458058	MN870998	MT227464
DQ093589	JX447054	MG197115	MK458061	MN870999	MT227465
DQ093590	JX447063	MG197126	MK458064	MN871000	MT227466
DQ093591	JX447072	MG197136	MK458069	MN871001	MT227467
DQ093592	JX447077	MG197146	MK458070	MN871002	MT227468
DQ093593	JX447087	MG197164	MK458071	MN871003	MT227469
DQ093594	JX447102	MG197176	MK458072	MN871004	MT227470
DQ093595	JX447117	MG197187	MK458073	MN871005	MT227471
DQ093596	JX447122	MG197197	MK458074	MN871006	MT227472
DQ093597	JX447132	MG197210	MK458075	MN871007	MT227473
DQ093598	JX447146	MG244243	MK458077	MN871008	MT227474
DQ093599	JX447156	MG365762	MK458078	MN871009	MT227475
DQ093600	JX447177	MG365763	MK458079	MN871010	MT227476
DQ093601	JX447199	MG365764	MK458080	MN871011	MT227477
DQ093602	JX447220	MG365765	MK458081	MN871012	MT227478
DQ093604	JX447235	MG365766	MK458082	MN871013	MT227479
DQ093605	JX447252	MG365767	MK458083	MN871014	MT227480
DQ093606	JX447266	MG365768	MK458084	MN871015	MT227481
DQ093607	JX447283	MG365769	MK458085	MN871016	MT227482
DQ127534	JX447294	MG365770	MK458086	MN871017	MT227483
DQ127537	JX447300	MG365771	MK458087	MN871018	MT227484
DQ127548	JX447305	MG518476	MK458088	MN871019	MT227485
DQ141204	JX447312	MG518477	MK458089	MN871020	MT227486
DQ141341	JX447315	MG519330	MK458090	MN871021	MT227487
DQ141342	JX447328	MG571981	MK458091	MN871022	MT227488
DQ141343	JX447344	MG571982	MK458092	MN871023	MT227489
DQ141344	JX447349	MG571983	MK458093	MN871024	MT227490
DQ141345	JX447359	MG571985	MK458094	MN871025	MT227491
DQ164104	JX447379	MG571986	MK458095	MN871026	MT227492
DQ164106	JX447395	MG571987	MK458096	MN871027	MT227493
DQ164107	JX447402	MG571988	MK458098	MN871028	MT227494
DQ164108	JX447412	MG571990	MK458099	MN871029	MT227495
DQ164109	JX447427	MG571991	MK458100	MN871030	MT276997
DQ164110	JX447447	MG571996	MK458101	MN871031	MT276998
DQ164111	JX447454	MG571999	MK458102	MN871032	MT276999
DQ164113	JX447465	MG572001	MK458104	MN871033	MT277000
DQ164114	JX447480	MG572002	MK458106	MN871034	MT277001
DQ164115	JX447493	MG572006	MK458109	MN871035	MT277002
DQ164117	JX447499	MG572010	MK458111	MN871036	MT307344
DQ164118	JX447515	MG572011	MK458117	MN871037	MT307345
DQ164119	JX447529	MG655196	MK458118	MN871038	MT307346

DQ164121	JX447535	MG655197	MK458120	MN871039	MT307347
DQ164122	JX447550	MG655198	MK458121	MN871040	MT307348
DQ164126	JX447561	MG655199	MK458123	MN871041	MT307349
DQ164127	JX447579	MG655200	MK458125	MN871042	MT307350
DQ164129	JX447589	MG655201	MK458126	MN871043	MT307351
DQ167215	JX447608	MG655202	MK458129	MN871044	MT307352
DQ168573	JX447637	MG655203	MK458130	MN871045	MT307353
DQ168575	JX447657	MG655204	MK458136	MN871046	MT307354
DQ168576	JX447668	MG655205	MK458137	MN871047	MT307355
DQ168577	JX447678	MG655206	MK458140	MN871048	MT307356
DQ168578	JX447699	MG655207	MK458142	MN871049	MT307357
DQ168579	JX447709	MG655208	MK458144	MN871050	MT307358
DQ178989	JX447721	MG655209	MK458147	MN871051	MT307359
DQ187009	JX447726	MG655210	MK458151	MN871052	MT307360
DQ189088	JX447746	MG655211	MK458152	MN871053	MT307361
DQ207940	JX447773	MG655212	MK458153	MN871054	MT307362
DQ207941	JX447795	MG655213	MK458154	MN871055	MT307363
DQ207942	JX447814	MG655214	MK458157	MN871056	MT307364
DQ207943	JX447827	MG655215	MK458166	MN871057	MT307365
DQ207944	JX447833	MG655216	MK458171	MN871058	MT307366
DQ208424	JX447853	MG655217	MK458173	MN871059	MT307367
DQ208431	JX447875	MG655218	MK458181	MN871060	MT307368
DQ208434	JX447884	MG742702	MK458183	MN871061	MT307369
DQ208441	JX447902	MG760371	MK458184	MN871062	MT307370
DQ208444	JX447921	MG760372	MK458185	MN871063	MT307371
DQ208447	JX447941	MG760373	MK458191	MN871064	MT307372
DQ208449	JX447958	MG760374	MK458194	MN871065	MT307373
DQ208456	JX447978	MG760375	MK458208	MN871066	MT307374
DQ208459	JX448001	MG760376	MK458216	MN871067	MT307375
DQ208465	JX448016	MG760377	MK458252	MN871068	MT307376
DQ208468	JX448019	MG760378	MK458262	MN871069	MT307377
DQ208478	JX448028	MG760379	MK458263	MN871070	MT307378
DQ208483	JX448039	MG760380	MK474158	MN871071	MT307379
DQ208489	JX448051	MG760381	MK493076	MN871072	MT307380
DQ208492	JX448057	MG760382	MK493077	MN871073	MT307381
DQ208499	JX448071	MG760383	MK493078	MN871074	MT307382
DQ222211	JX448086	MG760384	MK493079	MN871075	MT307383
DQ230841	JX448101	MG760386	MK499378	MN871076	MT307384
DQ230842	JX448198	MG760387	MK501552	MN871077	MT307385
DQ234790	JX448217	MG760388	MK501553	MN871078	MT307386
DQ275642	JX448238	MG760389	MK501554	MN871079	MT307387
DQ275643	JX448253	MG760390	MK501555	MN871080	MT307388
DQ275644	JX448270	MG760391	MK501556	MN871081	MT307389
DQ275645	JX448279	MG760393	MK501557	MN871082	MT307390
DQ275646	JX448289	MG760395	MK501558	MN871083	MT307391
DQ275647	JX448301	MG760396	MK501559	MN871084	MT307392
DQ275648	JX448312	MG760397	MK501560	MN871085	MT307393

DQ275649	JX500694	MG760398	MK501561	MN871086	MT307394
DQ275650	JX500695	MG760399	MK501562	MN871087	MT307395
DQ275651	JX500696	MG760400	MK501563	MN871088	MT307396
DQ275652	JX500697	MG760401	MK501564	MN871089	MT307397
DQ275653	JX500698	MG760402	MK501565	MN871090	MT307398
DQ275654	JX500699	MG760404	MK501566	MN871091	MT307399
DQ275655	JX500700	MG760405	MK501567	MN871092	MT307400
DQ275656	JX500701	MG760406	MK501568	MN871093	MT307401
DQ275657	JX500702	MG760407	MK501569	MN871094	MT307402
DQ275658	JX500703	MG760408	MK501570	MN871095	MT307403
DQ275659	JX500704	MG760409	MK501571	MN871096	MT307404
DQ275660	JX500705	MG760410	MK501572	MN871097	MT307405
DQ275661	JX500706	MG760412	MK501573	MN871098	MT307406
DQ275664	JX500707	MG760413	MK501574	MN871099	MT307407
DQ295192	JX500708	MG760414	MK501575	MN871100	MT307408
DQ295193	JX500709	MG760415	MK501576	MN871101	MT307409
DQ295195	JX503071	MG760416	MK501577	MN871102	MT307410
DQ295196	JX503074	MG760417	MK501578	MN871103	MT307411
DQ313239	JX503075	MG760419	MK501579	MN871104	MT307412
DQ313246	JX512892	MG760420	MK501580	MN871105	MT307413
DQ313247	JX512893	MG760421	MK501581	MN871106	MT307414
DQ313248	JX512894	MG760422	MK501582	MN871107	MT307415
DQ313249	JX512895	MG760423	MK501583	MN871108	MT307416
DQ313250	JX512896	MG760424	MK501584	MN871109	MT307417
DQ313252	JX512898	MG760425	MK501586	MN871110	MT307418
DQ313253	JX512899	MG760426	MK501587	MN871111	MT307419
DQ313254	JX512901	MG760428	MK501588	MN871112	MT307420
DQ314731	JX574661	MG760429	MK501589	MN871113	MT307421
DQ314732	JX574662	MG760430	MK501590	MN871114	MT307422
DQ322223	JX574663	MG760431	MK501591	MN871115	MT307423
DQ322225	JX658588	MG839510	MK501592	MN871116	MT307424
DQ322227	JX658600	MG839511	MK501593	MN871117	MT307425
DQ322233	JX658625	MG839512	MK501594	MN871118	MT307426
DQ339425	JX658657	MG839513	MK501595	MN871119	MT307427
DQ351216	JX679207	MG839514	MK501596	MN871120	MT307428
DQ351217	JX845585	MG839515	MK501597	MN871121	MT307429
DQ351218	JX845589	MG839516	MK501598	MN871122	MT307430
DQ351219	JX845592	MG839517	MK501599	MN871123	MT307431
DQ351220	JX845596	MG839518	MK501600	MN871124	MT307432
DQ351221	JX845599	MG839519	MK501601	MN871125	MT307433
DQ351222	JX845602	MG840836	MK501602	MN871126	MT307434
DQ351223	JX845604	MG840837	MK501603	MN871127	MT307435
DQ351224	JX845608	MG840838	MK521804	MN871128	MT307436
DQ351225	JX845611	MG840839	MK521805	MN871129	MT307437
DQ351226	JX845612	MG840840	MK521806	MN871130	MT307438
DQ351227	JX848346	MG840841	MK521807	MN871131	MT307439
DQ351228	JX848348	MG840842	MK521808	MN871132	MT307440

DQ351229	JX848350	MG897823	MK564326	MN871133	MT307441
DQ351230	JX863919	MG898887	MK577478	MN871134	MT307442
DQ351231	JX863920	MG900587	MK577479	MN871135	MT307443
DQ351232	JX863921	MG901243	MK577480	MN871136	MT307444
DQ351234	JX863922	MG902036	MK577481	MN871137	MT307445
DQ351235	JX863923	MG902171	MK634699	MN871138	MT307446
DQ351237	JX863924	MG902325	MK643537	MN871139	MT307447
DQ354112	JX863965	MG902510	MK643538	MN871140	MT307448
DQ354113	JX863966	MG902773	MK643539	MN871141	MT307449
DQ354114	JX863967	MG902950	MK643540	MN871142	MT307450
DQ354115	JX863968	MG902951	MK643541	MN871143	MT307451
DQ354116	JX863969	MG989490	MK643542	MN871144	MT307452
DQ354117	JX863970	MG989491	MK643543	MN871145	MT307453
DQ354118	JX863971	MG989492	MK643544	MN871146	MT307454
DQ354119	JX863972	MG989493	MK643545	MN871147	MT307455
DQ354120	JX863983	MG989494	MK643547	MN871148	MT307456
DQ354121	JX863984	MG989495	MK643548	MN871149	MT307457
DQ354122	JX863985	MG989496	MK643549	MN871150	MT307458
DQ354123	JX863986	MG989497	MK643550	MN871151	MT307459
DQ358799	JX863987	MG989498	MK643551	MN871152	MT307460
DQ358800	JX863988	MG989499	MK643559	MN871153	MT307461
DQ358801	JX863989	MG989500	MK643564	MN871154	MT307462
DQ358802	JX863990	MG989501	MK643565	MN871155	MT307463
DQ358803	JX863991	MG989502	MK643569	MN871156	MT307464
DQ358804	JX863992	MG989503	MK643571	MN871157	MT307465
DQ358805	JX863993	MG989504	MK643572	MN871158	MT307466
DQ358806	JX863994	MG989505	MK643573	MN871159	MT307467
DQ358807	JX863995	MG989506	MK643575	MN871160	MT307468
DQ358808	JX864006	MG989507	MK643577	MN871161	MT307469
DQ358809	JX864007	MG989508	MK643581	MN871162	MT307470
DQ358810	JX864008	MG989509	MK643584	MN871163	MT307471
DQ358811	JX864019	MG989510	MK643585	MN871164	MT307472
DQ358812	JX864020	MG989511	MK643586	MN871165	MT307473
DQ366659	JX864021	MG989512	MK643588	MN871166	MT307474
DQ366660	JX864022	MG989514	MK643591	MN871167	MT307475
DQ366661	JX864023	MG989515	MK643595	MN871168	MT307476
DQ366662	JX864024	MG989516	MK643598	MN871169	MT307477
DQ366663	JX864025	MG989517	MK643600	MN871170	MT307478
DQ366664	JX864026	MG989518	MK643602	MN871171	MT307479
DQ366665	JX864027	MG989519	MK643604	MN871172	MT307480
DQ366666	JX877522	MG989520	MK643608	MN871173	MT307481
DQ369976	JX960597	MG989521	MK643610	MN871174	MT307482
DQ369977	JX960598	MG989522	MK643611	MN871175	MT307483
DQ369978	JX960599	MG989523	MK643613	MN871176	MT307484
DQ369979	JX960600	MG989524	MK643614	MN871177	MT307485
DQ369980	JX960601	MG989525	MK643615	MN871178	MT307486
DQ369981	JX960602	MG989526	MK643616	MN871179	MT307487

DQ369982	JX960603	MG989527	MK643618	MN871180	MT307488
DQ369983	JX960604	MG989528	MK643620	MN871181	MT307489
DQ369984	JX960605	MG989529	MK643622	MN871182	MT307490
DQ369985	JX960606	MG989530	MK643624	MN871183	MT307491
DQ369986	JX960607	MG989531	MK643629	MN871184	MT307492
DQ369987	JX960608	MG989532	MK643631	MN871185	MT307493
DQ369988	JX960609	MG989533	MK643632	MN871186	MT307494
DQ369989	JX960610	MG989534	MK643636	MN871187	MT307495
DQ369990	JX960611	MG989535	MK643639	MN871188	MT307496
DQ369991	JX960612	MG989536	MK643641	MN871189	MT307497
DQ369992	JX960613	MG989537	MK643650	MN871190	MT307498
DQ369993	JX960614	MG989538	MK643653	MN871191	MT307499
DQ369994	JX960615	MG989539	MK643654	MN871192	MT307500
DQ369995	JX960616	MG989540	MK643655	MN871193	MT307501
DQ369996	JX960617	MG989541	MK643656	MN871194	MT307502
DQ369997	JX960618	MG989542	MK643660	MN871195	MT307503
DQ382361	JX960619	MG989543	MK643661	MN871196	MT307504
DQ382362	JX960620	MG989544	MK643663	MN871197	MT307505
DQ382363	JX960621	MG989545	MK643664	MN871198	MT307506
DQ382364	JX960622	MG989546	MK643669	MN871199	MT307507
DQ382365	JX960623	MG989547	MK643671	MN871200	MT307508
DQ382366	JX960624	MG989548	MK643672	MN871201	MT307509
DQ382367	JX960625	MG989549	MK643674	MN871202	MT307510
DQ382368	JX960626	MG989550	MK643676	MN871203	MT307511
DQ382369	JX960627	MG989551	MK643677	MN871204	MT307512
DQ382370	JX960628	MG989552	MK643686	MN871205	MT307513
DQ382371	JX960629	MG989553	MK643687	MN871206	MT307514
DQ382372	JX960630	MG989554	MK643690	MN871207	MT307515
DQ382373	JX960631	MG989555	MK643692	MN871208	MT307516
DQ382374	JX960632	MG989556	MK643697	MN871209	MT307517
DQ382375	JX960633	MG989557	MK643698	MN871210	MT307518
DQ382376	JX960634	MG989558	MK643705	MN871211	MT307519
DQ382377	JX960635	MG989559	MK643707	MN871212	MT307520
DQ382378	JX960636	MG989560	MK643709	MN871213	MT307521
DQ382379	JX960637	MG989561	MK643713	MN871214	MT307522
DQ382380	JX960638	MG989562	MK643715	MN871215	MT307523
DQ383746	JX960639	MG989563	MK643718	MN871216	MT307524
DQ383747	JX972390	MG989564	MK643719	MN871217	MT307525
DQ383748	JX972931	MG989565	MK643729	MN871218	MT307526
DQ383749	JX972986	MG989566	MK643730	MN871219	MT307527
DQ383750	JX973075	MG989567	MK643732	MN871220	MT307528
DQ383751	JX973171	MG989568	MK643734	MN871221	MT307529
DQ383752	K02007	MG989569	MK643736	MN871222	MT307530
DQ383753	K03454	MG989570	MK643740	MN871223	MT307531
DQ383754	K03458	MG989571	MK643743	MN871224	MT307532
DQ383755	KC148637	MG989572	MK643744	MN871225	MT307533
DQ388514	KC149470	MG989573	MK643746	MN871226	MT307534

DQ388515	KC154012	MG989574	MK643747	MN871227	MT307535
DQ388516	KC154013	MG989575	MK643754	MN871228	MT307536
DQ388517	KC154014	MG989576	MK643756	MN871229	MT307537
DQ396364	KC154015	MG989577	MK643759	MN871230	MT307538
DQ396365	KC154016	MG989578	MK643760	MN871231	MT307539
DQ396367	KC154017	MG989579	MK643761	MN871232	MT307540
DQ396368	KC154018	MG989580	MK643764	MN871233	MT307541
DQ396369	KC154019	MG989582	MK643766	MN871234	MT307542
DQ396370	KC154020	MG989583	MK643771	MN871235	MT307543
DQ396371	KC154021	MG989584	MK643776	MN871236	MT307544
DQ396372	KC154022	MG989585	MK643779	MN871237	MT307545
DQ396373	KC154023	MG989586	MK643780	MN871238	MT307546
DQ396374	KC154024	MG989587	MK643781	MN871239	MT307547
DQ396375	KC154025	MG989588	MK643782	MN871240	MT307548
DQ396376	KC154026	MG989589	MK643783	MN871241	MT307549
DQ396377	KC154027	MG989590	MK643785	MN871242	MT307550
DQ396378	KC154028	MG989591	MK643787	MN871243	MT307551
DQ396380	KC156216	MG989592	MK643788	MN871244	MT307552
DQ396381	KC183774	MG989593	MK643791	MN871245	MT307553
DQ396382	KC183777	MG989594	MK643792	MN871246	MT307554
DQ396383	KC183778	MG989595	MK643794	MN871247	MT307555
DQ396384	KC183779	MG989596	MK643797	MN871248	MT307556
DQ396385	KC183780	MG989597	MK643802	MN871249	MT307557
DQ396386	KC183781	MG989598	MK643803	MN871250	MT307558
DQ396387	KC183782	MG989599	MK643825	MN871251	MT307559
DQ396388	KC183783	MG989601	MK650464	MN871252	MT307560
DQ396389	KC186127	MG989602	MK697709	MN871253	MT307561
DQ396390	KC186165	MG989603	MK749242	MN871254	MT307562
DQ396391	KC186203	MG989604	MK749243	MN871255	MT307563
DQ396392	KC186237	MG989605	MK749244	MN871256	MT307564
DQ396393	KC186271	MG989606	MK749245	MN871257	MT307565
DQ396394	KC186308	MG989607	MK749246	MN871258	MT307566
DQ396395	KC186349	MG989608	MK749247	MN871259	MT307567
DQ396398	KC186388	MG989609	MK749248	MN871260	MT307568
DQ396399	KC186421	MG989610	MK749249	MN871261	MT307569
DQ396400	KC186457	MG989611	MK749250	MN871262	MT307570
DQ400856	KC186498	MG989612	MK749251	MN871263	MT307571
DQ404010	KC186544	MG989613	MK749252	MN871264	MT307572
DQ410040	KC186577	MG989614	MK749253	MN871265	MT307573
DQ410062	KC186611	MG989615	MK749254	MN871266	MT307574
DQ410069	KC186650	MG989616	MK749255	MN871267	MT307575
DQ410087	KC186693	MG989617	MK749256	MN871268	MT307576
DQ410101	KC186730	MG989618	MK749257	MN871269	MT307577
DQ410109	KC186771	MG989619	MK749258	MN871270	MT307578
DQ410131	KC186814	MG989620	MK749259	MN871271	MT307579
DQ410188	KC186855	MG989668	MK749260	MN871272	MT307580
DQ410217	KC186893	MG989669	MK749261	MN871273	MT307581

DQ410227	KC186934	MG989670	MK749262	MN871274	MT307582
DQ410246	KC186976	MG989671	MK749263	MN871275	MT307583
DQ410284	KC187022	MH000288	MK749264	MN871276	MT307584
DQ410325	KC187067	MH000289	MK749265	MN871277	MT307585
DQ410373	KC187107	MH000290	MK749266	MN871278	MT307586
DQ410404	KC187145	MH000291	MK749267	MN871279	MT307587
DQ410427	KC187179	MH000292	MK749268	MN871280	MT307588
DQ410479	KC187216	MH000293	MK749269	MN871281	MT307589
DQ410511	KC187247	MH000294	MK749270	MN871282	MT307590
DQ410527	KC187280	MH000295	MK749271	MN871283	MT307591
DQ410528	KC187314	MH000296	MK749272	MN871284	MT307592
DQ410542	KC187349	MH000297	MK749273	MN871285	MT307593
DQ410560	KC187374	MH000298	MK749274	MN871286	MT307594
DQ410573	KC187417	MH000299	MK749275	MN871287	MT307595
DQ410588	KC187461	MH000300	MK749276	MN871288	MT307596
DQ410607	KC187499	MH000301	MK749277	MN871289	MT307597
DQ410619	KC187538	MH000302	MK749278	MN871290	MT307598
DQ411853	KC187577	MH000303	MK749279	MN871291	MT307599
DQ422948	KC187611	MH000304	MK749280	MN871292	MT307600
DQ435682	KC187627	MH000305	MK749281	MN871293	MT307601
DQ435683	KC187662	MH000306	MK749282	MN871294	MT307602
DQ435684	KC187699	MH000307	MK749283	MN871295	MT307603
DQ444258	KC247375	MH000308	MK749284	MN871296	MT307604
DQ444260	KC247954	MH000309	MK749285	MN871297	MT307605
DQ445632	KC247955	MH000310	MK749286	MN871298	MT307606
DQ445633	KC247957	MH000311	MK749287	MN871299	MT307607
DQ445634	KC247962	MH000312	MK749288	MN871300	MT307608
DQ445635	KC247963	MH000313	MK749289	MN871301	MT307609
DQ445637	KC247978	MH000314	MK749290	MN871302	MT307610
DQ447266	KC247981	MH012257	MK749291	MN871303	MT307611
DQ447267	KC247983	MH012290	MK749292	MN871304	MT307612
DQ447268	KC247984	MH012316	MK749293	MN871305	MT307613
DQ447269	KC247985	MH012359	MK749294	MN871306	MT307614
DQ447270	KC247987	MH012394	MK749295	MN871307	MT307615
DQ447272	KC247989	MH012421	MK749296	MN871308	MT307616
DQ448819	KC247992	MH012462	MK757421	MN871309	MT307617
DQ487188	KC247993	MH012490	MK790783	MN871310	MT307618
DQ487190	KC247994	MH012519	MK790784	MN871311	MT307619
DQ518410	KC247997	MH012541	MK790785	MN871312	MT307620
DQ518411	KC248001	MH012580	MK790786	MN871313	MT307621
DQ518412	KC248002	MH012611	MK790787	MN871314	MT307622
DQ672623	KC248004	MH012636	MK790788	MN871315	MT307623
DQ676870	KC248008	MH012660	MK790789	MN871316	MT307624
DQ676872	KC248011	MH012678	MK790790	MN871317	MT307625
DQ676874	KC248012	MH012718	MK790791	MN871318	MT307626
DQ676877	KC248013	MH012772	MK790792	MN871319	MT307627
DQ676880	KC248015	MH012798	MK790793	MN871320	MT307628

DQ676883	KC248017	MH012806	MK790794	MN871321	MT307629
DQ676886	KC248021	MH012824	MK790795	MN871322	MT307630
DQ789392	KC248024	MH012859	MK790796	MN871323	MT307631
DQ823356	KC248026	MH012891	MK790797	MN871324	MT307632
DQ823357	KC248027	MH012923	MK790798	MN871325	MT307633
DQ823358	KC248032	MH012945	MK790799	MN871326	MT307634
DQ823359	KC248034	MH012963	MK790800	MN871327	MT307635
DQ823360	KC248035	MH012994	MK790801	MN871328	MT307636
DQ823361	KC248036	MH013027	MK790802	MN871329	MT307637
DQ823362	KC462190	MH013054	MK790803	MN871330	MT307638
DQ823363	KC462191	MH013075	MK790804	MN871331	MT307639
DQ823364	KC473824	MH013090	MK790805	MN871332	MT307640
DQ823365	KC473825	MH013129	MK790806	MN871333	MT307641
DQ823366	KC473826	MH013130	MK790807	MN887608	MT307642
DQ823367	KC473827	MH013132	MK790808	MN887609	MT307643
DQ826726	KC473828	MH013133	MK790809	MN887611	MT307644
DQ826727	KC473829	MH013134	MK790810	MN887612	MT307645
DQ837381	KC473830	MH013135	MK790811	MN887613	MT307646
DQ845386	KC473831	MH013136	MK790812	MN887614	MT307647
DQ845387	KC473832	MH013138	MK790813	MN887615	MT307648
DQ845388	KC473833	MH013139	MK790814	MN887616	MT307649
DQ853427	KC473834	MH013140	MK790815	MN887619	MT307650
DQ853455	KC473835	MH013141	MK790816	MN887621	MT307651
DQ859178	KC473836	MH013142	MK790817	MN887622	MT307652
DQ859179	KC473841	MH013143	MK790818	MN944109	MT307653
DQ859180	KC473845	MH013144	MK790819	MN944110	MT307654
DQ869014	KC473846	MH013145	MK790820	MN944111	MT307655
DQ869015	KC492737	MH013146	MK790821	MN944112	MT307656
DQ869016	KC522031	MH013147	MK790822	MN944113	MT307657
DQ869017	KC522032	MH013148	MK790823	MN944114	MT307658
DQ869018	KC522033	MH013149	MK790824	MN944115	MT307659
DQ869019	KC522034	MH013150	MK790825	MN944116	MT307660
DQ869020	KC522035	MH013151	MK790826	MN944117	MT307661
DQ869021	KC595149	MH013152	MK790827	MN944118	MT307662
DQ869022	KC595150	MH013153	MK790828	MN944119	MT307663
DQ869023	KC595151	MH013154	MK790829	MN944120	MT307664
DQ869024	KC595152	MH013155	MK790830	MN944121	MT307665
DQ869025	KC595153	MH013156	MK790831	MN944122	MT307666
DQ869026	KC595154	MH013157	MK790832	MN944123	MT307667
DQ869027	KC595155	MH013158	MK790833	MN944124	MT307668
DQ869028	KC595156	MH013159	MK790834	MN944125	MT307669
DQ869029	KC595157	MH013160	MK790835	MN944126	MT307670
DQ869030	KC595158	MH013161	MK790836	MN944127	MT307671
DQ869031	KC595159	MH013162	MK790837	MN944128	MT307672
DQ869032	KC595160	MH013163	MK790838	MN944129	MT307673
DQ869033	KC595161	MH013164	MK790839	MN944130	MT307674
DQ886031	KC595162	MH013165	MK790840	MN944131	MT307675

DQ886032	KC595163	MH013166	MK790841	MN944132	MT307676
DQ886033	KC595164	MH013167	MK790842	MN944133	MT307677
DQ886034	KC595165	MH013168	MK790843	MN944134	MT307678
DQ886035	KC595166	MH013170	MK790844	MN944135	MT307679
DQ886036	KC595167	MH013171	MK790845	MN944136	MT307680
DQ886037	KC595168	MH013172	MK790846	MN944138	MT307681
DQ886038	KC595169	MH013173	MK790847	MN944139	MT307682
DQ890515	KC595170	MH013174	MK790848	MN944141	MT307683
DQ912822	KC595171	MH013175	MK790849	MN944142	MT307684
DQ912823	KC595172	MH013176	MK790850	MN944143	MT307685
DQ926899	KC595173	MH013177	MK790851	MN944144	MT307686
DQ976394	KC595174	MH013178	MK790852	MN944145	MT307687
DQ979023	KC595175	MH013179	MK790853	MN944146	MT307688
DQ979024	KC595177	MH013180	MK790854	MN944147	MT307689
DQ979025	KC595178	MH013181	MK790855	MN944148	MT307690
DQ990880	KC595182	MH013182	MK790856	MN944149	MT307691
EF025168	KC595183	MH013183	MK790857	MN944150	MT307692
EF025323	KC595184	MH013184	MK790858	MN944151	MT307693
EF029066	KC595186	MH013185	MK790859	MN944152	MT307694
EF033657	KC595189	MH013186	MK790860	MN944153	MT307695
EF033659	KC595194	MH013187	MK790861	MN944154	MT307696
EF036527	KC595195	MH029899	MK790862	MN944155	MT307697
EF036528	KC595196	MH060263	MK790863	MN944156	MT307698
EF036529	KC595197	MH060484	MK790864	MN944157	MT307699
EF036530	KC595201	MH060485	MK790865	MN944158	MT307700
EF036531	KC595202	MH060614	MK790866	MN944159	MT307701
EF036532	KC595203	MH060630	MK790867	MN944160	MT307702
EF036533	KC595204	MH060681	MK790868	MN944161	MT307703
EF036534	KC595206	MH060746	MK790869	MN944162	MT307704
EF036535	KC595208	MH060766	MK790870	MN944163	MT307705
EF036536	KC596064	MH060814	MK790871	MN944164	MT307706
EF042692	KC596065	MH060823	MK790872	MN944165	MT307707
EF087994	KC596066	MH060884	MK790873	MN944166	MT307708
EF087995	KC596067	MH060990	MK790874	MN944167	MT307709
EF091932	KC596068	MH078530	MK790875	MN944168	MT307710
EF116594	KC634109	MH078531	MK790876	MN944169	MT307711
EF117254	KC634136	MH078532	MK790877	MN944170	MT307712
EF117255	KC634164	MH078533	MK790878	MN944171	MT307713
EF117256	KC634184	MH078535	MK790879	MN944172	MT307714
EF117257	KC699001	MH078536	MK790880	MN944173	MT307715
EF117258	KC699002	MH078537	MK790881	MN944174	MT307716
EF117259	KC699003	MH078538	MK790882	MN944175	MT307717
EF117260	KC699004	MH078539	MK790883	MN944176	MT307718
EF117261	KC699005	MH078541	MK790884	MN944177	MT307719
EF117262	KC699006	MH078542	MK790885	MN944178	MT307720
EF117263	KC699007	MH078543	MK790886	MN944179	MT307721
EF117264	KC699008	MH078544	MK790887	MN944180	MT307722

EF117265	KC699009	MH078545	MK790888	MN944181	MT307723
EF117266	KC699010	MH078546	MK790889	MN944182	MT307724
EF117267	KC699011	MH078547	MK790890	MN944183	MT307725
EF117268	KC699012	MH078548	MK790891	MN944184	MT307726
EF117269	KC699013	MH078549	MK790892	MN944185	MT307727
EF117270	KC699014	MH078550	MK790893	MN944186	MT307728
EF117271	KC699015	MH078551	MK790894	MN944187	MT307729
EF117272	KC699016	MH078552	MK790895	MN944188	MT307730
EF117273	KC699017	MH078553	MK790896	MN944189	MT307731
EF117274	KC699018	MH078554	MK790897	MN944190	MT307732
EF121405	KC699020	MH078555	MK790898	MN944191	MT307733
EF125655	KC699021	MH078556	MK790899	MN944192	MT307734
EF158040	KC699022	MH078557	MK790900	MN944193	MT307735
EF158041	KC699023	MH078559	MK790901	MN944194	MT307736
EF158042	KC699024	MH078560	MK790902	MN944195	MT307737
EF158043	KC699025	MH078561	MK790903	MN944196	MT307738
EF159970	KC699026	MH078562	MK790904	MN944197	MT307739
EF159971	KC699028	MH078563	MK790905	MN944198	MT307740
EF159972	KC699029	MH078564	MK790906	MN944199	MT307741
EF159973	KC699030	MH141491	MK790907	MN944200	MT307742
EF159974	KC699031	MH141492	MK790908	MN944201	MT307743
EF165539	KC699032	MH141493	MK790909	MN944202	MT307744
EF165540	KC699033	MH141494	MK790910	MN944203	MT307745
EF165541	KC699034	MH220193	MK790911	MN944204	MT307746
EF178338	KC699035	MH220194	MK790912	MN944205	MT307747
EF178354	KC699036	MH234639	MK790913	MN944206	MT307748
EF178358	KC699037	MH234640	MK790914	MN944207	MT307749
EF192591	KC699038	MH234641	MK790915	MN944208	MT307750
EF210725	KC699039	MH234642	MK790916	MN944209	MT307751
EF210726	KC699040	MH234643	MK790917	MN944210	MT307752
EF210727	KC748972	MH257525	MK790918	MN944211	MT307753
EF210729	KC749001	MH257526	MK790919	MN944212	MT307754
EF210730	KC749033	MH257527	MK790920	MN944213	MT307755
EF210731	KC797171	MH257528	MK790921	MN944214	MT307756
EF210732	KC797215	MH257529	MK790922	MN944215	MT307757
EF210734	KC807912	MH257530	MK790923	MN944216	MT307758
EF210735	KC807924	MH257531	MK790924	MN944217	MT307759
EF363122	KC807932	MH257532	MK790925	MN944218	MT307760
EF367173	KC807943	MH257533	MK790926	MN944219	MT307761
EF367208	KC807954	MH257534	MK790927	MN944220	MT307762
EF368370	KC807957	MH257535	MK790928	MN944221	MT307763
EF368371	KC807969	MH257536	MK790929	MN944222	MT307764
EF368372	KC807981	MH257537	MK790930	MN944223	MT307765
EF420986	KC833436	MH257538	MK790931	MN944224	MT307766
EF440689	KC834602	MH257539	MK790932	MN944225	MT307767
EF469243	KC834604	MH257540	MK790933	MN944226	MT307768
EF495062	KC852172	MH257541	MK790934	MN944227	MT307769

EF514697	KC852173	MH262588	MK790935	MN944228	MT307770
EF514698	KC852174	MH262625	MK790936	MN944229	MT307771
EF514699	KC862610	MH262644	MK790937	MN944230	MT307772
EF514700	KC862627	MH262665	MK790938	MN944231	MT307773
EF514701	KC862648	MH262688	MK790939	MN944232	MT307774
EF514702	KC862666	MH262722	MK790940	MN944233	MT307775
EF514703	KC862690	MH263122	MK790941	MN944234	MT307776
EF514704	KC862709	MH263194	MK790942	MN944235	MT307777
EF514705	KC862732	MH263749	MK790943	MN944236	MT307778
EF514706	KC862752	MH263882	MK790944	MN944237	MT307779
EF514707	KC862774	MH263982	MK790945	MN944238	MT307780
EF514708	KC862793	MH264031	MK790946	MN944239	MT307781
EF514709	KC862809	MH264099	MK790947	MN944240	MT307782
EF514710	KC862828	MH264149	MK790948	MN944241	MT307783
EF514711	KC862848	MH264237	MK790949	MN944242	MT307784
EF514712	KC862858	MH264326	MK790950	MN944243	MT307785
EF514713	KC862875	MH327744	MK790951	MN944244	MT307786
EF531330	KC862891	MH327745	MK790952	MN944245	MT307787
EF531331	KC862912	MH327746	MK790953	MN944246	MT307788
EF531333	KC862931	MH327747	MK790954	MN944247	MT307789
EF531335	KC862949	MH327748	MK790955	MN944248	MT307790
EF545108	KC862967	MH327749	MK790956	MN944249	MT307791
EF575354	KC862986	MH327750	MK790957	MN944250	MT307792
EF575370	KC863009	MH327752	MK790958	MN944251	MT307793
EF575384	KC863032	MH327753	MK790959	MN944252	MT307794
EF575400	KC863053	MH327754	MK790960	MN944253	MT307795
EF575414	KC863073	MH327755	MK790961	MN944254	MT307796
EF575429	KC863092	MH327756	MK790962	MN944255	MT307797
EF575444	KC863108	MH327757	MK790963	MN944256	MT307798
EF575455	KC863126	MH327758	MK790964	MN944257	MT307799
EF575471	KC863145	MH327759	MK790965	MN944258	MT307800
EF589039	KC863162	MH327760	MK790966	MN944259	MT307801
EF589040	KC863178	MH327761	MK790967	MN944260	MT307802
EF589041	KC863201	MH327762	MK790968	MN944261	MT307803
EF589042	KC863215	MH327763	MK790969	MN944262	MT307804
EF589043	KC863239	MH327764	MK790970	MN944263	MT307805
EF589044	KC863305	MH327765	MK790971	MN944264	MT307806
EF593181	KC863324	MH327766	MK790972	MN944265	MT307807
EF593182	KC863361	MH330337	MK790973	MN944266	MT307808
EF593186	KC863362	MH330338	MK790974	MN944267	MT307809
EF593187	KC863569	MH330339	MK790975	MN944268	MT307810
EF593188	KC863583	MH330340	MK790976	MN944269	MT307811
EF593189	KC863599	MH330341	MK790977	MN944270	MT307812
EF593190	KC863613	MH330342	MK790978	MN944271	MT307813
EF593191	KC863633	MH330343	MK790979	MN944272	MT307814
EF593192	KC870027	MH330344	MK790980	MN944273	MT307815
EF593193	KC870028	MH330345	MK790981	MN944274	MT307816

EF593194	KC870029	MH330346	MK790982	MN944275	MT307817
EF593195	KC870030	MH330347	MK790983	MN944276	MT307818
EF593196	KC870031	MH330348	MK790984	MN944277	MT307819
EF593197	KC870032	MH330349	MK790985	MN944278	MT307820
EF593198	KC870034	MH330350	MK790986	MN944279	MT307821
EF593199	KC870035	MH330351	MK790987	MN944280	MT307822
EF593200	KC870036	MH330352	MK790988	MN944281	MT307823
EF593201	KC870037	MH330353	MK790989	MN944282	MT307824
EF593202	KC870038	MH330354	MK790990	MN944283	MT307825
EF593203	KC870039	MH330355	MK790991	MN944284	MT307826
EF593204	KC870040	MH330356	MK790992	MN944285	MT307827
EF593205	KC870041	MH330357	MK790993	MN944286	MT307828
EF593206	KC870042	MH330358	MK790994	MN944287	MT307829
EF593207	KC870043	MH330359	MK790995	MN944288	MT307830
EF593208	KC870044	MH330361	MK790996	MN944289	MT307831
EF593209	KC894117	MH330363	MK790997	MN944290	MT307832
EF593210	KC894120	MH330364	MK790998	MN944291	MT307833
EF593211	KC894121	MH330365	MK790999	MN944292	MT307834
EF593212	KC894123	MH330367	MK791000	MN944293	MT307835
EF593213	KC894128	MH330370	MK791001	MN944294	MT307836
EF593216	KC894130	MH330371	MK791002	MN944295	MT307837
EF593217	KC894132	MH330372	MK791003	MN944296	MT307838
EF593218	KC894383	MH330373	MK791004	MN944297	MT307839
EF593219	KC898975	MH330374	MK791005	MN944299	MT307840
EF593220	KC898976	MH330375	MK791006	MN944300	MT307841
EF593221	KC898977	MH330376	MK791007	MN944301	MT307842
EF593222	KC898978	MH330377	MK791008	MN944302	MT307843
EF593223	KC898979	MH330378	MK791009	MN944303	MT307844
EF593224	KC898980	MH330379	MK791010	MN944304	MT307845
EF593225	KC898981	MH330380	MK791011	MN944305	MT307846
EF593226	KC898982	MH330381	MK791012	MN944306	MT307847
EF593227	KC898983	MH377336	MK791013	MN944307	MT307848
EF593228	KC898984	MH388438	MK791014	MN944308	MT307849
EF593229	KC898985	MH396608	MK791015	MN944309	MT307850
EF593231	KC898986	MH396609	MK791016	MN944310	MT307851
EF593232	KC898987	MH425161	MK791017	MN944311	MT307852
EF593233	KC898988	MH431770	MK791018	MN944312	MT307853
EF593234	KC898989	MH431788	MK791019	MN944313	MT307854
EF593238	KC898990	MH460464	MK791020	MN944315	MT307855
EF593240	KC898991	MH460465	MK791021	MN944316	MT307856
EF593243	KC898992	MH460466	MK791022	MN944317	MT307857
EF593245	KC898993	MH460467	MK791023	MN944318	MT307858
EF593253	KC898994	MH460468	MK791024	MN944319	MT307859
EF593254	KC898995	MH460469	MK791025	MN944320	MT307860
EF593256	KC898996	MH479275	MK791026	MN944321	MT307861
EF593259	KC899003	MH479933	MK791027	MN944322	MT307862
EF593260	KC899004	MH479962	MK791028	MN944323	MT307863

EF593261	KC899005	MH575375	MK791029	MN944324	MT307864
EF593262	KC899006	MH575478	MK791030	MN944325	MT307865
EF593263	KC899007	MH575546	MK791031	MN944326	MT307866
EF593264	KC899008	MH575582	MK791032	MN944327	MT307867
EF593265	KC899009	MH575694	MK791033	MN944328	MT307868
EF593266	KC899010	MH575724	MK791034	MN944329	MT307869
EF593267	KC899011	MH575774	MK791035	MN944330	MT307870
EF593268	KC899012	MH575856	MK791036	MN944331	MT307871
EF593269	KC899013	MH575922	MK791037	MN944332	MT307872
EF593270	KC899014	MH576039	MK791038	MN944333	MT307873
EF593271	KC899015	MH576077	MK791039	MN944334	MT307874
EF593272	KC899079	MH576107	MK791040	MN944335	MT307875
EF593273	KC899080	MH576152	MK791041	MN944336	MT307876
EF593274	KC899081	MH576253	MK791042	MN944337	MT307877
EF593275	KC911635	MH576325	MK791043	MN944338	MT307878
EF593276	KC914396	MH603967	MK791044	MN944339	MT307879
EF593277	KC935957	MH604056	MK791045	MN944340	MT307880
EF593279	KC935958	MH604101	MK791046	MN944341	MT307881
EF593281	KC935959	MH604118	MK791047	MN944342	MT307882
EF593282	KC990124	MH604219	MK791048	MN944343	MT307883
EF593283	KC990125	MH604303	MK791049	MN944344	MT307884
EF593284	KC990126	MH604308	MK791050	MN944345	MT307885
EF593285	KC990127	MH604392	MK791051	MN944346	MT307886
EF593286	KF011493	MH604408	MK791052	MN944347	MT307887
EF593287	KF011494	MH604484	MK791053	MN944348	MT307888
EF593288	KF011495	MH604492	MK791054	MN944349	MT308411
EF593289	KF061031	MH604507	MK791055	MN944350	MT308412
EF593290	KF061032	MH604522	MK791056	MN944351	MT347589
EF593291	KF114881	MH604535	MK791057	MN944352	MT347590
EF593292	KF114882	MH615836	MK791058	MN944353	MT347591
EF593293	KF114883	MH632763	MK791059	MN944354	MT347592
EF593294	KF114884	MH632822	MK791060	MN944355	MT347593
EF593295	KF114885	MH632949	MK791061	MN944356	MT347594
EF593296	KF114886	MH632956	MK791062	MN944357	MT347595
EF593297	KF114887	MH633031	MK791063	MN944358	MT347596
EF593298	KF114888	MH633155	MK791064	MN944359	MT347597
EF593299	KF114889	MH633199	MK791065	MN944360	MT347678
EF593300	KF114890	MH654975	MK791066	MN944361	MT347679
EF593301	KF114891	MH654976	MK791067	MN944362	MT347680
EF593302	KF114892	MH654977	MK791068	MN944363	MT347681
EF593303	KF114893	MH654978	MK791069	MN944364	MT366192
EF593304	KF114894	MH654979	MK791070	MN944365	MT366193
EF593305	KF234628	MH654980	MK791071	MN944366	MT366194
EF593306	KF250366	MH654981	MK791072	MN944367	MT366195
EF593308	KF250368	MH654982	MK791073	MN944368	MT366196
EF593309	KF250369	MH654983	MK791074	MN944369	MT366197
EF593310	KF250371	MH654984	MK791075	MN944370	MT395382

EF593311	KF250372	MH666154	MK791076	MN944371	MT395383
EF593312	KF250373	MH666155	MK791077	MN944372	MT395384
EF593313	KF250374	MH666156	MK791078	MN944373	MT395385
EF593314	KF250375	MH666157	MK791079	MN944374	MT395386
EF593315	KF250376	MH666158	MK791080	MN944375	MT395387
EF593316	KF250377	MH666161	MK791081	MN944376	MT395388
EF593317	KF250378	MH666162	MK791082	MN944377	MT395389
EF593318	KF250379	MH666165	MK791083	MN944378	MT395390
EF593319	KF250380	MH666166	MK791084	MN944379	MT395391
EF614151	KF250381	MH666167	MK791085	MN944380	MT395392
EF633445	KF250382	MH666168	MK791086	MN944381	MT395393
EF637046	KF250383	MH666169	MK791087	MN944382	MT395394
EF637047	KF250384	MH666170	MK791088	MN944383	MT395395
EF637048	KF250385	MH666171	MK791089	MN944384	MT395396
EF637049	KF250395	MH666172	MK791090	MN989412	MT395397
EF637050	KF250397	MH666173	MK791091	MN989924	MT395398
EF637051	KF250398	MH666174	MK791092	MN989925	MT395399
EF637052	KF250399	MH666175	MK791093	MN989926	MT395400
EF637053	KF250400	MH666176	MK791094	MN989927	MT395401
EF637054	KF250401	MH666177	MK791095	MT007547	MT395402
EF637055	KF250402	MH666178	MK791096	MT007548	MT395403
EF637056	KF250403	MH666179	MK791097	MT007549	MT395404
EF637057	KF250404	MH666180	MK791098	MT007550	MT395405
EF643652	KF250405	MH666181	MK791099	MT007551	MT395406
EF643656	KF250406	MH666182	MK791100	MT007552	MT395407
EF643659	KF250407	MH666183	MK791101	MT007553	MT395408
EF643662	KF250408	MH666184	MK791102	MT007554	MT395409
EF694032	KF250409	MH666185	MK791103	MT007555	MT395410
EF694034	KF250410	MH666186	MK791104	MT007556	MT395411
EF694037	KF268035	MH666187	MK791105	MT007557	MT395412
EU000506	KF268036	MH666188	MK791106	MT007558	MT395413
EU000509	KF268037	MH666189	MK791107	MT007559	MT395414
EU000512	KF268038	MH666190	MK791108	MT007560	MT395415
EU000515	KF268039	MH666191	MK791109	MT007561	MT395416
EU023922	KF268040	MH666192	MK791110	MT007562	MT395417
EU023929	KF268041	MH666193	MK791111	MT007563	MT395418
EU023932	KF268042	MH666194	MK791112	MT007564	MT395419
EU023933	KF268043	MH666195	MK791113	MT007565	MT395420
EU031913	KF268044	MH666196	MK791114	MT007566	MT395421
EU031914	KF268045	MH666197	MK791115	MT007567	MT395422
EU031915	KF268046	MH666198	MK791116	MT007568	MT395423
EU110086	KF268047	MH666199	MK791117	MT007569	MT395424
EU110087	KF268048	MH666200	MK791118	MT007570	MT395425
EU110088	KF384798	MH666201	MK791119	MT007571	MT395426
EU110089	KF384801	MH666202	MK791120	MT007572	MT395427
EU110090	KF384802	MH666203	MK791121	MT007573	MT395428
EU110091	KF384803	MH666204	MK791122	MT007574	MT395429

EU110092	KF384804	MH666205	MK791123	MT007575	MT395430
EU110093	KF384805	MH666206	MK791124	MT007576	MT395431
EU110094	KF384806	MH666207	MK791125	MT007577	MT395432
EU131787	KF384807	MH666208	MK791126	MT007578	MT395433
EU131788	KF384808	MH666209	MK791127	MT007579	MT395434
EU131789	KF384809	MH666210	MK791128	MT007580	MT395435
EU131790	KF384810	MH666211	MK791129	MT007581	MT395436
EU131791	KF384811	MH666212	MK791130	MT007582	MT395437
EU131792	KF384812	MH666214	MK791131	MT007583	MT395438
EU131793	KF384813	MH666215	MK791132	MT023027	MT395439
EU131794	KF384814	MH666216	MK791133	MT023028	MT395440
EU131796	KF425293	MH666217	MK791134	MT023029	MT395441
EU131797	KF526120	MH666218	MK792287	MT023030	MT395443
EU131798	KF526174	MH666219	MK792288	MT023031	MT395444
EU131799	KF526228	MH666220	MK792289	MT023032	MT395445
EU131800	KF526261	MH666221	MK850457	MT023033	MT395447
EU131801	KF526298	MH666222	MK867524	MT033127	MT395448
EU131802	KF526318	MH666223	MK867525	MT033131	MT395449
EU131803	KF527081	MH666224	MK867526	MT033140	MT395450
EU131804	KF541292	MH666225	MK867527	MT033141	MT395451
EU131805	KF561435	MH666226	MK867528	MT033143	MT395452
EU131806	KF561443	MH666227	MK867529	MT033155	MT395453
EU131807	KF682427	MH666228	MK867530	MT033158	MT395454
EU131808	KF716464	MH666229	MK867531	MT033159	MT395455
EU131809	KF716465	MH666230	MK867532	MT033160	MT395456
EU131810	KF716466	MH666231	MK867533	MT033169	MT395457
EU131811	KF716467	MH666232	MK867534	MT033182	MT395458
EU161643	KF716468	MH666233	MK867535	MT033187	MT395459
EU166353	KF716469	MH666234	MK867536	MT033189	MT395460
EU166379	KF716470	MH666235	MK867537	MT033190	MT395461
EU166413	KF716471	MH666236	MK867538	MT033191	MT395462
EU166439	KF716472	MH666237	MK867539	MT033194	MT395463
EU166576	KF716473	MH666238	MK867540	MT033197	MT395464
EU166605	KF716474	MH666239	MK867541	MT033203	MT395465
EU166653	KF716475	MH666240	MK867542	MT033243	MT395466
EU166681	KF716476	MH666241	MK867543	MT033246	MT395467
EU166718	KF716477	MH666242	MK867554	MT033248	MT395468
EU166759	KF716478	MH666243	MK867555	MT033251	MT395469
EU166779	KF716479	MH666244	MK867556	MT033253	MT395470
EU170136	KF716480	MH666245	MK867557	MT033258	MT395471
EU170138	KF716481	MH666246	MK867558	MT033261	MT395472
EU170139	KF716482	MH666247	MK867559	MT033262	MT395473
EU170140	KF716483	MH666248	MK867560	MT033270	MT395474
EU170141	KF716484	MH666249	MK867561	MT033278	MT395475
EU170142	KF716485	MH666250	MK867562	MT033288	MT395476
EU170143	KF716486	MH666251	MK867563	MT033290	MT395477
EU170144	KF716487	MH666252	MK867564	MT033299	MT395478

EU170145	KF716488	MH666253	MK867565	MT033301	MT395479
EU170146	KF716489	MH666254	MK867566	MT033317	MT395480
EU170147	KF716490	MH666255	MK867567	MT033320	MT395481
EU170148	KF716494	MH666256	MK867568	MT033327	MT395482
EU170150	KF716495	MH666257	MK867569	MT033331	MT395483
EU170151	KF716496	MH666258	MK867570	MT033333	MT395484
EU170153	KF716497	MH666259	MK867571	MT033338	MT395485
EU170154	KF716498	MH666260	MK867572	MT033344	MT395486
EU170155	KF725883	MH666261	MK867573	MT033349	MT395487
EU191613	KF725884	MH666262	MK867584	MT033350	MT395488
EU191615	KF725885	MH666263	MK867585	MT033353	MT395489
EU191616	KF725886	MH666264	MK867586	MT033361	MT395490
EU191617	KF725887	MH666265	MK867587	MT033367	MT395491
EU191618	KF725888	MH666266	MK867588	MT033371	MT395492
EU220698	KF725889	MH666267	MK867589	MT033377	MT395493
EU281995	KF725890	MH666268	MK867590	MT033380	MT395494
EU281996	KF725891	MH666269	MK867591	MT033408	MT395495
EU281997	KF725892	MH666270	MK867592	MT033409	MT395496
EU281998	KF725895	MH666271	MK867593	MT033415	MT395497
EU281999	KF725896	MH666272	MK867594	MT033417	MT395498
EU289185	KF725897	MH666273	MK867595	MT033423	MT395499
EU289186	KF725900	MH666274	MK867596	MT033426	MT395500
EU289197	KF725901	MH666275	MK867597	MT033427	MT395501
EU293444	KF725902	MH667256	MK867598	MT033428	MT395502
EU293450	KF725903	MH672692	MK867599	MT033430	MT395503
EU363825	KF725905	MH672693	MK867600	MT033432	MT395504
EU363826	KF725906	MH672694	MK867601	MT033438	MT395505
EU363827	KF725908	MH672695	MK867602	MT033446	MT395506
EU363828	KF725909	MH672696	MK867603	MT033447	MT395507
EU363829	KF725910	MH672697	MK867604	MT033458	MT395508
EU363830	KF725911	MH672698	MK867605	MT033461	MT395509
EU363831	KF725912	MH672699	MK867606	MT033465	MT395510
EU363832	KF725913	MH672700	MK867607	MT033475	MT395511
EU363833	KF725914	MH672701	MK867608	MT033479	MT417737
EU363834	KF725915	MH672702	MK867609	MT033482	MT417738
EU363835	KF725916	MH672703	MK867610	MT033493	MT417739
EU363836	KF725917	MH672704	MK867611	MT033504	MT417740
EU363837	KF725918	MH672705	MK867612	MT033510	MT417741
EU363838	KF725920	MH672706	MK867613	MT033513	MT417742
EU363839	KF725921	MH672707	MK867614	MT033520	MT417743
EU363840	KF725922	MH672708	MK867615	MT033524	MT417744
EU363841	KF725924	MH672709	MK867626	MT033527	MT417745
EU363842	KF725926	MH672710	MK867627	MT033528	MT417746
EU363843	KF725927	MH672711	MK867628	MT033547	MT417747
EU363844	KF725928	MH672712	MK867629	MT033549	MT417748
EU363845	KF725929	MH672713	MK867630	MT033573	MT417749
EU363846	KF725930	MH672714	MK867631	MT033575	MT417750

EU363847	KF725931	MH672715	MK867632	MT033595	MT417751
EU363848	KF725933	MH672716	MK867633	MT033596	MT417752
EU363849	KF725934	MH672717	MK867634	MT033597	MT417753
EU363850	KF725935	MH672718	MK867635	MT033628	MT417754
EU363851	KF725936	MH672719	MK867636	MT033636	MT417755
EU446022	KF725937	MH672720	MK867637	MT033639	MT417756
EU448295	KF725938	MH672721	MK867638	MT033641	MT417757
EU448296	KF725939	MH672722	MK867639	MT033642	MT417758
EU513182	KF725942	MH672723	MK867640	MT033647	MT417759
EU513183	KF725943	MH672724	MK867641	MT033651	MT417760
EU513184	KF725944	MH672725	MK867643	MT033656	MT417761
EU513185	KF725945	MH672726	MK867644	MT033672	MT417762
EU513186	KF725946	MH672727	MK867645	MT033675	MT417763
EU513187	KF725948	MH672728	MK871374	MT033686	MT417764
EU513188	KF725949	MH672729	MK984159	MT033688	MT417765
EU513189	KF725950	MH672730	MK984160	MT033699	MT417766
EU513190	KF725952	MH672731	MN043576	MT033701	MT417767
EU513191	KF725953	MH672732	MN043577	MT033713	MT417768
EU513192	KF725954	MH672733	MN043579	MT033716	MT417769
EU513193	KF725955	MH672734	MN043580	MT033717	MT417770
EU513194	KF725956	MH672735	MN043581	MT033719	MT417771
EU513195	KF725957	MH672736	MN043582	MT033720	MT417772
EU513196	KF725958	MH672737	MN043583	MT033721	MT419970
EU513198	KF725959	MH672738	MN043584	MT033726	MT419971
EU513199	KF725960	MH672739	MN043585	MT033727	MT419972
EU521727	KF725961	MH672740	MN043586	MT033728	MT419973
EU521728	KF725962	MH672741	MN043587	MT033729	MT419974
EU521729	KF725963	MH672742	MN043588	MT033730	MT419975
EU575734	KF725964	MH672743	MN043589	MT033732	MT419976
EU577480	KF725966	MH672744	MN043590	MT033734	MT419977
EU577589	KF725967	MH672745	MN043591	MT033735	MT419978
EU581823	KF725968	MH672746	MN043592	MT033739	MT419979
EU581824	KF725969	MH672747	MN043593	MT033761	MT419980
EU581825	KF725970	MH672748	MN043594	MT033776	MT419981
EU581826	KF725971	MH672749	MN043595	MT033799	MT419982
EU581827	KF725972	MH672750	MN043596	MT033811	MT419983
EU581828	KF725974	MH672751	MN043597	MT033827	MT419984
EU604549	KF725975	MH672752	MN043598	MT033840	MT419985
EU604591	KF725976	MH672753	MN043599	MT033853	MT419986
EU616639	KF725977	MH672754	MN043600	MT033855	MT419987
EU663615	KF725979	MH672755	MN043601	MT033857	MT419988
EU668963	KF725980	MH672756	MN043602	MT033863	MT419989
EU683891	KF725981	MH672757	MN043603	MT033865	MT419990
EU693240	KF725982	MH672759	MN043604	MT033866	MT419991
EU697904	KF725983	MH672760	MN043605	MT033867	MT419992
EU697905	KF725984	MH672762	MN043606	MT033868	MT419993
EU697906	KF725985	MH672763	MN043607	MT033870	MT419994

EU697907	KF725986	MH672764	MN044632	MT033875	MT419995
EU697908	KF725987	MH672765	MN055643	MT033876	MT419996
EU697909	KF725988	MH672766	MN055644	MT033877	MT419997
EU735534	KF725989	MH672767	MN067222	MT033878	MT419998
EU735535	KF725990	MH672768	MN067223	MT033879	MT419999
EU735536	KF725994	MH672769	MN067224	MT033880	MT420000
EU735537	KF725995	MH672770	MN067522	MT033881	MT420001
EU735538	KF725996	MH672771	MN090188	MT033882	MT420002
EU735539	KF725997	MH672772	MN090189	MT033883	MT420003
EU735540	KF725998	MH672773	MN090190	MT033884	MT420004
EU743757	KF725999	MH672774	MN090191	MT033885	MT420005
EU743758	KF726000	MH672775	MN090192	MT033886	MT420006
EU743759	KF726001	MH672776	MN090193	MT033887	MT420007
EU743760	KF726002	MH672777	MN090194	MT033888	MT420008
EU743761	KF726003	MH672778	MN090195	MT033889	MT420009
EU743762	KF726004	MH672779	MN090197	MT033890	MT420010
EU743763	KF726008	MH672780	MN090200	MT033891	MT420011
EU743764	KF726009	MH672781	MN090203	MT033892	MT420012
EU743765	KF726010	MH672782	MN090205	MT033893	MT420013
EU743766	KF726011	MH672783	MN090207	MT033894	MT420014
EU743767	KF726013	MH672784	MN090209	MT033895	MT420015
EU743768	KF726014	MH672785	MN090213	MT033896	MT420016
EU743769	KF726015	MH672786	MN090215	MT033897	MT420017
EU743770	KF726016	MH672787	MN090217	MT033898	MT420018
EU743771	KF726017	MH672788	MN090218	MT033899	MT420019
EU743772	KF726018	MH672789	MN090221	MT033900	MT420020
EU743773	KF726020	MH672790	MN090222	MT033901	MT420021
EU743774	KF726021	MH672791	MN090224	MT033902	MT420022
EU743775	KF726022	MH672792	MN090227	MT033903	MT420023
EU743776	KF726023	MH672794	MN090229	MT033904	MT420024
EU743777	KF726025	MH672795	MN090231	MT033905	MT420025
EU743778	KF758551	MH672796	MN090233	MT033906	MT420026
EU743779	KF766537	MH672797	MN090234	MT101871	MT420027
EU743780	KF766540	MH672798	MN090241	MT121311	MT420028
EU743781	KF766541	MH672799	MN090242	MT121312	MT420029
EU743782	KF803580	MH672800	MN090243	MT121313	MT420030
EU743783	KF835493	MH672801	MN090245	MT121314	MT420031
EU743784	KF835494	MH672802	MN090246	MT121315	MT420032
EU743785	KF835495	MH672803	MN090247	MT121316	MT420033
EU743786	KF835496	MH672804	MN090252	MT121317	MT420034
EU743787	KF835497	MH672805	MN090254	MT121318	MT420035
EU743788	KF835499	MH672806	MN090255	MT121319	MT420036
EU743789	KF835500	MH672807	MN090256	MT121320	MT420037
EU743790	KF835501	MH672808	MN090257	MT121321	MT420038
EU743791	KF835502	MH672809	MN090261	MT121322	MT420039
EU743792	KF835503	MH672810	MN090262	MT121323	MT420040
EU743793	KF835504	MH672811	MN090264	MT121324	MT420041

EU743794	KF835505	MH672812	MN090265	MT121325	MT420042
EU743963	KF835506	MH672813	MN090266	MT121326	MT420043
EU743964	KF835507	MH672814	MN090268	MT121327	MT420044
EU743973	KF835508	MH672815	MN090269	MT121328	MT420045
EU744014	KF835509	MH672816	MN090270	MT121329	MT420046
EU744055	KF835510	MH672817	MN090271	MT121330	MT420047
EU744097	KF835511	MH672818	MN090272	MT121331	MT420048
EU744146	KF835512	MH672819	MN090274	MT121332	MT420049
EU786670	KF835513	MH672820	MN090275	MT121333	MT420050
EU786671	KF835514	MH672821	MN090276	MT121334	MT420051
EU786672	KF835515	MH672822	MN090277	MT121335	MT420052
EU786673	KF835516	MH672823	MN090279	MT121351	MT420053
EU786674	KF835517	MH672824	MN090280	MT121352	MT420054
EU786675	KF835518	MH672825	MN090281	MT121353	MT420055
EU786676	KF835519	MH672826	MN090282	MT121354	MT420056
EU786677	KF835520	MH672827	MN090287	MT121355	MT420057
EU786678	KF835521	MH672828	MN090289	MT121356	MT420058
EU786679	KF835522	MH672829	MN090290	MT121357	MT420059
EU786680	KF835523	MH672830	MN090291	MT121358	MT420060
EU786681	KF835524	MH672831	MN090292	MT121359	MT420061
EU807761	KF835525	MH672832	MN090293	MT121360	MT420062
EU839596	KF835526	MH672833	MN090295	MT121361	MT420063
EU839597	KF835527	MH672834	MN090297	MT121362	MT420064
EU839598	KF835528	MH672835	MN090298	MT121363	MT420065
EU839599	KF835529	MH672836	MN090299	MT121364	MT420066
EU839600	KF835530	MH672837	MN090300	MT121365	MT420067
EU839601	KF835531	MH672838	MN090301	MT121366	MT420068
EU839602	KF835532	MH672839	MN090306	MT121367	MT420069
EU839603	KF835533	MH672840	MN090308	MT121368	MT420070
EU839604	KF835534	MH672841	MN090309	MT121369	MT420071
EU839605	KF835535	MH672842	MN090310	MT121370	MT420072
EU839606	KF835536	MH672843	MN090312	MT121371	MT420073
EU839607	KF835537	MH672844	MN090313	MT121372	MT420074
EU839608	KF835538	MH672845	MN090315	MT121373	MT420075
EU839609	KF835539	MH672846	MN090316	MT121374	MT420076
EU839610	KF835540	MH672847	MN090319	MT121375	MT420077
EU850429	KF835541	MH672848	MN090321	MT121401	MT420078
EU852934	KF835542	MH672849	MN090322	MT121402	MT420079
EU852942	KF835543	MH672850	MN090324	MT121403	MT420080
EU852950	KF835544	MH672851	MN090325	MT121404	MT420081
EU852958	KF835545	MH672852	MN090326	MT121405	MT420082
EU852966	KF835546	MH672853	MN090327	MT121406	MT420083
EU852974	KF835547	MH672854	MN090328	MT121407	MT420084
EU852982	KF850149	MH672855	MN090329	MT121408	MT420085
EU852990	KF859741	MH672856	MN090330	MT121409	MT420086
EU852998	KF859742	MH672857	MN090332	MT121410	MT420087
EU853006	KF859743	MH672858	MN090333	MT121411	MT420088

EU853014	KF859744	MH672859	MN090334	MT121412	MT420089
EU853022	KF859745	MH672860	MN090335	MT121413	MT420090
EU853030	KF859746	MH672861	MN090336	MT121414	MT420091
EU853038	KF859747	MH672862	MN090337	MT121415	MT420092
EU853046	KF859773	MH672863	MN090338	MT121416	MT420093
EU853054	KF927150	MH672864	MN090340	MT121417	MT420094
EU853062	KF927151	MH672865	MN090341	MT121418	MT420095
EU853070	KF985982	MH672866	MN090343	MT121419	MT420096
EU853078	KF985994	MH672867	MN090344	MT121420	MT420097
EU853086	KF986006	MH672868	MN090345	MT121421	MT420098
EU853094	KF986021	MH672869	MN090346	MT121422	MT420099
EU853102	KF986033	MH672870	MN090347	MT121423	MT420100
EU853110	KF986041	MH672871	MN090348	MT121424	MT420101
EU853118	KF986050	MH672872	MN090349	MT121425	MT420102
EU853126	KF986058	MH672873	MN090350	MT121441	MT420103
EU853134	KF986066	MH672874	MN090351	MT121442	MT420104
EU861977	KF986074	MH672875	MN090352	MT121444	MT420105
EU884500	KF986082	MH672876	MN090353	MT121445	MT420106
EU884501	KF986090	MH672877	MN090354	MT121446	MT420107
EU885759	KF986098	MH672878	MN090355	MT121447	MT420108
EU885760	KF986106	MH672879	MN090356	MT121448	MT420109
EU885761	KF986114	MH672880	MN090357	MT121449	MT420110
EU885762	KF986123	MH672881	MN090358	MT121450	MT420111
EU885763	KF986131	MH672882	MN090359	MT121451	MT420112
EU885764	KF986139	MH672883	MN090361	MT121452	MT420113
EU885765	KF990605	MH672884	MN090362	MT121453	MT420114
EU885766	KF990608	MH672885	MN090364	MT121454	MT420115
EU908218	KJ019215	MH672886	MN090365	MT121455	MT420116
FJ152546	KJ140245	MH672887	MN090366	MT121456	MT420117
FJ152547	KJ140247	MH672888	MN090367	MT121457	MT420118
FJ152548	KJ140248	MH672889	MN090368	MT121458	MT420119
FJ185228	KJ140249	MH672890	MN090369	MT121459	MT420120
FJ185229	KJ140250	MH672891	MN090370	MT121460	MT420121
FJ185230	KJ140251	MH672892	MN090371	MT121461	MT420122
FJ185231	KJ140252	MH672893	MN090372	MT121462	MT420123
FJ185232	KJ140255	MH672894	MN090373	MT121463	MT420124
FJ185233	KJ140256	MH672895	MN090374	MT121464	MT420125
FJ185234	KJ140257	MH672896	MN090375	MT121465	MT420126
FJ185235	KJ140259	MH672897	MN090376	MT121491	MT420127
FJ185236	KJ140260	MH672898	MN090377	MT121492	MT420128
FJ185237	KJ140261	MH672899	MN090378	MT121493	MT420129
FJ185238	KJ140262	MH672900	MN090379	MT121494	MT420130
FJ185239	KJ140264	MH672901	MN090380	MT121495	MT420131
FJ185240	KJ140265	MH672902	MN090381	MT121496	MT420132
FJ185241	KJ140266	MH672903	MN090382	MT121497	MT420133
FJ185242	KJ140267	MH672904	MN090383	MT121498	MT420134
FJ185243	KJ158421	MH672905	MN090384	MT121499	MT420135

FJ185244	KJ158422	MH672906	MN090385	MT121500	MT420136
FJ185245	KJ158423	MH672907	MN090386	MT121501	MT420137
FJ185246	KJ158424	MH672908	MN090387	MT121502	MT420138
FJ185247	KJ158425	MH672909	MN090388	MT121503	MT420139
FJ185248	KJ158426	MH672910	MN090389	MT121504	MT420140
FJ185249	KJ158427	MH672911	MN090390	MT121505	MT420141
FJ185250	KJ158428	MH672912	MN090392	MT121506	MT420142
FJ185251	KJ158429	MH672913	MN090393	MT121507	MT420143
FJ185252	KJ158431	MH672914	MN090394	MT121508	MT420144
FJ185253	KJ158432	MH672915	MN090395	MT121509	MT420145
FJ185254	KJ158433	MH672916	MN090396	MT121510	MT420146
FJ185255	KJ158434	MH672917	MN090397	MT121511	MT420147
FJ185256	KJ158435	MH672918	MN090399	MT121512	MT420148
FJ185257	KJ158436	MH672919	MN090400	MT121513	MT420149
FJ185258	KJ158437	MH672920	MN090401	MT121514	MT420150
FJ185259	KJ158438	MH672921	MN090402	MT121515	MT420151
FJ185260	KJ158439	MH672922	MN090403	MT121537	MT420152
FJ195086	KJ158440	MH672923	MN090404	MT121538	MT420153
FJ195088	KJ184176	MH672924	MN090405	MT121539	MT420154
FJ195089	KJ184177	MH672927	MN090407	MT121540	MT420155
FJ195090	KJ184180	MH672928	MN090408	MT121541	MT420156
FJ195091	KJ197200	MH672929	MN090409	MT121542	MT420157
FJ213780	KJ197201	MH672930	MN090410	MT121543	MT420158
FJ213781	KJ197202	MH672931	MN090411	MT121544	MT420159
FJ213782	KJ206289	MH672932	MN090412	MT121545	MT420160
FJ213783	KJ484433	MH672933	MN090414	MT121546	MT420161
FJ238521	KJ484434	MH672934	MN090415	MT121547	MT420162
FJ358521	KJ484435	MH672935	MN090416	MT121548	MT420163
FJ388890	KJ485697	MH672936	MN090417	MT121549	MT420164
FJ388891	KJ485698	MH672937	MN090418	MT121550	MT420165
FJ388892	KJ541837	MH672938	MN090419	MT121551	MT420166
FJ388893	KJ541839	MH672939	MN090420	MT121552	MT420167
FJ388894	KJ541840	MH672940	MN090421	MT121553	MT420168
FJ388895	KJ541841	MH672941	MN090422	MT121554	MT420169
FJ388896	KJ541842	MH672942	MN090423	MT121555	MT420170
FJ388898	KJ541843	MH672943	MN090424	MT121571	MT420171
FJ388899	KJ541844	MH672945	MN090425	MT121572	MT420172
FJ388901	KJ541845	MH672946	MN090426	MT121573	MT420173
FJ388902	KJ541846	MH672947	MN090427	MT121574	MT420174
FJ388903	KJ541847	MH672948	MN090428	MT121575	MT420175
FJ388904	KJ541848	MH672949	MN090429	MT121576	MT420176
FJ388905	KJ541849	MH672950	MN090430	MT121577	MT420177
FJ388906	KJ541850	MH672951	MN090431	MT121578	MT420178
FJ388908	KJ541851	MH672952	MN090432	MT121579	MT420179
FJ388909	KJ579955	MH672953	MN090433	MT121580	MT420180
FJ388910	KJ671533	MH672954	MN090434	MT121581	MT420181
FJ388911	KJ671534	MH672955	MN090436	MT121582	MT420182

FJ388912	KJ671535	MH672956	MN090438	MT121583	MT420183
FJ388913	KJ671536	MH672957	MN090439	MT121584	MT420184
FJ388914	KJ671537	MH672958	MN090440	MT121585	MT420185
FJ388915	KJ698244	MH672959	MN090441	MT121586	MT420186
FJ388916	KJ704789	MH672960	MN090442	MT121587	MT420187
FJ388917	KJ704790	MH672961	MN090443	MT121588	MT420188
FJ388918	KJ704791	MH672962	MN090444	MT121589	MT420189
FJ388919	KJ704792	MH672963	MN090445	MT121590	MT420190
FJ388920	KJ704793	MH672964	MN090446	MT121591	MT420191
FJ388921	KJ704794	MH672965	MN090447	MT121592	MT420192
FJ388923	KJ704795	MH672966	MN090448	MT121593	MT420193
FJ388924	KJ769147	MH672967	MN090449	MT121594	MT420194
FJ388925	KJ778895	MH672968	MN090450	MT121595	MT420195
FJ388927	KJ778896	MH672969	MN090451	MT121611	MT420196
FJ388928	KJ778897	MH672970	MN090452	MT121612	MT420197
FJ388929	KJ787683	MH672971	MN090453	MT121613	MT420198
FJ388930	KJ787684	MH672972	MN090454	MT121614	MT420199
FJ388931	KJ849757	MH672973	MN090455	MT121615	MT420200
FJ388932	KJ849758	MH672974	MN090456	MT121616	MT420201
FJ388933	KJ849759	MH672975	MN090457	MT121617	MT420202
FJ388934	KJ849760	MH672976	MN090458	MT121618	MT420203
FJ388935	KJ849761	MH672977	MN090459	MT121619	MT420204
FJ388936	KJ849762	MH672978	MN090460	MT121620	MT420205
FJ388937	KJ849763	MH672979	MN090461	MT121621	MT420206
FJ388938	KJ849767	MH672980	MN090462	MT121622	MT420207
FJ388939	KJ849768	MH672981	MN090463	MT121623	MT420208
FJ388940	KJ849769	MH672982	MN090464	MT121624	MT420209
FJ388941	KJ849770	MH672983	MN090465	MT121625	MT420210
FJ388942	KJ849771	MH672985	MN090466	MT121626	MT420211
FJ388943	KJ849773	MH672986	MN090467	MT121627	MT420212
FJ388944	KJ849774	MH672987	MN090468	MT121628	MT420213
FJ388945	KJ849775	MH672988	MN090469	MT121629	MT420214
FJ388946	KJ849776	MH672989	MN090470	MT121630	MT420215
FJ388947	KJ849777	MH672990	MN090471	MT121631	MT420216
FJ388948	KJ849778	MH672991	MN090472	MT121632	MT420217
FJ388949	KJ849779	MH672992	MN090473	MT121633	MT420218
FJ388950	KJ849780	MH672993	MN090474	MT121634	MT420219
FJ388951	KJ849782	MH672994	MN090475	MT121635	MT420220
FJ388952	KJ849784	MH672995	MN090476	MT121651	MT420221
FJ388953	KJ849785	MH672996	MN090477	MT121652	MT420222
FJ388954	KJ849786	MH672997	MN090478	MT121653	MT420223
FJ388955	KJ849787	MH672998	MN090479	MT121654	MT420224
FJ388956	KJ849788	MH672999	MN090480	MT121655	MT420225
FJ388957	KJ849789	MH673000	MN090481	MT121656	MT420226
FJ388958	KJ849790	MH673001	MN090482	MT121657	MT420227
FJ388959	KJ849791	MH673002	MN090483	MT121658	MT420228
FJ388960	KJ849793	MH673003	MN090484	MT121659	MT420229

FJ388961	KJ849794	MH673004	MN090485	MT121660	MT420230
FJ388962	KJ849795	MH673005	MN090486	MT121661	MT420231
FJ388963	KJ849796	MH673006	MN090487	MT121662	MT420232
FJ388964	KJ849798	MH673007	MN090488	MT121663	MT420233
FJ388965	KJ849799	MH673008	MN090489	MT121664	MT420234
FJ389363	KJ849800	MH673009	MN090490	MT121665	MT420235
FJ389364	KJ849801	MH673010	MN090491	MT121666	MT420236
FJ389365	KJ849802	MH673011	MN090492	MT121667	MT420237
FJ389366	KJ849803	MH673012	MN090493	MT121668	MT420238
FJ389367	KJ849804	MH673013	MN090494	MT121669	MT420239
FJ396012	KJ849805	MH673014	MN090495	MT121670	MT420240
FJ396016	KJ849807	MH673015	MN090496	MT121671	MT420241
FJ396022	KJ849808	MH673016	MN090497	MT121672	MT420242
FJ396026	KJ849809	MH673017	MN090498	MT121673	MT420243
FJ396028	KJ849810	MH673018	MN090499	MT121674	MT420244
FJ403482	KJ849811	MH673020	MN090500	MT121675	MT420245
FJ441290	KJ849812	MH673021	MN090501	MT121691	MT420247
FJ443128	KJ849813	MH673022	MN090502	MT121692	MT420248
FJ443140	KJ849814	MH673023	MN090503	MT121693	MT420249
FJ443159	KJ849815	MH673024	MN090504	MT121694	MT420250
FJ443166	KJ849816	MH673025	MN090505	MT121695	MT420251
FJ443167	KJ849817	MH673026	MN090506	MT121696	MT420252
FJ443177	KJ849818	MH673027	MN090507	MT121697	MT420253
FJ443196	KJ849820	MH673029	MN090508	MT121698	MT420254
FJ443209	KJ849821	MH673030	MN090509	MT121699	MT420255
FJ443215	KJ849824	MH682099	MN090510	MT121700	MT436112
FJ443222	KJ849825	MH683549	MN090511	MT121711	MT436113
FJ443240	KJ849826	MH683550	MN090512	MT121712	MT436114
FJ443253	KJ883137	MH684584	MN090513	MT121713	MT436115
FJ443274	KJ883138	MH705133	MN090514	MT121714	MT436116
FJ443356	KJ883139	MH705134	MN090515	MT121715	MT436117
FJ443367	KJ883142	MH705135	MN090516	MT121716	MT436118
FJ443379	KJ883143	MH705136	MN090517	MT121717	MT436119
FJ443455	KJ883144	MH705137	MN090518	MT121718	MT436120
FJ443474	KJ883145	MH705140	MN090519	MT121719	MT436121
FJ443520	KJ883146	MH705141	MN090520	MT121720	MT436122
FJ443533	KJ883147	MH705142	MN090521	MT121721	MT436123
FJ443548	KJ883148	MH705143	MN090522	MT121722	MT436124
FJ443557	KJ883149	MH705144	MN090523	MT121723	MT436125
FJ443575	KJ883150	MH705145	MN090524	MT121724	MT436126
FJ443600	KJ883151	MH705147	MN090525	MT121725	MT436127
FJ443624	KJ883152	MH705148	MN090526	MT121726	MT436128
FJ443644	KJ948656	MH705149	MN090527	MT121727	MT436129
FJ443666	KJ948657	MH705150	MN090528	MT121728	MT436130
FJ443690	KJ948658	MH705151	MN090529	MT121729	MT436131
FJ443713	KJ948659	MH705152	MN090530	MT121730	MT436132
FJ443734	KJ948660	MH705153	MN090531	MT121731	MT436133

FJ443757	KJ948661	MH705155	MN090532	MT121732	MT436134
FJ443782	KJ948662	MH705156	MN090533	MT121733	MT436135
FJ443839	KJ952242	MH705157	MN090534	MT121734	MT436136
FJ443861	KJ952283	MH705158	MN090535	MT121735	MT436137
FJ443883	KJ952314	MH705159	MN090536	MT121736	MT436138
FJ443906	KJ952346	MH705161	MN090537	MT121737	MT436139
FJ443938	KJ952376	MH705162	MN090538	MT121738	MT436140
FJ443963	KJ952432	MH705163	MN090539	MT121739	MT436141
FJ443989	KJ952505	MH714324	MN090540	MT121740	MT436142
FJ444007	KJ952535	MH714326	MN090541	MT121741	MT436143
FJ444035	KJ952562	MH714328	MN090542	MT121742	MT436144
FJ444058	KJ952667	MH714335	MN090543	MT121743	MT436145
FJ444077	KJ952689	MH714343	MN090544	MT121744	MT436146
FJ444092	KJ952712	MH714351	MN090545	MT121745	MT436147
FJ444120	KJ952732	MH715978	MN090546	MT121761	MT436148
FJ444159	KJ952765	MH742757	MN090547	MT121762	MT436149
FJ444186	KJ952820	MH742762	MN090548	MT121763	MT436150
FJ444208	KJ952852	MH742763	MN090549	MT121764	MT436151
FJ444230	KJ952897	MH742764	MN090550	MT121765	MT436152
FJ444253	KJ952917	MH742765	MN090551	MT121766	MT436153
FJ444281	KJ952968	MH742766	MN090552	MT121767	MT436154
FJ444305	KJ952991	MH742768	MN090553	MT121768	MT436155
FJ444325	KJ952992	MH742769	MN090554	MT121769	MT436156
FJ444347	KJ953058	MH742770	MN090555	MT121770	MT436157
FJ444369	KJ953089	MH746230	MN090556	MT121771	MT436158
FJ444392	KJ953119	MH746231	MN090557	MT121772	MT436159
FJ444416	KJ953149	MH746232	MN090558	MT121773	MT436160
FJ444436	KJ953175	MH746233	MN090559	MT121774	MT436161
FJ444457	KJ953197	MH746234	MN090560	MT121775	MT436162
FJ444480	KJ953229	MH746235	MN090561	MT121776	MT436163
FJ444501	KJ953282	MH746236	MN090562	MT121777	MT436164
FJ444523	KJ953316	MH746237	MN090563	MT121778	MT436165
FJ444543	KJ953335	MH746238	MN090564	MT121779	MT436166
FJ444566	KJ953366	MH746239	MN090565	MT121780	MT436167
FJ444589	KJ953408	MH746240	MN090566	MT121781	MT436168
FJ444611	KJ953438	MH746241	MN090567	MT121782	MT436169
FJ460499	KJ953459	MH746242	MN090568	MT121783	MT436170
FJ460500	KJ953503	MH746243	MN090569	MT121784	MT436171
FJ460501	KJ953525	MH746244	MN090570	MT121785	MT436172
FJ469682	KJ953555	MH746245	MN090571	MT121786	MT436173
FJ469683	KJ953596	MH746246	MN090572	MT121787	MT436174
FJ469684	KJ953611	MH746247	MN090573	MT121788	MT436175
FJ469685	KJ953634	MH746248	MN090574	MT121789	MT436176
FJ469686	KJ953664	MH746249	MN090575	MT121790	MT436177
FJ469687	KJ953685	MH746250	MN090576	MT121806	MT436178
FJ469688	KM081846	MH746251	MN090577	MT121807	MT436179
FJ469689	KM081850	MH746252	MN090578	MT121808	MT436180

FJ469690	KM081860	MH746253	MN090579	MT121810	MT436181
FJ469691	KM081862	MH746254	MN090580	MT121811	MT436182
FJ469692	KM081898	MH746255	MN090581	MT121812	MT436183
FJ469693	KM081934	MH746256	MN090582	MT121813	MT436184
FJ469694	KM081999	MH746257	MN090583	MT121814	MT436185
FJ469695	KM082062	MH746258	MN090584	MT121815	MT436186
FJ469696	KM082076	MH746259	MN090585	MT121816	MT436187
FJ469697	KM082124	MH746260	MN090586	MT121817	MT436188
FJ469698	KM082156	MH746261	MN090587	MT121818	MT436189
FJ469699	KM111555	MH746262	MN090588	MT121819	MT436190
FJ469700	KM217617	MH746263	MN090589	MT121820	MT436191
FJ469701	KM217780	MH746264	MN090590	MT121821	MT436192
FJ469702	KM217857	MH746265	MN090591	MT121822	MT436193
FJ469703	KM218138	MH746266	MN090592	MT121823	MT436194
FJ469704	KM218157	MH801989	MN090593	MT121824	MT436195
FJ469705	KM218159	MH843712	MN090594	MT121825	MT436196
FJ469706	KM218184	MH843713	MN090595	MT121826	MT436197
FJ469707	KM218210	MH843742	MN090596	MT121827	MT436198
FJ469708	KM218302	MH843743	MN090597	MT121828	MT436199
FJ469709	KM248765	MH843746	MN090598	MT121829	MT436200
FJ469710	KM258899	MH843747	MN090599	MT121830	MT436201
FJ469711	KM258919	MH843753	MN090600	MT121831	MT436202
FJ469712	KM258941	MH843754	MN090601	MT121832	MT436203
FJ469713	KM258969	MH843759	MN090602	MT121833	MT436204
FJ469714	KM258999	MH843761	MN090603	MT121834	MT436205
FJ469715	KM259030	MH843762	MN090604	MT121835	MT436206
FJ469716	KM259048	MH843763	MN090605	MT121836	MT436207
FJ469717	KM259061	MH843764	MN090606	MT121837	MT436208
FJ469718	KM259080	MH843765	MN090607	MT121838	MT436209
FJ469719	KM259085	MH843766	MN090608	MT121839	MT436210
FJ469720	KM259100	MH843767	MN090609	MT121840	MT436211
FJ469721	KM259127	MH843768	MN090610	MT121841	MT457783
FJ469722	KM259155	MH843769	MN090611	MT121842	MT457784
FJ469723	KM259197	MH843770	MN090612	MT121843	MT457785
FJ469724	KM259229	MH843771	MN090613	MT121859	MT457786
FJ469725	KM353586	MH843772	MN090614	MT121860	MT457787
FJ469726	KM353665	MH843773	MN090615	MT121861	MT457788
FJ469727	KM353701	MH843774	MN090616	MT121862	MT457789
FJ469728	KM353733	MH843775	MN090617	MT121863	MT457790
FJ469729	KM353807	MH843776	MN090618	MT121864	MT457791
FJ469730	KM353846	MH843778	MN090619	MT121865	MT457792
FJ469731	KM353922	MH843779	MN090620	MT121866	MT457793
FJ469732	KM354092	MH843780	MN090621	MT121867	MT457794
FJ469733	KM354114	MH843781	MN090622	MT121868	MT457795
FJ469734	KM354155	MH843782	MN090623	MT121879	MT457796
FJ469735	KM354232	MH843783	MN090624	MT121880	MT457797
FJ469737	KM354355	MH843784	MN090625	MT121881	MT457798

FJ469738	KM354431	MH843785	MN090626	MT121882	MT457799
FJ469739	KM354470	MH843786	MN090627	MT121883	MT457800
FJ469740	KM354585	MH843787	MN090628	MT121884	MT457801
FJ469741	KM354670	MH843788	MN090629	MT121885	MT457802
FJ469742	KM354703	MH843791	MN090630	MT121886	MT457803
FJ469743	KM354740	MH843792	MN090631	MT121887	MT457804
FJ469744	KM354781	MH843793	MN090632	MT121888	MT457805
FJ469745	KM354821	MH843818	MN090633	MT121889	MT457806
FJ469747	KM354862	MH843819	MN090634	MT121890	MT457807
FJ469748	KM354904	MH843820	MN090635	MT121891	MT457808
FJ469749	KM354962	MH843851	MN090636	MT121892	MT457809
FJ469750	KM355003	MH843857	MN090637	MT121893	MT457810
FJ469751	KM355044	MH843860	MN090638	MT121894	MT457811
FJ469752	KM355085	MH843872	MN090639	MT121895	MT457812
FJ469753	KM355159	MH843879	MN090640	MT121896	MT457813
FJ469754	KM438031	MH843883	MN090641	MT121897	MT457814
FJ469755	KM438032	MH843884	MN090642	MT121898	MT458931
FJ469756	KM516886	MH843885	MN090643	MT121899	MT458932
FJ469757	KM606628	MH843886	MN090644	MT121900	MT458933
FJ469758	KM606629	MH843887	MN090645	MT121901	MT458934
FJ469759	KM606631	MH843888	MN090646	MT121902	MT458935
FJ469760	KM606632	MH843890	MN090647	MT121903	MT559044
FJ469761	KM606633	MH843891	MN090648	MT121919	MT559045
FJ469762	KM606635	MH843892	MN090649	MT121920	MT559046
FJ469763	KM606636	MH843893	MN090650	MT121921	MT559047
FJ469764	KM606637	MH843894	MN090651	MT121922	MT559048
FJ469765	KM878816	MH843895	MN090652	MT121923	MT559049
FJ469766	KM974719	MH843896	MN090653	MT121924	MT559050
FJ469767	KM974720	MH843897	MN090654	MT121925	MT559051
FJ469768	KM986883	MH843898	MN090655	MT121926	MT559052
FJ469769	KM986886	MH843899	MN090656	MT121927	MT559053
FJ469770	KP109490	MH843900	MN090657	MT121928	MT559054
FJ469771	KP109491	MH843901	MN090658	MT121929	MT559055
FJ469772	KP109492	MH843902	MN090659	MT121930	MT559056
FJ496072	KP109493	MH843904	MN090660	MT121931	MT559057
FJ496081	KP109494	MH843905	MN090661	MT121932	MT559058
FJ515874	KP109495	MH843906	MN090662	MT121933	MT559059
FJ623475	KP109496	MH843907	MN090663	MT121934	MT559060
FJ623476	KP109497	MH843908	MN090664	MT121935	MT559061
FJ623477	KP109498	MH843909	MN090665	MT121936	MT559062
FJ623478	KP109499	MH843910	MN090666	MT121937	MT559063
FJ623479	KP109500	MH843911	MN090667	MT121938	MT559064
FJ623480	KP109501	MH843912	MN090668	MT121939	MT559065
FJ623481	KP109502	MH843913	MN090669	MT121940	MT559066
FJ623482	KP109503	MH843914	MN090670	MT121941	MT582420
FJ623483	KP109504	MH843915	MN090671	MT121942	MT582421
FJ623484	KP109505	MH843916	MN090672	MT121943	MT582422

FJ623485	KP109506	MH897911	MN090673	MT154920	MT582423
FJ623486	KP109507	MH897913	MN090674	MT154950	MT582424
FJ623487	KP109508	MH897915	MN090675	MT154963	MT611502
FJ623488	KP109510	MH897916	MN090676	MT154972	MT611503
FJ623489	KP109511	MH897917	MN090677	MT154974	MT611504
FJ623490	KP109512	MH897918	MN090678	MT154975	MT611505
FJ623491	KP109513	MH897920	MN090679	MT154978	MT611506
FJ623492	KP109514	MH899152	MN090680	MT154981	MT611507
FJ623493	KP109515	MH899153	MN090681	MT155001	MT650723
FJ623494	KP109516	MH899154	MN090682	MT155002	MT679550
FJ623495	KP109517	MH899155	MN090683	MT155004	MT679551
FJ647145	KP109518	MH899156	MN090684	MT155005	MT679552
FJ647147	KP109520	MH899157	MN090685	MT155006	MT679553
FJ647148	KP109521	MH899158	MN090686	MT155007	MT712270
FJ653098	KP109522	MH899160	MN090687	MT155008	MT712388
FJ653122	KP109523	MH899161	MN090688	MT155012	MT712389
FJ653146	KP109524	MH909568	MN090689	MT155015	MT712390
FJ653194	KP109525	MH909569	MN090690	MT155020	MT712391
FJ653218	KP109526	MH909570	MN090691	MT155021	MT783399
FJ653242	KP109527	MH933704	MN090692	MT155022	MT853116
FJ653278	KP170487	MH933705	MN090693	MT155027	MT853117
FJ653332	KP174771	MH933706	MN090694	MT155030	MT853118
FJ653380	KP178420	MH933707	MN090695	MT155031	MT853119
FJ653404	KP223776	MH933708	MN090696	MT155032	MT853120
FJ653428	KP223844	MH933709	MN090697	MT155033	MT853121
FJ653452	KP411822	MH933710	MN090698	MT155037	MT853122
FJ653476	KP411823	MH933711	MN090699	MT155043	MT853123
FJ653500	KP411824	MH933712	MN090700	MT155052	MT853124
FJ653524	KP411825	MH933713	MN090701	MT155055	MT853125
FJ653548	KP411826	MH933714	MN090702	MT155062	MT853126
FJ653572	KP411827	MH938677	MN090703	MT155063	MT853127
FJ653596	KP411828	MH938678	MN090704	MT155066	MT853128
FJ670515	KP411829	MH986013	MN090705	MT155080	MT853129
FJ670516	KP411830	MH986014	MN090706	MT155101	MT853130
FJ670517	KP411831	MH986016	MN090707	MT155132	MT853131
FJ670518	KP411832	MH986017	MN090708	MT155138	MT853132
FJ670519	KP411833	MK041550	MN090709	MT155153	MT853133
FJ670520	KP411834	MK041551	MN090710	MT155174	MT853134
FJ670521	KP411835	MK041553	MN090711	MT155175	MT853135
FJ670522	KP411836	MK041554	MN090712	MT155178	MT853136
FJ670523	KP411837	MK041556	MN090713	MT155179	MT853137
FJ670524	KP411838	MK041558	MN090714	MT155189	MT853138
FJ670525	KP411839	MK041560	MN090715	MT155191	MT853139
FJ670526	KP411840	MK041561	MN090716	MT155196	MT853140
FJ670527	KP411841	MK041562	MN090717	MT155273	MT853141
FJ670528	KP411842	MK041563	MN090718	MT155278	MT853142
FJ670529	KP411843	MK041564	MN090719	MT155299	MT853143

FJ670530	KP411844	MK041565	MN090720	MT155308	MT853144
FJ670531	KP411845	MK041566	MN090721	MT155310	MT853145
FJ687532	KP418805	MK041568	MN090722	MT155320	MT853146
FJ694790	KP418806	MK041570	MN090723	MT155323	MT853147
FJ694791	KP455640	MK041572	MN090724	MT155324	MT853148
FJ694792	KP668994	MK041573	MN090725	MT155325	MT853149
FJ711703	KP718914	MK041574	MN090726	MT155326	MT853150
FJ771006	KP718915	MK041575	MN090727	MT155327	MT853151
FJ771008	KP718916	MK041576	MN090728	MT155328	MT853152
FJ771009	KP718917	MK041577	MN090729	MT155329	MT853153
FJ771010	KP718918	MK041578	MN090730	MT155330	MT853154
FJ798320	KP718919	MK041580	MN090731	MT155331	MT853155
FJ798391	KP718920	MK041584	MN090732	MT155332	MT853156
FJ798415	KP718921	MK041585	MN090733	MT155333	MT853157
FJ817365	KP718922	MK041586	MN090734	MT155334	MT853158
FJ817366	KP718923	MK053935	MN090735	MT155335	MT853159
FJ817368	KP718924	MK076584	MN090736	MT155336	MT853160
FJ817370	KP718925	MK076663	MN090737	MT155337	MT853161
FJ817371	KP718926	MK086109	MN090738	MT155338	MT853162
FJ817372	KP718927	MK086110	MN090739	MT155339	MT853163
FJ846628	KP718928	MK086111	MN090740	MT155340	MT853164
FJ846633	KP718929	MK086112	MN090742	MT155341	MT853165
FJ853620	KP718930	MK086113	MN090743	MT155342	MT853166
FJ853621	KP718931	MK086114	MN090744	MT155343	MT853167
FJ853622	KP718932	MK086115	MN090745	MT155344	MT853168
FJ864679	KP718933	MK086116	MN090746	MT155345	MT853169
FJ866113	KP718934	MK086117	MN090747	MT155346	MT853170
FJ866117	KP718935	MK086118	MN090748	MT155347	MT853171
FJ866118	KP718936	MK086119	MN090749	MT155348	MT853172
FJ866119	KP718937	MK086120	MN090750	MT155349	MT853173
FJ866121	KP718938	MK086121	MN090751	MT155350	MT853174
FJ866124	KP754463	MK086122	MN090752	MT155351	MT853175
FJ866128	KP754464	MK086123	MN090753	MT155352	MT853176
FJ866131	KP754465	MK086124	MN090754	MT155353	MT853177
FJ866133	KP754466	MK086125	MN090755	MT155354	MT853178
FJ866136	KP754467	MK086126	MN090756	MT155355	MT853179
FJ866138	KP754468	MK086127	MN090757	MT155356	MT853180
FJ866139	KP754469	MK086128	MN090758	MT155357	MT853181
FJ900266	KP754470	MK086129	MN090759	MT155358	MT861212
FJ900267	KP754471	MK086130	MN090760	MT155359	MT861213
FJ900268	KP754472	MK086131	MN090761	MT155360	MT861214
FJ904244	KP754473	MK086132	MN090762	MT155361	MT861215
FJ952154	KP873161	MK095228	MN090763	MT155362	MT861216
FJ977091	KP873163	MK095229	MN090764	MT155363	MT861217
FJ977092	KR017771	MK095230	MN090765	MT155364	MT861218
FJ977095	KR017772	MK095236	MN090766	MT155365	MT861219
FM165626	KR017773	MK114636	MN090767	MT155366	MT861220

FM165645	KR017774	MK114800	MN090768	MT155367	MT861221
FM877777	KR017776	MK114990	MN090769	MT155368	MT861222
FM877779	KR017777	MK115138	MN090770	MT155369	MT861223
FM877780	KR017778	MK115343	MN090771	MT155370	MT861224
FM877781	KR017779	MK115381	MN090772	MT155371	MT861225
FM877782	KR019770	MK115482	MN090774	MT155372	MT861226
FN392873	KR019771	MK115595	MN090775	MT155373	MT861227
FN392874	KR019772	MK115694	MN090776	MT155374	MT861228
FN392875	KR051418	MK115871	MN090777	MT155375	MT861229
FN392876	KR051426	MK115946	MN090778	MT155376	MT861230
FN392877	KR051432	MK116093	MN090779	MT155377	MT861231
FN432725	KR051438	MK116905	MN090780	MT155378	MT861232
FR846408	KR051442	MK145772	MN090781	MT155379	MT861233
FR846409	KR051448	MK145773	MN090782	MT155380	MT861234
FR846410	KR051457	MK145774	MN090783	MT155381	MT861235
GQ153936	KR067667	MK145775	MN090784	MT155382	MT861236
GQ175881	KR067668	MK145776	MN090786	MT155383	MT861237
GQ175882	KR067669	MK145777	MN090788	MT155384	MT861238
GQ175883	KR067670	MK145778	MN090789	MT155385	MT861239
GQ222685	KR067671	MK145779	MN090790	MT189316	MT861240
GQ222686	KR182173	MK145780	MN090791	MT189317	MT861241
GQ229529	KR182174	MK145781	MN090792	MT189318	MT861242
GQ256645	KR182175	MK145782	MN090794	MT189319	MT861243
GQ256646	KR182176	MK145783	MN090795	MT189320	MT861244
GQ290462	KR182177	MK145784	MN090796	MT189321	MT861245
GQ304518	KR182180	MK145785	MN090797	MT189322	MT861246
GQ324613	KR182183	MK145786	MN090798	MT189325	MT861247
GQ324958	KR182184	MK145787	MN090799	MT189326	MT861248
GQ324959	KR182185	MK145788	MN090802	MT189327	MT861249
GQ324962	KR182187	MK145789	MN090803	MT189331	MT861250
GQ328744	KR182188	MK145790	MN090804	MT189333	MT861251
GQ351296	KR182189	MK145791	MN090805	MT189334	MT861252
GQ365649	KR182190	MK145792	MN090806	MT189337	MT861253
GQ365650	KR182191	MK145793	MN090807	MT189339	MT861254
GQ365651	KR182192	MK145794	MN090808	MT189346	MT861255
GQ365652	KR182196	MK145795	MN090809	MT189821	MT861256
GQ372986	KR182197	MK145796	MN090811	MT189857	MT861257
GQ372987	KR182198	MK147530	MN090812	MT189858	MT861258
GQ372988	KR182199	MK147531	MN090813	MT189859	MT861259
GQ372989	KR182200	MK147532	MN090814	MT189860	MT861260
GQ372990	KR182201	MK147533	MN090815	MT189861	MT861261
GQ477441	KR182202	MK147534	MN090816	MT189862	MT861262
GQ477442	KR182203	MK147535	MN090817	MT189863	MT861263
GQ477443	KR182204	MK147536	MN090818	MT189864	MT861264
GQ477444	KR182205	MK147537	MN090819	MT189865	MT861265
GQ477445	KR182206	MK147538	MN090820	MT189866	MT861266
GQ477446	KR182207	MK147539	MN090821	MT189867	MT861267

GQ477447	KR182208	MK147540	MN090822	MT189868	MT861268
GQ477448	KR182209	MK147541	MN090823	MT189869	MT861269
GQ477449	KR182210	MK147542	MN090825	MT189877	MT861270
GQ477450	KR182215	MK147543	MN090826	MT189880	MT861271
GQ477451	KR182217	MK147544	MN090829	MT189883	MT861272
GQ485312	KR182218	MK147545	MN090831	MT189927	MT861273
GQ485415	KR182219	MK147546	MN090832	MT189928	MT861274
GQ845124	KR182220	MK147547	MN090833	MT189929	MT861275
GQ845125	KR182221	MK147548	MN090834	MT189930	MT861276
GQ845126	KR182226	MK147549	MN090835	MT189932	MT861277
GQ862780	KR182228	MK147550	MN090836	MT189933	MT861278
GQ862781	KR182229	MK147551	MN090837	MT189934	MT861279
GQ916569	KR182230	MK147552	MN090838	MT189935	MT861280
GQ916570	KR182232	MK147553	MN090839	MT189936	MT861281
GQ916571	KR182234	MK147554	MN090841	MT189937	MT861282
GQ916572	KR182235	MK147555	MN090842	MT189938	MT861283
GQ916573	KR182236	MK147556	MN090843	MT189939	MT861284
GQ916574	KR182237	MK148475	MN090844	MT189940	MT861285
GQ916575	KR182238	MK148476	MN090845	MT189941	MT861286
GQ916576	KR182239	MK148477	MN090846	MT189942	MT861287
GQ916577	KR182240	MK148478	MN090847	MT189943	MT861288
GQ916578	KR182242	MK148479	MN090848	MT189944	MT861289
GQ916579	KR182243	MK148480	MN090849	MT189945	MT861290
GQ916580	KR182244	MK148481	MN090850	MT189946	MT861291
GQ916581	KR182245	MK148482	MN090851	MT189947	MT861292
GQ916582	KR182246	MK148483	MN090852	MT189948	MT861293
GQ916583	KR182247	MK148484	MN090854	MT189949	MT861294
GQ916584	KR182248	MK148485	MN090855	MT189950	MT861295
GQ916585	KR182249	MK148486	MN090856	MT189951	MT861297
GQ916587	KR182250	MK148487	MN090857	MT189952	MT861298
GQ916588	KR182251	MK148488	MN090858	MT189953	MT861299
GQ916589	KR182252	MK148489	MN090859	MT189954	MT861300
GQ916590	KR182253	MK148490	MN090860	MT189955	MT861301
GQ916591	KR182254	MK148491	MN090861	MT189956	MT861302
GQ916592	KR182255	MK148492	MN090862	MT189957	MT861303
GQ916593	KR182256	MK148493	MN090863	MT189958	MT861304
GQ999973	KR182257	MK148494	MN090865	MT189959	MT861305
GQ999975	KR182258	MK148495	MN090866	MT189960	MT861306
GQ999977	KR182259	MK148496	MN090867	MT189961	MT861307
GQ999980	KR182260	MK148497	MN090868	MT189962	MT861308
GQ999984	KR182261	MK148498	MN090869	MT189963	MT861309
GQ999985	KR182262	MK148499	MN090870	MT189964	MT861310
GQ999987	KR182263	MK148500	MN090871	MT189965	MT861311
GQ999988	KR182264	MK148501	MN090872	MT189966	MT861312
GQ999989	KR182265	MK148502	MN090873	MT189968	MT861313
GQ999990	KR182266	MK148503	MN090874	MT189969	MT861314
GU074012	KR182267	MK148504	MN090875	MT189970	MT861315

GU080161	KR182268	MK148505	MN090876	MT189972	MT861316
GU080162	KR182269	MK148506	MN090877	MT189973	MT861317
GU080164	KR182271	MK148507	MN090880	MT189974	MT861318
GU080165	KR182272	MK148508	MN090881	MT189975	MT861319
GU080166	KR182273	MK148509	MN090882	MT189976	MT861320
GU080168	KR182274	MK148510	MN090883	MT189977	MT861321
GU080171	KR182275	MK148511	MN090885	MT189978	MT861322
GU080173	KR182276	MK148512	MN090886	MT189979	MT861323
GU080174	KR182278	MK148513	MN090887	MT189980	MT861324
GU080176	KR182279	MK148514	MN090888	MT189981	MT861325
GU080178	KR182280	MK148515	MN090889	MT189982	MT861326
GU080179	KR182281	MK148516	MN090890	MT189983	MT861327
GU080180	KR182282	MK148517	MN090892	MT189984	MT861328
GU080181	KR182283	MK148518	MN090893	MT189985	MT861329
GU080182	KR182284	MK148519	MN090894	MT189986	MT861330
GU080183	KR182285	MK148520	MN090895	MT189987	MT861331
GU080184	KR182286	MK148521	MN090896	MT189988	MT861332
GU080185	KR182287	MK148522	MN090898	MT189989	MT861333
GU080186	KR182288	MK148523	MN090899	MT190008	MT861334
GU080187	KR182289	MK148524	MN090900	MT190009	MT861335
GU080190	KR182290	MK148525	MN090901	MT190010	MT861336
GU080191	KR182291	MK148526	MN090902	MT190011	MT861337
GU080192	KR182292	MK148527	MN090904	MT190012	MT861338
GU080193	KR182293	MK148528	MN090906	MT190013	MT861339
GU080194	KR182294	MK148529	MN090907	MT190014	MT861340
GU080195	KR182295	MK148530	MN090909	MT190015	MT861341
GU080197	KR182296	MK148531	MN090910	MT190016	MT861342
GU080198	KR182297	MK148532	MN090912	MT190017	MT861343
GU080199	KR182298	MK148533	MN090913	MT190018	MT861344
GU177863	KR182299	MK148534	MN090914	MT190019	MT861345
GU191360	KR182300	MK148535	MN090915	MT190020	MT861346
GU191372	KR182301	MK148536	MN090916	MT190021	MT861347
GU191384	KR182302	MK148537	MN090917	MT190022	MT861348
GU201494	KR182303	MK148538	MN090918	MT190023	MT861349
GU201495	KR182304	MK148539	MN090919	MT190024	MT861350
GU201497	KR182305	MK148540	MN090920	MT190025	MT861351
GU201498	KR182306	MK148541	MN090921	MT190026	MT861352
GU201499	KR182307	MK148542	MN090922	MT190027	MT861353
GU201500	KR182308	MK148543	MN090923	MT190034	MT861354
GU201504	KR182309	MK148544	MN090924	MT190035	MT861355
GU201508	KR182310	MK148545	MN090925	MT190040	MT861356
GU201512	KR182311	MK148546	MN090927	MT190041	MT861357
GU201513	KR182312	MK148547	MN090929	MT190249	MT861358
GU201514	KR182313	MK148548	MN090931	MT190250	MT861359
GU201516	KR182314	MK148549	MN090932	MT190251	MT861360
GU201611	KR182315	MK148550	MN090933	MT190255	MT861361
GU204919	KR182316	MK148551	MN090934	MT190259	MT861362

GU204922	KR182317	MK148552	MN090935	MT190260	MT861363
GU204925	KR182318	MK148553	MN090936	MT190264	MT861364
GU204929	KR182319	MK148554	MN090937	MT190269	MT861365
GU204933	KR182320	MK148555	MN090938	MT190272	MT861366
GU204937	KR182321	MK148556	MN090940	MT190275	MT861367
GU204941	KR182322	MK148560	MN090941	MT190281	MT861368
GU207884	KR182323	MK148561	MN090942	MT190292	MT861369
GU216724	KR182324	MK148562	MN097551	MT190297	MT861370
GU216803	KR182325	MK148563	MN097552	MT190298	MT861371
GU216838	KR182326	MK148564	MN097553	MT190309	MT861372
GU230137	KR182327	MK148565	MN097554	MT190318	MT861373
GU237072	KR182328	MK148566	MN097555	MT190319	MT861374
GU329048	KR182329	MK148567	MN097556	MT190320	MT861375
GU329053	KR182330	MK148568	MN097557	MT190321	MT861376
GU329063	KR182331	MK148569	MN097558	MT190323	MT861377
GU329078	KR182332	MK148570	MN097559	MT190324	MT861378
GU329094	KR182333	MK148571	MN097560	MT190325	MT861379
GU329107	KR182334	MK148572	MN097561	MT190326	MT861380
GU329121	KR182335	MK148574	MN097562	MT190327	MT861381
GU329131	KR182336	MK148577	MN097563	MT190328	MT861382
GU329144	KR182337	MK148581	MN097564	MT190331	MT861383
GU329154	KR182338	MK148582	MN097565	MT190332	MT861384
GU329164	KR182339	MK148583	MN097566	MT190333	MT861385
GU329175	KR182340	MK148585	MN097567	MT190338	MT861386
GU329184	KR182341	MK148586	MN097568	MT190339	MT861387
GU329195	KR182342	MK148589	MN097569	MT190342	MT861388
GU329205	KR182343	MK148590	MN097570	MT190343	MT861389
GU329216	KR182344	MK148595	MN097571	MT190345	MT861390
GU329236	KR182345	MK148619	MN097572	MT190347	MT861391
GU329257	KR182346	MK148620	MN097573	MT190349	MT861392
GU329271	KR182347	MK148621	MN097574	MT190350	MT861393
GU329289	KR182348	MK148622	MN097575	MT190353	MT861394
GU329300	KR182349	MK148623	MN097576	MT190358	MT861395
GU329310	KR182350	MK148624	MN097577	MT190360	MT861396
GU329321	KR182351	MK148625	MN097578	MT190625	MT861397
GU329333	KR182352	MK148626	MN097579	MT190626	MT861398
GU329343	KR182353	MK148627	MN097580	MT190627	MT861399
GU329354	KR182354	MK148628	MN097581	MT190628	MT861400
GU329364	KR182355	MK148629	MN097582	MT190629	MT861401
GU329375	KR182356	MK148630	MN097583	MT190631	MT861402
GU329389	KR182357	MK148631	MN097584	MT190632	MT861403
GU329399	KR182358	MK148632	MN097585	MT190636	MT861404
GU329415	KR182359	MK148633	MN097586	MT190637	MT861405
GU329437	KR182360	MK148634	MN097587	MT190638	MT861406
GU329458	KR182361	MK148635	MN097588	MT190639	MT861407
GU329481	KR182362	MK148636	MN097589	MT190640	MT861408
GU329490	KR182363	MK148637	MN097590	MT190641	MT861409

GU329500	KR182364	MK148638	MN097591	MT190642	MT861410
GU329510	KR182365	MK148639	MN097592	MT190650	MT861411
GU330247	KR182366	MK148640	MN097593	MT190658	MT861412
GU330333	KR182367	MK148641	MN097594	MT190743	MT861413
GU330396	KR182368	MK148642	MN097595	MT190745	MT861414
GU330462	KR182369	MK148643	MN097596	MT190747	MT861415
GU330499	KR182370	MK148644	MN097597	MT190751	MT861416
GU330524	KR182371	MK148645	MN097598	MT190752	MT861417
GU330549	KR182372	MK148646	MN097599	MT190753	MT861418
GU330598	KR182373	MK148647	MN097600	MT190754	MT861419
GU330622	KR182374	MK148648	MN097601	MT190755	MT861420
GU330692	KR182375	MK148649	MN097602	MT190756	MT861421
GU330731	KR182376	MK148650	MN097603	MT190757	MT861422
GU330809	KR182377	MK148651	MN097604	MT190758	MT861423
GU330839	KR182378	MK148652	MN097605	MT190759	MT861424
GU330862	KR182379	MK148653	MN097606	MT190762	MT861425
GU330916	KR182380	MK148654	MN097607	MT190764	MT861426
GU330956	KR182381	MK148655	MN097608	MT190767	MT861427
GU330994	KR182382	MK148656	MN097609	MT190768	MT861428
GU331039	KR182383	MK148657	MN097610	MT190771	MT861429
GU331066	KR182384	MK148658	MN097611	MT190776	MT861430
GU331094	KR182385	MK148659	MN097612	MT190777	MT861431
GU331183	KR182386	MK148660	MN097613	MT190778	MT861432
GU331218	KR182387	MK148661	MN097614	MT190779	MT861433
GU331463	KR182389	MK148662	MN097615	MT190780	MT861434
GU331496	KR182390	MK148663	MN097616	MT190781	MT861435
GU331550	KR182391	MK148664	MN097617	MT190782	MT861436
GU331590	KR182392	MK148665	MN097618	MT190783	MT861437
GU331722	KR182393	MK148666	MN097619	MT190784	MT861438
GU331748	KR182394	MK148667	MN097620	MT190785	MT861439
GU332506	KR182395	MK148668	MN097621	MT190786	MT861440
GU332507	KR182396	MK148669	MN097622	MT190787	MT861441
GU332508	KR182397	MK148670	MN097623	MT190788	MT861442
GU332509	KR182398	MK148671	MN097624	MT190789	MT861443
GU332510	KR182399	MK148672	MN097625	MT190790	MT861444
GU332511	KR182400	MK148673	MN097626	MT190791	MT861445
GU332512	KR182401	MK148674	MN097627	MT190792	MT861446
GU332513	KR182402	MK148675	MN097628	MT190793	MT861447
GU362013	KR182403	MK148676	MN097629	MT190794	MT861448
GU362881	KR182404	MK148677	MN097630	MT190795	MT861449
GU362882	KR182405	MK148678	MN097631	MT190796	MT861450
GU362883	KR182406	MK148679	MN097632	MT190797	MT861451
GU362885	KR182407	MK148680	MN097633	MT190798	MT861452
GU362886	KR182409	MK148681	MN097634	MT190799	MT861453
GU367395	KR182410	MK148682	MN097635	MT190800	MT861454
GU367398	KR182411	MK148683	MN097636	MT190801	MT861455
GU367399	KR182412	MK148684	MN097637	MT190802	MT861456

GU367403	KR182413	MK148685	MN097638	MT190803	MT861457
GU367404	KR182414	MK148686	MN097639	MT190804	MT861458
GU367405	KR182415	MK148687	MN097640	MT190805	MT861459
GU367406	KR182416	MK148688	MN097641	MT190806	MT861460
GU367407	KR182417	MK148689	MN097642	MT190807	MT861461
GU367408	KR182418	MK148690	MN097643	MT190808	MT861462
GU367409	KR182419	MK148691	MN097644	MT190809	MT861463
GU367410	KR182420	MK148692	MN097645	MT190810	MT861464
GU367412	KR182421	MK148693	MN097646	MT190811	MT861465
GU455425	KR182422	MK148694	MN097647	MT190812	MT861466
GU455456	KR182423	MK148695	MN097648	MT190813	MT861467
GU455476	KR182424	MK148696	MN097649	MT190814	MT861468
GU455494	KR182425	MK148697	MN097650	MT190815	MT861469
GU455514	KR182426	MK148698	MN097651	MT190816	MT861470
GU475013	KR182427	MK148699	MN097652	MT190817	MT861471
GU475014	KR182428	MK148700	MN097653	MT190818	MT861472
GU475015	KR182429	MK158945	MN097654	MT190819	MT861473
GU475016	KR182430	MK158946	MN097655	MT190820	MT861474
GU475017	KR182431	MK164663	MN097656	MT190821	MT861475
GU475018	KR182432	MK164664	MN097657	MT190822	MT861477
GU475019	KR182433	MK164665	MN097658	MT190823	MT861478
GU475020	KR182434	MK164666	MN097659	MT190824	MT861479
GU475021	KR182435	MK164667	MN097660	MT190825	MT861480
GU475022	KR182436	MK164668	MN097661	MT190826	MT861481
GU475023	KR182437	MK164669	MN097662	MT190827	MT861482
GU475024	KR182438	MK164670	MN097663	MT190828	MT861483
GU475025	KR182439	MK164671	MN097664	MT190829	MT861484
GU475026	KR182440	MK164672	MN097665	MT190830	MT861485
GU475027	KR182441	MK164673	MN097666	MT190831	MT861486
GU475028	KR182442	MK164674	MN097667	MT190832	MT861487
GU475029	KR182443	MK164675	MN097668	MT190833	MT861488
GU475030	KR182444	MK164676	MN097669	MT190834	MT861489
GU475031	KR182445	MK164677	MN097670	MT190835	MT861490
GU475032	KR182446	MK164678	MN097671	MT190836	MT861491
GU475033	KR182447	MK164679	MN097672	MT190837	MT861492
GU475034	KR182448	MK164680	MN097673	MT190838	MT861493
GU475035	KR182449	MK164681	MN097674	MT190839	MT861494
GU475036	KR182450	MK164682	MN097675	MT190840	MT861496
GU475037	KR182451	MK164683	MN097676	MT190841	MT861497
GU475038	KR182452	MK169417	MN097677	MT190842	MT861498
GU475039	KR182453	MK169418	MN097678	MT190843	MT861499
GU475040	KR182454	MK169420	MN097679	MT190844	MT861500
GU475042	KR182455	MK169421	MN097680	MT190845	MT861501
GU475043	KR182456	MK169422	MN097681	MT190846	MT861502
GU475044	KR182457	MK169427	MN097682	MT190847	MT861503
GU475045	KR182458	MK169430	MN097683	MT190848	MT861504
GU475046	KR182459	MK169431	MN097684	MT190849	MT861505

GU481107	KR182460	MK169433	MN097685	MT190850	MT861506
GU481156	KR182461	MK169434	MN097686	MT190851	MT861507
GU481203	KR182462	MK169435	MN097687	MT190852	MT861508
GU481254	KR182463	MK169437	MN097688	MT190853	MT861509
GU481277	KR182464	MK169438	MN097689	MT190854	MT861510
GU481329	KR182465	MK169440	MN097690	MT190855	MT861511
GU481371	KR182466	MK169441	MN097691	MT190856	MT861512
GU481392	KR182467	MK169443	MN097692	MT190857	MT861513
GU481421	KR182468	MK169447	MN097693	MT190858	MT861514
GU481472	KR182469	MK169448	MN097694	MT190859	MT861515
GU481519	KR182470	MK169449	MN097695	MT190860	MT861516
GU481537	KR182471	MK169450	MN097696	MT190861	MT861517
GU481566	KR182472	MK169451	MN097697	MT190862	MT861518
GU481591	KR182473	MK169452	MN116200	MT190863	MT861519
GU481615	KR423015	MK169453	MN116201	MT190864	MT861520
GU481636	KR423028	MK169454	MN116203	MT190865	MT861521
GU481661	KR423052	MK169455	MN116204	MT190866	MT861522
GU562001	KR423118	MK169456	MN116205	MT190867	MT861523
GU562028	KR423153	MK169457	MN116206	MT190868	MT861524
GU562058	KR423183	MK169458	MN116207	MT190869	MT861525
GU562080	KR423212	MK169459	MN116208	MT190870	MT861526
GU562105	KR423234	MK169460	MN116209	MT190871	MT861527
GU562134	KR423279	MK169461	MN116210	MT190872	MT861528
GU562153	KR423303	MK169462	MN116211	MT190873	MT861529
GU562218	KR423319	MK169463	MN116212	MT190874	MT861530
GU562249	KR423340	MK169464	MN124512	MT190875	MT861531
GU562271	KR423358	MK169465	MN153475	MT190876	MT861532
GU564221	KR423363	MK169466	MN153476	MT190877	MT861533
GU564222	KR423373	MK169467	MN153477	MT190878	MT861534
GU564223	KR423391	MK169468	MN153478	MT190879	MT861535
GU564224	KR423410	MK169469	MN153479	MT190880	MT861536
GU564225	KR423423	MK169470	MN153480	MT190881	MT861537
GU564227	KR423435	MK169471	MN153481	MT190882	MT861538
GU564228	KR423444	MK169472	MN153482	MT190883	MT861539
GU564229	KR423464	MK169473	MN153483	MT190884	MT861540
GU564230	KR423482	MK169474	MN153484	MT190885	MT861541
GU595148	KR423500	MK169475	MN153485	MT190886	MT861542
GU595149	KR423527	MK169476	MN153486	MT190887	MT861544
GU595150	KR423545	MK169477	MN153487	MT190888	MT861545
GU595151	KR423553	MK169478	MN153488	MT190889	MT861546
GU595152	KR423567	MK169479	MN153489	MT190890	MT861547
GU595153	KR423590	MK169480	MN153490	MT190891	MT861548
GU595156	KR423602	MK169481	MN153491	MT190892	MT861549
GU595158	KR423619	MK169482	MN153492	MT190893	MT861550
GU595160	KR423637	MK169483	MN153493	MT190894	MT861551
GU727873	KR423648	MK169484	MN153494	MT190895	MT861552
GU727875	KR423693	MK169485	MN153495	MT190896	MT861553

GU727879	KR423725	MK169486	MN153496	MT190897	MT861554
GU727880	KR423796	MK169487	MN153497	MT190898	MT861555
GU727884	KR425429	MK169488	MN172222	MT190899	MT861556
GU727886	KR425448	MK169489	MN172223	MT190900	MT861557
GU727887	KR820294	MK169490	MN172224	MT190901	MT861558
GU727890	KR820314	MK169493	MN172225	MT190902	MT861559
GU727898	KR820324	MK169495	MN178644	MT190903	MT861560
GU727913	KR820326	MK169499	MN178645	MT190904	MT861561
GU727917	KR820341	MK169504	MN187301	MT190905	MT861562
GU727919	KR820358	MK169506	MN187302	MT190906	MT861563
GU727943	KR820367	MK169510	MN187303	MT190907	MT861564
GU727957	KR820385	MK169512	MN202471	MT190908	MT861565
GU727958	KR820394	MK169513	MN202472	MT190909	MT861566
GU727961	KR820415	MK169514	MN237642	MT190910	MT861567
GU727972	KR820422	MK169515	MN237643	MT190911	MT861568
GU727977	KR820440	MK169516	MN237644	MT190912	MT861569
GU727980	KR822830	MK169517	MN237645	MT190913	MT861570
GU727988	KR861257	MK169518	MN237646	MT190914	MT861571
GU728005	KR861259	MK169519	MN271384	MT190915	MT861572
GU728008	KR861264	MK169520	MN336526	MT190916	MT861573
GU728014	KR861269	MK169521	MN336527	MT190917	MT861574
GU728025	KR861270	MK169522	MN336528	MT190918	MT861575
GU728030	KR861271	MK169523	MN336529	MT190919	MT861576
GU728039	KR861273	MK169524	MN336530	MT190920	MT861577
GU728041	KR861276	MK169525	MN336531	MT190921	MT861578
GU728050	KR861279	MK169526	MN337382	MT190922	MT861579
GU728051	KR861280	MK169527	MN449474	MT190923	MT861580
GU728053	KR861281	MK169528	MN449475	MT190924	MT861581
GU728054	KR861282	MK169529	MN449476	MT190925	MT861582
GU728056	KR861283	MK169530	MN452901	MT190926	MT861583
GU728072	KR861302	MK169531	MN466965	MT190927	MT861584
GU728073	KR861304	MK169532	MN466967	MT190928	MT861585
GU728093	KR861308	MK169533	MN466972	MT190929	MT861586
GU728094	KR861311	MK169534	MN466977	MT190930	MT861587
GU728101	KR861312	MK169535	MN466979	MT190931	MT861588
GU728134	KR861313	MK169536	MN466980	MT190932	MT861589
GU728137	KR861314	MK169537	MN466982	MT190933	MT861590
GU728150	KR861315	MK169538	MN466984	MT190934	MT861591
GU728151	KR861316	MK169539	MN466985	MT190935	MT861592
GU728152	KR861317	MK169540	MN466986	MT190936	MT861593
GU728155	KR861318	MK169541	MN466989	MT190937	MT861594
GU728157	KR861319	MK169542	MN466990	MT190938	MT861595
GU728158	KR861320	MK169543	MN466991	MT190939	MT861596
GU728163	KR861321	MK169545	MN466992	MT190940	MT861597
GU728173	KR861322	MK169546	MN466995	MT190941	MT861598
GU728176	KR861323	MK169547	MN466996	MT190942	MT861599
GU728179	KR861324	MK169550	MN466997	MT190943	MT861600

GU728181	KR861325	MK169551	MN466998	MT190944	MT861601
GU728185	KR861326	MK169553	MN466999	MT190945	MT861602
GU728189	KR861327	MK169554	MN467000	MT190946	MT861603
GU728191	KR861328	MK169555	MN467001	MT190947	MT861604
GU728195	KR861329	MK169557	MN467002	MT190948	MT861605
GU728201	KR861330	MK169559	MN467003	MT190949	MT861606
GU728204	KR861331	MK169560	MN467004	MT190950	MT861607
GU728205	KR861332	MK169566	MN467005	MT190951	MT861608
GU728209	KR861333	MK169570	MN467006	MT190952	MT861609
GU728216	KR861334	MK169573	MN467007	MT190953	MT861610
GU728222	KR861335	MK169578	MN467010	MT190954	MT861611
GU728223	KR861336	MK169579	MN467011	MT190955	MT861612
GU728225	KR861337	MK169580	MN467014	MT190956	MT861613
GU728226	KR861338	MK169581	MN467015	MT190957	MT861614
GU728227	KR861339	MK169584	MN467022	MT190958	MT861615
GU728229	KR861340	MK169586	MN467026	MT190959	MT861616
GU728234	KR861341	MK169587	MN467027	MT190960	MT861617
GU728237	KR861342	MK169588	MN467034	MT190961	MT861618
GU728238	KR861343	MK169589	MN467035	MT190962	MT861619
GU728240	KR861344	MK169590	MN467050	MT190963	MT861620
GU728244	KR861345	MK169591	MN467104	MT190964	MT861621
GU728251	KR868834	MK169592	MN467209	MT190965	MT861622
GU728253	KR868847	MK169593	MN467237	MT190966	MT861623
GU728255	KR868869	MK169594	MN467250	MT190967	MT861624
GU728263	KR868894	MK169595	MN467271	MT190968	MT861625
GU728268	KR868913	MK169596	MN467301	MT190969	MT861626
GU728271	KR914675	MK169597	MN467309	MT190970	MT861627
GU728274	KR914676	MK169598	MN467313	MT190971	MT861628
GU728276	KR914677	MK169599	MN467314	MT190972	MT861629
GU728277	KR914678	MK169600	MN467315	MT190973	MT861630
GU728279	KT008649	MK169601	MN467316	MT190974	MT861631
GU728280	KT022360	MK169602	MN467317	MT190975	MT861632
GU728283	KT022361	MK169603	MN467318	MT190976	MT861633
GU728287	KT022362	MK169604	MN467319	MT190977	MT861634
GU728288	KT022363	MK169605	MN467320	MT190978	MT861635
GU728290	KT022364	MK169606	MN467323	MT190979	MT861636
GU728293	KT022365	MK169607	MN467327	MT190980	MT861637
GU728294	KT022366	MK169608	MN467328	MT190981	MT861638
GU728295	KT022367	MK169609	MN467329	MT190982	MT861639
GU728297	KT022368	MK169610	MN467330	MT190983	MT861640
GU728304	KT022369	MK169611	MN467334	MT190984	MT861641
GU728308	KT022370	MK169612	MN467336	MT190985	MT861642
GU728311	KT022371	MK169613	MN467337	MT190986	MT861643
GU728313	KT022372	MK169614	MN467338	MT190987	MT861644
GU728321	KT022373	MK169615	MN467339	MT190988	MT861645
GU728322	KT022374	MK169616	MN467340	MT190989	MT861646
GU728323	KT022375	MK169617	MN467355	MT190990	MT861647

GU728327	KT022376	MK169618	MN467383	MT190991	MT861648
GU728330	KT022377	MK169619	MN467384	MT190992	MT861649
GU728333	KT022378	MK169620	MN467385	MT190993	MT861650
GU728334	KT022379	MK169621	MN467386	MT190994	MT861651
GU728336	KT022380	MK169622	MN467387	MT190995	MT861652
GU728337	KT022381	MK169623	MN467388	MT190996	MT861653
GU728339	KT022382	MK169624	MN467389	MT190997	MT861654
GU728341	KT022383	MK169625	MN467390	MT190998	MT861655
GU728343	KT022384	MK169626	MN467391	MT190999	MT861656
GU728345	KT022385	MK169627	MN467392	MT191000	MT861657
GU728346	KT022386	MK169628	MN467393	MT191001	MT861658
GU728348	KT022387	MK169629	MN467394	MT191002	MT861659
GU728349	KT022388	MK169630	MN467395	MT191003	MT861660
GU728352	KT022389	MK169631	MN467396	MT191004	MT861661
GU728357	KT022390	MK169632	MN467397	MT191005	MT861662
GU728359	KT022391	MK169633	MN485971	MT191006	MT861663
GU728368	KT022392	MK169634	MN485972	MT191007	MT861664
GU728369	KT022393	MK169635	MN485973	MT191008	MT861665
GU728370	KT022394	MK169636	MN485974	MT191009	MT861666
GU728371	KT022395	MK169637	MN485975	MT191010	MT861667
GU728375	KT022396	MK169638	MN485977	MT191011	MT861668
GU728376	KT022397	MK169639	MN485978	MT191012	MT861669
GU728383	KT022398	MK169640	MN485979	MT191013	MT861670
GU728392	KT022399	MK169641	MN485980	MT191014	MT861671
GU728395	KT022400	MK169642	MN485981	MT191015	MT861672
GU728396	KT022401	MK169643	MN485982	MT191016	MT861673
GU728404	KT022402	MK169644	MN485983	MT191017	MT861674
GU728406	KT022403	MK169645	MN485984	MT191018	MT861675
GU728409	KT022404	MK169646	MN485985	MT191019	MT861676
GU728410	KT022405	MK169647	MN485986	MT191020	MT861677
GU728412	KT022406	MK169648	MN485987	MT191021	MT861678
GU728413	KT022407	MK169649	MN485988	MT191022	MT861679
GU728416	KT022408	MK169650	MN485989	MT191023	MT861680
GU733713	KT022409	MK169651	MN485990	MT191024	MT861681
GU939049	KT022410	MK169652	MN485991	MT191025	MT861682
GU939062	KT022411	MK169653	MN485992	MT191026	MT861683
GU939124	KT022412	MK169654	MN485993	MT191027	MT861684
GU939129	KT022413	MK169655	MN485994	MT191028	MT861685
GU939143	KT022414	MK169656	MN485995	MT191029	MT861686
HM026455	KT022415	MK169657	MN485996	MT191030	MT861687
HM026456	KT022416	MK169658	MN485997	MT191031	MT861688
HM026457	KT022417	MK169659	MN485998	MT191032	MT861689
HM026458	KT074935	MK169660	MN485999	MT191033	MT861690
HM026459	KT124747	MK169661	MN486000	MT191034	MT861691
HM026460	KT124748	MK169662	MN486001	MT191035	MT861692
HM027823	KT124749	MK169663	MN486002	MT191036	MT861693
HM027827	KT124750	MK169664	MN486003	MT191037	MT861694

HM027838	KT124751	MK169665	MN486004	MT191038	MT861695
HM027846	KT124752	MK169666	MN486005	MT191039	MT861696
HM027850	KT124753	MK169667	MN486006	MT191040	MT861697
HM027851	KT124754	MK169668	MN486007	MT191041	MT861698
HM027859	KT124755	MK169669	MN486008	MT191042	MT861699
HM027863	KT124756	MK169670	MN486009	MT191043	MT861700
HM027864	KT124757	MK169671	MN486010	MT191044	MT861701
HM027869	KT124758	MK169672	MN486011	MT191045	MT861702
HM030559	KT124759	MK169673	MN486012	MT191046	MT861703
HM030560	KT124760	MK169674	MN486013	MT191047	MT861704
HM030561	KT124761	MK169675	MN486014	MT191048	MT861705
HM030562	KT124762	MK169676	MN486015	MT191049	MT861706
HM030563	KT124763	MK169677	MN486016	MT191050	MT861707
HM030564	KT124764	MK169678	MN486017	MT191051	MT861708
HM030565	KT124765	MK169682	MN486018	MT191052	MT861709
HM036739	KT124766	MK169683	MN486019	MT191053	MT861710
HM036760	KT124767	MK169710	MN486020	MT191054	MT861711
HM036792	KT124768	MK169714	MN486021	MT191055	MT861712
HM036812	KT124769	MK169720	MN486022	MT191056	MT861713
HM036832	KT124770	MK169721	MN486023	MT191057	MT861714
HM036843	KT124771	MK169723	MN486024	MT191058	MT861715
HM036864	KT124772	MK169728	MN486025	MT191059	MT861716
HM036903	KT124773	MK169731	MN486026	MT191060	MT861717
HM036960	KT124774	MK169735	MN486027	MT191061	MT861718
HM036983	KT124775	MK169745	MN486028	MT191062	MT861719
HM037006	KT124776	MK169747	MN486029	MT191063	MT861720
HM067748	KT124777	MK169756	MN486030	MT191064	MT861721
HM067749	KT124778	MK169758	MN486031	MT191065	MT861722
HM068551	KT124779	MK169761	MN486032	MT191066	MT861723
HM068552	KT124782	MK169764	MN486033	MT191067	MT861724
HM068554	KT124783	MK169765	MN486034	MT191068	MT861725
HM068555	KT124784	MK169777	MN486035	MT191069	MT861726
HM068556	KT124785	MK169780	MN486036	MT191070	MT861727
HM068596	KT124786	MK169781	MN486037	MT191071	MT861728
HM068598	KT124787	MK169784	MN486038	MT191072	MT861729
HM070449	KT124788	MK169785	MN486039	MT191073	MT861730
HM070491	KT124790	MK169787	MN486040	MT191074	MT861731
HM070529	KT124791	MK169788	MN486041	MT191075	MT861732
HM070571	KT124792	MK169789	MN486042	MT191076	MT861733
HM070630	KT124794	MK169790	MN486043	MT191077	MT861734
HM070674	KT124795	MK169791	MN486044	MT191078	MT861735
HM070791	KT124796	MK169792	MN486045	MT191079	MT861736
HM100716	KT124797	MK169793	MN486046	MT191080	MT861737
HM138656	KT124798	MK169794	MN486047	MT191081	MT861738
HM204580	KT124799	MK169795	MN498126	MT191082	MT861739
HM204581	KT124801	MK169796	MN515491	MT191083	MT861740
HM204582	KT124802	MK169797	MN515571	MT191084	MT861743

HM204583	KT124803	MK169798	MN515777	MT191085	MT861745
HM204584	KT124804	MK169799	MN515912	MT191086	MT861748
HM204585	KT124805	MK169800	MN515943	MT191087	MT861750
HM204587	KT124806	MK169801	MN515955	MT191088	MT861751
HM204588	KT124807	MK169802	MN516274	MT191089	MT861753
HM204589	KT124808	MK169803	MN516275	MT191090	MT861755
HM204590	KT124809	MK169804	MN516276	MT191091	MT861757
HM204591	KT124810	MK169805	MN516277	MT191092	MT861759
HM204592	KT124811	MK169806	MN516278	MT191093	MT861760
HM204593	KT124812	MK169807	MN516279	MT191094	MT861763
HM204594	KT124813	MK169808	MN516280	MT191095	MT861765
HM204595	KT124814	MK169809	MN516281	MT191096	MT861767
HM204596	KT152839	MK169810	MN516282	MT191097	MT861770
HM204597	KT152840	MK169811	MN516283	MT191098	MT861773
HM204598	KT152841	MK169812	MN516284	MT191099	MT861775
HM204600	KT152842	MK169813	MN516285	MT191100	MT861777
HM204601	KT152843	MK169814	MN516286	MT191101	MT861780
HM204602	KT152846	MK169815	MN516287	MT191102	MT861782
HM204604	KT175202	MK169816	MN516288	MT191103	MT861784
HM204605	KT175204	MK169817	MN516289	MT191104	MT861786
HM204606	KT175205	MK169818	MN516290	MT191105	MT861787
HM204607	KT183056	MK169819	MN516291	MT191106	MT861788
HM204608	KT183068	MK169820	MN516292	MT191107	MT861789
HM204609	KT183073	MK169821	MN516293	MT191108	MT861790
HM204610	KT183078	MK169822	MN516294	MT191109	MT861791
HM204611	KT183084	MK169823	MN516295	MT191110	MT861792
HM204613	KT183114	MK169824	MN516296	MT191111	MT861793
HM204614	KT183135	MK169825	MN516297	MT191112	MT861794
HM204615	KT183150	MK169826	MN516298	MT191113	MT861795
HM204616	KT183168	MK169827	MN516299	MT191114	MT861796
HM204617	KT183250	MK169828	MN516300	MT191115	MT861797
HM204618	KT183252	MK169829	MN516301	MT191116	MT861798
HM204619	KT183271	MK169830	MN516302	MT191117	MT861799
HM204620	KT183301	MK169831	MN516303	MT191118	MT861800
HM204621	KT185678	MK169832	MN516304	MT191119	MT861801
HM204622	KT185724	MK169833	MN516305	MT191120	MT861802
HM204623	KT185744	MK169834	MN516306	MT191121	MT861803
HM204624	KT185793	MK169835	MN516307	MT191122	MT861804
HM204625	KT185851	MK169836	MN516308	MT191123	MT861805
HM204626	KT185921	MK169837	MN516309	MT191124	MT861806
HM204627	KT185941	MK169838	MN516310	MT191125	MT861807
HM204628	KT185962	MK169839	MN516312	MT191126	MT861808
HM204629	KT185983	MK169840	MN516313	MT191127	MT861809
HM204630	KT186005	MK169841	MN516314	MT191128	MT861810
HM204631	KT200348	MK169842	MN516315	MT191129	MT861811
HM204632	KT200349	MK169843	MN516316	MT191130	MT861812
HM204633	KT200350	MK169844	MN516317	MT191131	MT861813

HM204634	KT200351	MK169845	MN516318	MT191132	MT861814
HM204635	KT200352	MK169846	MN516319	MT191133	MT861815
HM204636	KT200353	MK169847	MN516320	MT191134	MT861816
HM204637	KT200354	MK169848	MN516321	MT191135	MT861817
HM204638	KT200355	MK169849	MN516322	MT191136	MT861818
HM204639	KT200356	MK169850	MN516323	MT191137	MT861819
HM204640	KT200357	MK169851	MN516324	MT191138	MT861820
HM204641	KT200358	MK169852	MN516325	MT191139	MT861821
HM204642	KT223503	MK169853	MN516326	MT191140	MT861822
HM204643	KT223759	MK169854	MN516328	MT191141	MT861823
HM204644	KT223760	MK169855	MN516329	MT191142	MT861824
HM204645	KT252544	MK169856	MN516331	MT191143	MT861825
HM204646	KT252545	MK169857	MN516332	MT191144	MT861826
HM204647	KT276254	MK169858	MN516333	MT191145	MT861827
HM204648	KT276255	MK169859	MN516334	MT191146	MT861828
HM215249	KT276256	MK169860	MN516335	MT191147	MT861829
HM215251	KT276257	MK169861	MN516336	MT191148	MT861830
HM215253	KT276258	MK169862	MN516337	MT191149	MT861831
HM215254	KT276259	MK169863	MN516338	MT191150	MT861832
HM215255	KT276260	MK169864	MN516339	MT191151	MT861833
HM215258	KT276261	MK169865	MN516340	MT191152	MT861834
HM215259	KT276262	MK169869	MN516341	MT191153	MT861835
HM215261	KT276263	MK169873	MN516342	MT191154	MT861836
HM215262	KT276264	MK169876	MN516343	MT191155	MT861837
HM215263	KT276265	MK169878	MN516344	MT191156	MT861838
HM215264	KT276266	MK169880	MN516345	MT191157	MT861839
HM215265	KT276267	MK169882	MN516346	MT191158	MT861840
HM215266	KT276268	MK169886	MN516347	MT191159	MT861841
HM215267	KT276269	MK169891	MN516348	MT191160	MT861842
HM215270	KT276270	MK169892	MN516349	MT191161	MT861843
HM215271	KT276271	MK169896	MN516350	MT191162	MT861844
HM215272	KT283686	MK169897	MN516351	MT191163	MT861845
HM215273	KT283704	MK169898	MN516352	MT191164	MT861846
HM215274	KT283729	MK169899	MN516353	MT191165	MT861847
HM215275	KT283740	MK169900	MN516354	MT191166	MT861848
HM215276	KT283762	MK169901	MN516355	MT191167	MT861849
HM215277	KT283800	MK169902	MN516356	MT191168	MT861850
HM215279	KT283856	MK169903	MN516357	MT191169	MT861851
HM215280	KT283923	MK169904	MN516358	MT191170	MT861852
HM215281	KT284371	MK169905	MN516359	MT191171	MT861853
HM215282	KT321211	MK169906	MN516360	MT191172	MT861854
HM215283	KT372798	MK169907	MN516361	MT191173	MT861855
HM215286	KT427649	MK169908	MN516362	MT191174	MT861856
HM215287	KT427650	MK169909	MN516363	MT191175	MT861857
HM215288	KT427651	MK169910	MN516364	MT191176	MT861858
HM215289	KT427652	MK169911	MN516365	MT191177	MT861859
HM215290	KT427654	MK169912	MN516366	MT191178	MT861860

HM215291	KT427656	MK169913	MN516367	MT191179	MT861861
HM215292	KT427660	MK169914	MN516368	MT191180	MT861862
HM215293	KT427667	MK169915	MN516369	MT191181	MT861863
HM215294	KT427668	MK169916	MN516370	MT191182	MT861864
HM215296	KT427669	MK169917	MN516371	MT191183	MT861865
HM215297	KT427670	MK169918	MN516372	MT191184	MT861866
HM215298	KT427671	MK169919	MN516373	MT191185	MT861867
HM215299	KT427673	MK169920	MN516374	MT191186	MT861868
HM215300	KT427674	MK169921	MN516375	MT191187	MT861869
HM215301	KT427675	MK169922	MN516376	MT191188	MT861870
HM215302	KT427676	MK169923	MN516377	MT191189	MT861871
HM215303	KT427677	MK169924	MN516378	MT191190	MT861872
HM215304	KT427678	MK169925	MN516379	MT191191	MT861873
HM215305	KT427679	MK169926	MN516380	MT191192	MT861874
HM215306	KT427680	MK169927	MN516381	MT191193	MT861875
HM215307	KT427681	MK169928	MN516382	MT191194	MT861876
HM215308	KT427682	MK169929	MN516383	MT191195	MT861877
HM215309	KT427683	MK169930	MN516384	MT191196	MT861878
HM215310	KT427684	MK169931	MN516385	MT191197	MT861879
HM215311	KT427685	MK169932	MN516386	MT191198	MT861880
HM215312	KT427686	MK169933	MN516387	MT191199	MT861881
HM215313	KT427687	MK169934	MN516388	MT191200	MT861882
HM215314	KT427688	MK169935	MN516389	MT191201	MT861884
HM215315	KT427689	MK169936	MN516390	MT191202	MT861885
HM215317	KT427690	MK169937	MN516391	MT191203	MT861886
HM215318	KT427691	MK169938	MN516392	MT191204	MT861887
HM215319	KT427692	MK169939	MN516394	MT191205	MT861888
HM215320	KT427693	MK169940	MN516395	MT191206	MT861889
HM215321	KT427694	MK169941	MN516396	MT191207	MT861890
HM215322	KT427695	MK169942	MN516397	MT191208	MT861891
HM215323	KT427696	MK169943	MN516398	MT191209	MT861892
HM215324	KT427697	MK169944	MN516399	MT191210	MT861893
HM215325	KT427698	MK169945	MN516400	MT191211	MT861894
HM215326	KT427699	MK169946	MN516401	MT191212	MT861895
HM215327	KT427700	MK169947	MN516402	MT191213	MT861896
HM215328	KT427701	MK169948	MN516403	MT191214	MT861897
HM215329	KT427702	MK169949	MN516404	MT191215	MT861898
HM215330	KT427703	MK169950	MN516405	MT191216	MT861899
HM215331	KT427704	MK169951	MN516406	MT191217	MT861900
HM215333	KT427705	MK169952	MN516407	MT191218	MT861901
HM215335	KT427706	MK169953	MN516408	MT191219	MT861902
HM215336	KT427707	MK169954	MN516409	MT191220	MT861903
HM215337	KT427708	MK169955	MN516410	MT191221	MT861904
HM215338	KT427709	MK169956	MN516411	MT191222	MT861905
HM215339	KT427710	MK169957	MN516412	MT191223	MT861906
HM215341	KT427711	MK169958	MN516413	MT191224	MT861907
HM215343	KT427712	MK169959	MN516414	MT191225	MT861908

HM215344	KT427713	MK169960	MN516415	MT194125	MT861909
HM215347	KT427714	MK169961	MN516416	MT194126	MT861910
HM215349	KT427715	MK169962	MN516417	MT194127	MT861911
HM215350	KT427716	MK169963	MN516418	MT194128	MT861912
HM215351	KT427717	MK169964	MN516419	MT194129	MT861913
HM215352	KT427718	MK169965	MN516420	MT194130	MT861914
HM215354	KT427719	MK169966	MN516423	MT194131	MT861915
HM215355	KT427720	MK169967	MN516424	MT194132	MT861916
HM215359	KT427721	MK169968	MN516425	MT194134	MT861917
HM215360	KT427722	MK169969	MN516426	MT194135	MT861919
HM215361	KT427723	MK169970	MN516427	MT194136	MT861920
HM215362	KT427724	MK169971	MN516428	MT194137	MT861921
HM215363	KT427725	MK169972	MN516429	MT194138	MT861922
HM215365	KT427726	MK169973	MN516430	MT194139	MT861923
HM215367	KT427727	MK169974	MN516431	MT194140	MT861924
HM215369	KT427728	MK169975	MN516432	MT194142	MT861925
HM215370	KT427729	MK169976	MN516433	MT194143	MT861926
HM215371	KT427730	MK169977	MN516434	MT194144	MT861927
HM215372	KT427731	MK169978	MN516435	MT194145	MT861928
HM215373	KT427732	MK169979	MN516436	MT194146	MT861929
HM215375	KT427733	MK169980	MN516437	MT194147	MT861930
HM215376	KT427734	MK169981	MN516438	MT194148	MT861931
HM215377	KT427735	MK169982	MN516439	MT194149	MT861932
HM215378	KT427736	MK169983	MN516440	MT194150	MT861933
HM215379	KT427737	MK169984	MN516441	MT194151	MT861934
HM215380	KT427738	MK169985	MN516442	MT194152	MT861935
HM215382	KT427739	MK169986	MN537434	MT194153	MT861936
HM215383	KT427740	MK169987	MN564837	MT194154	MT861937
HM215384	KT427741	MK169988	MN565588	MT194155	MT861938
HM215385	KT427742	MK169989	MN565589	MT194156	MT861939
HM215386	KT427743	MK169990	MN565590	MT194157	MT861940
HM215388	KT427744	MK169991	MN565591	MT194158	MT861941
HM215389	KT427745	MK169992	MN565592	MT194159	MT861942
HM215392	KT427746	MK169993	MN565593	MT194160	MT861943
HM215393	KT427747	MK169994	MN565594	MT194161	MT861944
HM215394	KT427748	MK169995	MN565595	MT194162	MT861945
HM215397	KT427749	MK169996	MN590229	MT194163	MT861946
HM215398	KT427750	MK169997	MN593212	MT194164	MT861947
HM215399	KT427751	MK169998	MN593213	MT194165	MT861948
HM215400	KT427752	MK169999	MN593214	MT194166	MT861949
HM215401	KT427753	MK170000	MN593215	MT194167	MT861950
HM215402	KT427754	MK170001	MN611462	MT194168	MT861951
HM215403	KT427755	MK170002	MN611463	MT194169	MT861952
HM215404	KT427756	MK170003	MN611464	MT194170	MT861953
HM215405	KT427757	MK170004	MN611465	MT194171	MT861954
HM215406	KT427758	MK170005	MN611466	MT194172	MT861955
HM215407	KT427759	MK170006	MN611467	MT194173	MT861956

HM215408	KT427760	MK170007	MN611468	MT194174	MT861957
HM215409	KT427761	MK170008	MN617361	MT194175	MT861958
HM215410	KT427762	MK170009	MN617362	MT194176	MT861959
HM215411	KT427763	MK170010	MN617363	MT194177	MT861960
HM215413	KT427764	MK170011	MN617364	MT194178	MT861961
HM215414	KT427765	MK170012	MN617365	MT194179	MT861962
HM215415	KT427766	MK170013	MN617367	MT194180	MT861963
HM215416	KT427767	MK170014	MN617368	MT194181	MT861964
HM215417	KT427768	MK170015	MN617369	MT194182	MT861965
HM215418	KT427769	MK170016	MN617371	MT194183	MT861966
HM215419	KT427770	MK170017	MN617372	MT194184	MT861967
HM215420	KT427771	MK170018	MN617373	MT194185	MT861968
HM215421	KT427772	MK170019	MN617374	MT194186	MT861969
HM215422	KT427774	MK170020	MN617375	MT194187	MT861970
HM215423	KT427775	MK170021	MN617376	MT194188	MT861971
HM215424	KT427776	MK170022	MN617377	MT194189	MT861972
HM215425	KT427777	MK170023	MN617378	MT194190	MT861973
HM215427	KT427778	MK170024	MN617379	MT194191	MT861974
HM215428	KT427779	MK170025	MN617380	MT194192	MT861975
HM215429	KT427780	MK170026	MN617381	MT194193	MT861976
HM215431	KT427781	MK170027	MN617382	MT194194	MT861977
HM215432	KT427782	MK170028	MN617383	MT194195	MT861978
HM215433	KT427783	MK170029	MN617384	MT194196	MT861979
HM215434	KT427784	MK170030	MN617385	MT194197	MT861980
HM215435	KT427785	MK170031	MN617386	MT194198	MT861981
HM215436	KT427786	MK170032	MN617387	MT194199	MT861982
HM234502	KT427787	MK170033	MN617388	MT194200	MT861983
HM573466	KT427788	MK170034	MN617389	MT194201	MT861984
HM586187	KT427789	MK170035	MN617390	MT194202	MT861985
HM586193	KT427790	MK170036	MN617391	MT194203	MT861986
HM586198	KT427791	MK170037	MN617392	MT194204	MT861987
HM586206	KT427792	MK170038	MN617393	MT194205	MT861988
HM586210	KT427793	MK170039	MN617394	MT194206	MT861989
HM598635	KT427794	MK170040	MN635324	MT194207	MT861990
HM623548	KT427795	MK170041	MN635325	MT194208	MT861991
HM623549	KT427796	MK170042	MN635326	MT194209	U04908
HM623550	KT427797	MK170043	MN635327	MT194210	U08805
HM623551	KT427799	MK170044	MN635328	MT194211	U09664
HM623552	KT427800	MK170045	MN635329	MT194212	U12036
HM623553	KT427801	MK170046	MN635330	MT194213	U23487
HM623554	KT427802	MK170047	MN635331	MT194214	U36859
HM623555	KT427803	MK170048	MN635332	MT194215	U36865
HM623556	KT427804	MK170049	MN635333	MT194216	U36866
HM623557	KT427805	MK170050	MN635334	MT194217	U36867
HM623558	KT427806	MK170051	MN635335	MT194218	U36869
HM623559	KT427807	MK170052	MN635336	MT194219	U36870
HM623560	KT427808	MK170053	MN635337	MT194220	U36875

HM623561	KT427809	MK177825	MN635338	MT194221	U36877
HM623562	KT427810	MK177826	MN635339	MT194222	U36879
HM623563	KT427811	MK177827	MN635340	MT194223	U36880
HM623564	KT427813	MK177829	MN635341	MT194224	U36882
HM623566	KT427814	MK205753	MN635342	MT194225	U36884
HM623567	KT427815	MK214316	MN635343	MT194227	U36885
HM623568	KT427816	MK254637	MN635344	MT194228	U36886
HM623569	KT427817	MK254639	MN635345	MT194229	U36887
HM623570	KT427818	MK258683	MN635346	MT194230	U39233
HM623571	KT427819	MK258684	MN635347	MT194232	U39234
HM623572	KT427820	MK272339	MN635348	MT194233	U39237
HM623573	KT427822	MK272340	MN635349	MT194234	U39239
HM623574	KT427823	MK272341	MN635350	MT194235	U39240
HM623575	KT427824	MK272342	MN635351	MT194236	U39241
HM623576	KT427825	MK272343	MN635352	MT194237	U39244
HM623577	KT427826	MK272344	MN635353	MT194238	U39245
HM623579	KT427827	MK272345	MN635354	MT194239	U39250
HM623580	KT427828	MK272346	MN635355	MT194240	U39253
HM623581	KT427829	MK272347	MN635356	MT194242	U39255
HM623582	KT427830	MK272348	MN635357	MT194243	U39258
HM623583	KT427831	MK272349	MN635358	MT194244	U39362
HM623584	KT427841	MK272350	MN635359	MT194245	U43096
HM623585	KT427847	MK272351	MN635360	MT194246	U43141
HM623586	KT427849	MK272352	MN635361	MT194247	U46016
HM623587	KT427853	MK272353	MN635362	MT194248	U48264
HM623589	KT427860	MK272354	MN635363	MT194249	U48266
HM623590	KT427861	MK272355	MN635364	MT194250	U51188
HM623591	KT427866	MK272356	MN635365	MT194251	U51189
HM623592	KT427868	MK272357	MN635366	MT194252	U52953
HM623593	KT427869	MK272358	MN635367	MT194253	U65075
HM623595	KT427870	MK272359	MN635368	MT194254	U69584
HM623596	KT427871	MK272360	MN635369	MT194255	U71182
HM623597	KT427872	MK272361	MN635370	MT194256	U82990
HM623598	KT427873	MK272362	MN635371	MT194257	U82991
HM623599	KT438782	MK272363	MN635372	MT194258	U82992
HM623600	KT438783	MK272364	MN635373	MT194259	U82993
HM623601	KT438784	MK272365	MN635374	MT194260	U84819
HM623602	KT452084	MK272366	MN635375	MT194261	U84854
HM623603	KT452108	MK272367	MN635376	MT194262	U86778
HM623604	KT452143	MK272368	MN635377	MT194263	U88822
HM623605	KT452191	MK272369	MN635378	MT194264	U88823
HM623606	KT452215	MK272370	MN635379	MT194265	U88825
HM623607	KT452239	MK272371	MN635380	MT194266	U88826
HM623608	KT452263	MK272372	MN635381	MT194267	U90934
HM623609	KT452311	MK272373	MN635382	MT194268	X04415
HM623610	KT452335	MK272374	MN635383	MT194269	X96522
HM623611	KT452359	MK272375	MN635384	MT194270	X96526

HM638460	KT452407	MK272376	MN635385	MT194271	Y13717
HM638516	KT452455	MK272377	MN635386	MT194272	Y13718
HM638548	KT452479	MK272379	MN635387	MT194273	Y13719
HM638583	KT452503	MK272380	MN635388	MT194274	
HM638616	KT452527	MK272381	MN635389	MT194275	
HM638668	KT452563	MK272382	MN635390	MT194276	
HM638722	KT452599	MK272383	MN635391	MT194277	
HM638780	KT592380	MK272384	MN635392	MT194278	
HM638826	KT619126	MK272385	MN635393	MT194279	
HM638888	KT808396	MK272386	MN635394	MT194280	
HM638963	KT878021	MK272387	MN635395	MT194281	
HM639009	KT878022	MK272388	MN635396	MT194282	
HM639073	KT878023	MK272389	MN635397	MT194283	
